# Chitosan–Unconjugated Bilirubin Microspheres Alleviate Dysbiosis, Immune Dysregulation, and Intestinal Barrier Damage in Ulcerative Colitis

**DOI:** 10.3390/biom16081140

**Published:** 2026-08-05

**Authors:** Xinyu Lyu, Xiaotong Xu, Mengqi Shi, Rui Wang, Xiaoqing Yu, Yan Liu, Xiangyu Xue, Fengmin Zhang, Xiuhong Wang

**Affiliations:** 1 Department of Microbiology, School of Basic Medical Sciences, WU Lien-Teh Institute, Harbin Medical University, Harbin 150081, China; 202501020@hrbmu.edu.cn (X.L.);; 2 Department of Biochemistry and Molecular Biology, School of Basic Medical Sciences, Harbin Medical University, Harbin 150081, China; 3 Heilongjiang Provincial Key Laboratory of Infection and Immunity, Harbin Medical University, Harbin 150081, China; 4 Department of Ultrasound, Harbin Medical University Cancer Hospital, Harbin 150081, China

**Keywords:** ulcerative colitis, unconjugated bilirubin, chitosan, microspheres, gut microbiota, digestive proteases

## Abstract

Ulcerative colitis (UC) is a chronic inflammatory bowel disease marked by immune dysregulation, microbiota imbalance, and intestinal barrier damage. Unconjugated bilirubin (UCB) shows promise in treating UC due to its anti-inflammatory properties but is limited by poor solubility and potential toxicity. This study developed a chitosan-based controlled-release microsphere (CBMS) treatment to overcome these issues. CBMS utilizes the mucoadhesive properties of chitosan to achieve targeted, controlled UCB release in the colon, enhancing its stability and bioavailability. In a DSS-induced UC mouse model, CBMS alleviated clinical symptoms, reduced colon shortening, and improved histological outcomes, including reduced inflammation and enhanced mucosal repair. The mechanism involves CBMS retention in the intestinal lumen, UCB inactivation of digestive proteases, and restoration of microbiota balance, suppressing pro-inflammatory pathways. CBMS offers a promising new therapeutic strategy for UC and insights into polysaccharide-based drug delivery systems.

## 1. Introduction

UCB is a key product of heme metabolism, generated by the action of heme oxygenase (HO) which catalyzes the conversion of heme to biliverdin, followed by reduction to UCB by biliverdin reductase [1,2]. Recent clinical and basic research has increasingly confirmed that UCB holds considerable potential in the prevention and treatment of various diseases, particularly immune-mediated conditions such as inflammatory bowel disease (IBD) [3,4]. Studies have shown a significant negative correlation between serum bilirubin levels and the risk and activity of IBD, suggesting that UCB may play a critical role in the pathogenesis and progression of IBD through its anti-inflammatory and immunomodulatory effects [5]. Our previous studies also demonstrated that UCB protects the intestinal mucosa by improving epithelial tight junctions, alleviating oxidative stress and inflammation, regulating immune cell activation, and inhibiting the activity of aberrant digestive enzymes [4]. Thus, UCB is considered a promising endogenous protective factor with significant clinical value in the treatment of IBD.

However, despite its substantial therapeutic potential, the clinical application of UCB faces several technical challenges. First, UCB is highly hydrophobic and unstable in aqueous solutions. Second, UCB is prone to oxidative degradation in vivo, and its high accumulation may cause adverse effects such as jaundice and neurotoxicity, limiting its use as a therapeutic agent [6]. To address these issues, recent research has focused on optimizing UCB delivery using nanotechnology. By encapsulating or chemically modifying UCB with nanocarriers, its stability, solubility, targeting ability, and bioavailability can be significantly improved, enhancing its clinical prospects [7].

In drug delivery, CIS has gained widespread use due to its excellent biocompatibility, biodegradability, and cationic properties [8]. CIS and its derivatives can strongly adsorb to the intestinal mucosa through electrostatic interactions, prolonging drug retention in the gut and improving bioavailability [9]. Several studies have shown that CIS-based UCB nanocarriers not only enhance UCB stability but also protect it from oxidative degradation while promoting its anti-inflammatory effects by increasing its local concentration. Moreover, these nanodelivery systems have shown significant anti-inflammatory, mucosal repair, and immunomodulatory effects in mouse models of colitis, liver fibrosis, and gastric ulcers [10,11,12,13,14].

In our previous studies, we found that UCB could effectively inactivate digestive proteases, providing a new strategy for its application in intestinal disease treatment. Thus, we propose an innovative CBMS treatment as a novel gut-targeted delivery platform. Due to its larger particle size, CBMS exhibits stronger adhesion in the intestinal lumen, which prolongs its local retention time, reduces the chance of penetration through the intestinal wall into systemic circulation, and minimizes the risk of systemic side effects. We hypothesize that the micron-sized particles allow sustained UCB release locally in the gut, offering prolonged therapeutic effects. Specifically, CBMS demonstrated significant therapeutic effects in a mouse model of ulcerative colitis (UC), alleviating intestinal mucosal inflammation, repairing gut barrier function, and reducing digestive enzyme activity in the gut, further promoting the restoration of immune function. Additionally, CBMS has the unique advantage of restoring the balance of the gut microbiota, thereby enhancing its effectiveness in IBD treatment. The unique properties of micron-sized CBMS provide broader application potential in suppressing inflammation, restoring intestinal barrier function, and modulating the immune microenvironment.

In summary, we have designed an innovative CBMS treament as a new gut-targeted delivery system that overcomes the limitations of traditional nanocarriers. By optimizing UCB delivery and local release, CBMS not only offers an effective treatment for IBD but also opens up new approaches and strategies for treating other inflammation-driven, immune-mediated diseases.

## 2. Materials and Methods

### 2.1. Experimental Animals

Male C57BL/6 mice (18–22 g; 10 weeks old) were obtained from Liaoning Changsheng Biotechnology Co., Ltd. (Benxi, Liaoning, China). All experimental protocols involving mice were approved by the Animal Ethics and Welfare Committee of Harbin Medical University, China (Approval No.: HMUIRB2023003). All animal experiments were conducted in accordance with the NIH Guide for the Care and Use of Laboratory Animals (NIH Publication No. 85-23, Revised 1996).

### 2.2. Chemicals and Reagents

UCB (unconjugated bilirubin, purity > 95%, UV-Spec) was purchased from Lee Biosolutions Inc. (Maryland Heights, MO, USA). DSS (dextran sulfate sodium salt, colitis grade, molecular weight of 36,000–50,000 Da) was purchased from MP Biomedicals Inc. (Solon, OH, USA). CIS (chitosan, average molecular weight = 200 kDa, degree of deacetylation ≥ 85%) was purchased from Aladdin Bio-Chem Technology Co., Ltd. (Shanghai, China). BAPNA (N-Benzoyl-DL-arginine-4-nitroanilide hydrochloride) and BTEE (N-Benzoyl-L-tyrosine ethyl ester) were purchased from Sigma-Aldrich Co. (St. Louis, MO, USA). PNPG (4-Nitrophenyl β-D-glucopyranoside) was purchased from BBI Life Sciences (Shanghai, China). A fecal DNA extraction kit was purchased from GBCBIO Technologies Inc. (Guangzhou, China). ELISA kits for IL-6 and LPS were purchased from Jiang Lai Bio. (Guangzhou, China). The antibodies used in this study included anti-Occludin (YN2865, Immunoway, Plano, TX, USA), anti-Claudin-1 (YT0942, Immunoway, Plano, TX, USA), anti-phospho-NF-κB p65 (YP0959, Immunoway, Plano, TX, USA), anti-E-cadherin (YT1454, Immunoway, Plano, TX, USA), anti-NF-κB (YM3111, Immunoway, Plano, TX, USA), anti-TRAF6 (YT4720, Immunoway, Plano, TX, USA), anti-IL-17 (YT7835, Immunoway, Plano, TX, USA), anti-β-catenin (D10A8, Cell Signaling Technology, Danvers, MA, USA), anti-Wnt3a (29225, SAB, College Park, MD, USA), anti-IL-10 (WL03088, Wanleibio, Shenyang, China), anti-IL-6 (WL0284, Wanleibio, Shenyang, China), anti-β-actin (81115-1-RR, Proteintech, Rosemont, IL, USA), HRP-conjugated goat anti-rabbit IgG (H + L) (RS0002, Immunoway, Plano, TX, USA), and HRP-conjugated goat anti-mouse IgG (H + L) (RS0001, Immunoway, Plano, TX, USA). All reagents used in this study were of analytical reagent grade.

### 2.3. Preparation of CBMS

CBMS was prepared by an emulsification and cross-linking method using CIS and UCB as the primary raw materials. Briefly, 2 mL of glacial acetic acid was diluted to 100 mL with distilled water, followed by the addition of 2.0 g of CIS powder. The mixture was stirred until complete dissolution. Subsequently, 0.4 g of UCB was added and dissolved completely under stirring. In this experiment, the mass ratio of UCB to CIS was maintained at m1(UCB):m2(CIS) = 1:5. After complete dissolution of UCB and CIS, 20 mL of the acetate–UCB–CIS mixed solution was slowly added dropwise into a liquid paraffin phase containing 100 mL of liquid paraffin and 3 mL of Span 80 emulsifier. During the emulsification process, the mixture was stirred at 1000 rpm for 60 min. Subsequently, 0.5 mL of formaldehyde was added, and stirring was continued for another 30 min. Then, 2 mL of glutaraldehyde solution (50%) was added to the emulsion system, followed by continuous stirring for 180 min to complete the cross-linking reaction. After centrifugation at 2000 rpm for 10 min, the oil phase was collected, and the obtained microspheres were sequentially washed with anhydrous ethanol, chloroform, and anhydrous ethanol for 5 min each. The product was then freeze-dried using a freeze dryer (Biosafer, Nanjing, China) for 24 h and stored at −20 °C. The entire preparation process was performed under light-protected conditions and maintained at 4 °C.

### 2.4. Preparation of Simulated Intestinal Fluid

According to reference [10], simulated intestinal fluid was prepared by dissolving pepsin (5 μg/mL) and trypsin (30 μg/mL) in 0.1 mol/L phosphate-buffered saline (PBS). The pH was adjusted to 6.8 to simulate the intestinal environment.

### 2.5. Particle Size and Zeta Potential Analysis

The CBMS sample was dispersed in deionized water at a concentration of 0.1 mg/mL. The particle size distribution and zeta potential of CBMS were measured using a BeNano 180 Zeta Potential Analyzer (Dandong Bettersize Instruments Ltd., Dandong, China).

### 2.6. Infrared Spectroscopy Analysis

An appropriate amount of CBMS was mixed with dried KBr crystals under a heating lamp, thoroughly ground, and homogenized. The resulting mixture was compressed into pellets for FTIR analysis. FTIR spectra were recorded over the range of 4000–400 cm^−1^ using an FTIR-650 Fourier transform infrared spectrometer (Shimadzu, Kyoto, Japan), with KBr used as the background reference. The characteristic absorption bands were identified and analyzed.

### 2.7. Microstructural Morphology Characterization

The morphology of CBMS was characterized using a field-emission scanning electron microscope (FE-SEM, HT7700, JEOL Ltd., Tokyo, Japan) operated at an accelerating voltage of 5.0 kV. Prior to observation, the CBMS sample was uniformly mounted onto the specimen stage and sputter-coated with a thin layer of gold. SEM images were acquired at magnifications of 700× and 1000× to evaluate the surface morphology of CBMS.

### 2.8. The Establishment of Experimental Animal Models

In this experiment, mice were randomly divided into five groups: the Control group, DSS-induced colitis model group (DSS group), DSS + CIS treatment group (DSS + CIS group), DSS + UCB treatment group (DSS + UCB group), and DSS + CBMS treatment group (DSS + CBMS group), with 6–8 mice per group. Except for the Control group, which received sterile water, all other groups were administered 3% DSS aqueous solution through drinking water for seven consecutive days to induce acute colitis. From day 1 to day 7, treatments were administered by oral gavage at the same time each day. The DSS group received 200 μL of PBS, the CIS group received 200 μL of 0.5% CIS solution, the UCB group received 200 μL of UCB solution (400 μmol/L), and the CBMS group received 200 μL of a mixture containing 0.5% CIS solution and an appropriate amount of CBMS. The amount of UCB encapsulated in CBMS was adjusted to be approximately equivalent to the UCB dose administered in the UCB group. Body weight, stool consistency, and fecal occult blood were monitored and recorded throughout the experimental period. On day 8, all mice were euthanized after anesthesia with 1% sodium pentobarbital solution via intraperitoneal injection (0.1 mL/10 g body weight). The spleen, colon, rectum, and intestinal contents were collected and stored at −80 °C for subsequent analyses.

### 2.9. Disease Activity Index

According to the calculation criteria of weight loss, stool consistency and rectal bleeding listed in Table S1, the disease activity index (DAI) of each animal was calculated.

### 2.10. Histology Analysis for Scoring Colonic Damage and Inflammation

Colon tissues fixed in Carnoy’s solution were embedded in paraffin and sectioned into 5 μm thick slices. The sections were stained with hematoxylin and eosin (H&E) and examined under a light microscope. The extent of colonic injury and inflammation was evaluated using a blinded scoring method according to the criteria described in Table S2.

### 2.11. Immunofluorescence

After deparaffinization, the paraffin sections were blocked with 5.0% FBS for 30 min and incubated with primary antibodies at 4 °C overnight. After washing with PBS, the sections were incubated with fluorescently labeled secondary antibodies in the dark for 1.5 h. The nuclei were then counterstained with DAPI. Non-specific background signals were corrected using the “Subtract Background” function in ImageJ software (version 1.54p). All images were acquired and processed using the same parameters (Olympus, Tokyo, Japan).

### 2.12. Determination of Fecal Digestive Protease Activity and β-Glucuronidase (β-GD) Activity

Fecal digestive proteases (trypsin and chymotrypsin) activities were measured by using BAPNA (N-Benzoyl-DL-arginine-4-nitroanilide hydrochloride) and BTEE (N-Benzoyl-L-tyrosine ethyl ester) as the substrate [13]. The activity of β-GD was tested as a substrate for PNPG (4-Nitrophenyl β-D-glucopyranoside) as previously described [15].

### 2.13. Extraction of Fecal Total DNA and 16S rRNA Diversity Sequencing

Fecal microbial DNA was extracted from stool samples using the Stool DNA Kit (GBCBIO Technologies Inc., Guangzhou, China) according to the manufacturer’s instructions. The extracted fecal DNA samples were submitted to Biozeron Biotechnology Co., Ltd. (Shanghai, China) for microbial community diversity analysis using Illumina sequencing.

### 2.14. Quantitative Real-Time PCR (RT-qPCR) Analysis

Total RNA was extracted from colon tissues, and cDNA was synthesized using a reverse transcription kit (TransGen Biotech Co., Ltd., Beijing, China) according to the manufacturer’s instructions. Reverse transcription was performed using a SimpliAmp^™^ PCR system (Applied Biosystems, Foster City, CA, USA). Quantitative real-time PCR was conducted using a TransStart^®^ Top Green qPCR SuperMix kit (TransGen Biotech Co., Ltd., Beijing, China) on a CFX Connect Real-Time PCR Detection System (Bio-Rad Laboratories, Hercules, CA, USA) with a final reaction volume of 20 μL.

All primers were synthesized by Invitrogen Trading Co., Ltd. (Shanghai, China), and the primer sequences are listed in Supplementary Table S3. Each sample was analyzed in triplicate, and gene expression levels were normalized to GAPDH. Relative expression levels were calculated using the 2^−ΔΔCt^ method.

### 2.15. Western Blot Analysis

Colon tissues were homogenized in RIPA lysis buffer containing PMSF at a ratio of 100:1 to extract total proteins. The lysates were incubated on ice for 30 min and centrifuged at 12,000 rpm for 15 min at 4 °C. The supernatants were collected as protein samples. Protein concentrations were determined using a BCA Protein Assay Kit (Beyotime Biotechnology, Shanghai, China).

Equal amounts of protein samples were prepared and standardized to 400 μg of total protein in a final volume of 100 μL. Western blot analysis was performed to evaluate the expression levels of target proteins. Briefly, proteins were separated by 10% sodium dodecyl sulfate-polyacrylamide gel electrophoresis (SDS-PAGE) and transferred onto polyvinylidene difluoride (PVDF) membranes. The membranes were incubated with primary antibodies against β-actin, IL-17A, TRAF6, NF-κB, phosphorylated NF-κB (p-NF-κB), IL-6, IL-10, Wnt3a, β-catenin, Claudin-1, E-cadherin, and Occludin. Subsequently, the membranes were incubated with horseradish peroxidase (HRP)-conjugated goat anti-rabbit or goat anti-mouse secondary antibodies.

Protein bands were visualized using an Amersham Imager 600 system (GE Healthcare, Chicago, IL, USA), and band intensities were quantified using ImageJ software (version 1.54p).

### 2.16. ELISA Analysis

Serum NO, IL-6 and LPS levels were determined by ELISA kit (Jianglai Bio., Guangzhou, China) according to manufacturer’s instructions.

### 2.17. Statistical Analysis

All data were presented as mean ± SD and were analyzed with GraphPad Prism 8.2.1 software. Differences among groups were analyzed using one-way ANOVA followed by Tukey’s multiple comparisons test. The results were considered statistically significant when *p* values are less than 0.05.

## 3. Results

### 3.1. Characterization and Morphology of CBMS

**Figure 1:**
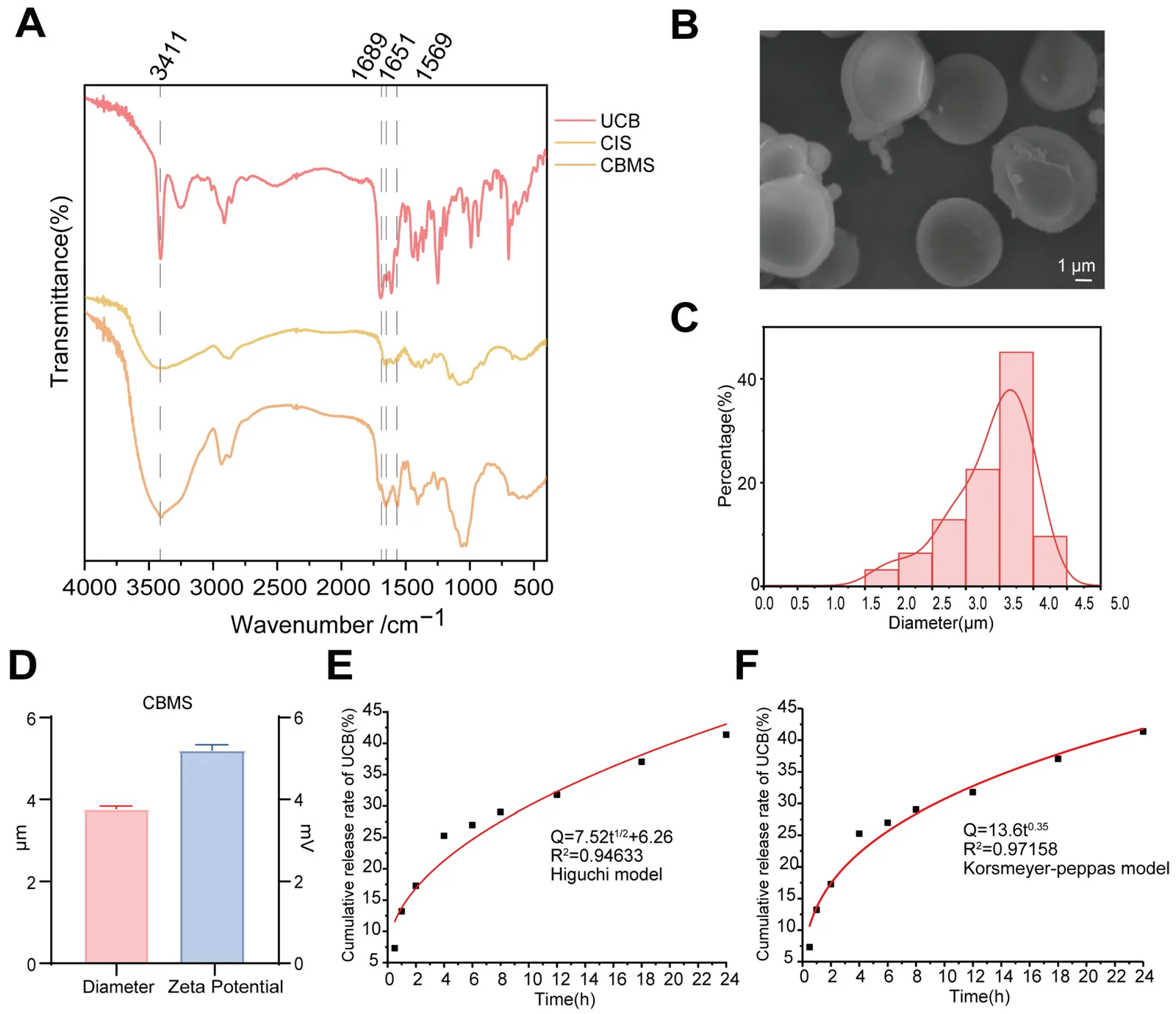
Characteristics of CBMS. (**A**) FITR of UCB (red), CIS (yellow) and CBMS (orange). (**B**) SEM image of CBMS. (**C**) Size distribution of CBMS. (**D**) Zeta potential of CBMS. (**E**) The curve of the Korsmeyer–Peppas kinetics model of UCB release. (**F**) The curve of the Higuchi kinetics model of UCB release. Data represent means ± SD. *n* = 3.

We successfully developed an innovative CBMS as a novel intestinal-targeted delivery material using an emulsification–crosslinking method. This material is designed to provide a micro-sized bilirubin carrier suitable for local retention and delivery in the intestinal lumen, thereby achieving more effective therapeutic outcomes. FT-IR spectral analysis results (Figure 1A) revealed that the FT-IR spectrum of CIS exhibited a broad and intense absorption peak at approximately 3430 cm^−1^, corresponding to O-H and N-H stretching vibrations [16]. In the spectrum of UCB, a prominent C=O stretching vibration peak was observed around 1700 cm^−1^, and a pyrrole ring vibration absorption was visible in the 1600–1400 cm^−1^ range [17]. For CBMS, the FT-IR spectrum showed a sharp strong peak at 1689 cm^−1^, which corresponds to the C=N stretching vibration of the Schiff base bond formed by the condensation of glutaraldehyde with the amino groups of CIS, directly confirming the occurrence of covalent crosslinking. Additionally, the intensity of the amide II band at 1569 cm^−1^ was significantly enhanced, and the peak shape broadened. This was attributed not only to the acetylamino contribution of the original CIS but also to the formation of amide bonds (-CONH-) between the carboxyl group of UCB and the amino group of CIS through an amidation reaction, indicating that UCB was covalently attached to the CIS network. These changes suggest that UCB and CIS formed a composite with a covalent structure, rather than just a simple physical mixture.

SEM images showed that CBMSs had smooth surfaces, a regular shape, uniform size, and exhibited excellent spherical morphology (Figure 1B). Further Dynamic Light Scattering (DLS) particle size distribution measurements (Figure 1C) indicated that the average particle size of CBMS was 3.772 ± 0.664 μm, with a polydispersity index (PDI) of 0.60 ± 0.08, confirming that CBMS forms relatively uniform micron-sized aggregates in the aqueous phase. Zeta potential measurements showed that the Zeta potential of CBMS was 5.19 ± 0.13 mV (Figure 1D). The drug encapsulation rate was 15.2%, with a high loading efficiency of 91.2% (Figure S1). In simulated intestinal fluid (SIF), the in vitro release curve showed that the cumulative release rate of UCB from CBMS reached 41.37% after 24 h, indicating that the drug delivery system exhibited significant sustained-release characteristics. To further elucidate the potential mechanism of drug release, four mathematical models were used to analyze the release kinetics: zero-order kinetics, first-order kinetics, the Higuchi model, and Korsmeyer–Peppas model. According to the correlation coefficients (R^2^) in Table S4, the release behavior of CBMS most closely followed the Korsmeyer–Peppas and Higuchi models (Figure 1E,F). The consistency of these two models indicates that the release of UCB is primarily diffusion-controlled and predominantly governed by the Fickian diffusion mechanism through the CIS matrix transport process. These results suggest that CBMS effectively encapsulates and protects the hydrophobic UCB, enabling long-lasting and controlled drug release in a simulated intestinal fluid environment.

### 3.2. CBMS Alleviates Symptoms in DSS-Induced UC Mice

**Figure 2:**
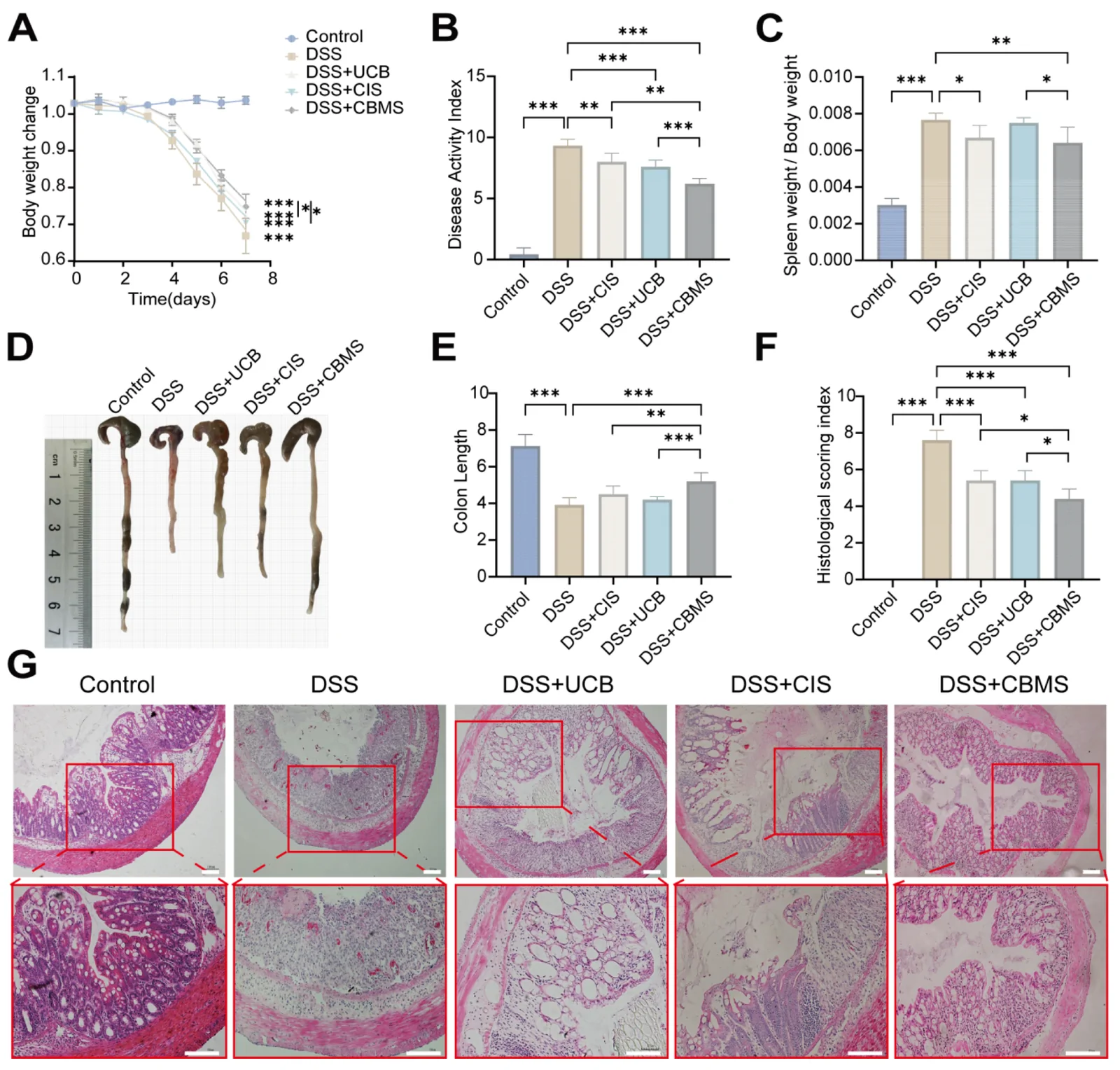
CBMS ameliorates disease symptoms and intestinal damage in DSS-induced colitis. (**A**) Body weight changes. (**B**) DAI scores. (**C**) Spleen index. (**D**) Representative colon images. (**E**) Colon length quantification. (**F**) Histopathological scoring. (**G**) H&E-stained colon sections. Scale bar: 55 μm. Significance: * *p* < 0.05, ** *p* < 0.01, *** *p* < 0.001, *n* ≥ 3.

We evaluated the therapeutic potential of CBMS in an acute colitis mouse model. The treatment effects of CBMS on DSS-induced colitis were assessed using multiple indices, including body weight, the disease activity index (DAI) score, the spleen index, colon length, and histological damage. These parameters effectively reflect the severity of inflammation. The DSS-induced colitis mouse model is characterized by weight loss. Mice were treated with different formulations for seven days while being simultaneously administered DSS, with daily body weight monitoring (Figure 2A). The Control group mice showed continuous weight gain, while the DSS group exhibited persistent weight loss, accompanied by diarrhea and other symptoms, confirming that DSS induces severe colitis in the mice and successfully establishes the animal model. Compared to UCB and CIS, CBMS treatment significantly alleviated weight loss. Additionally, the DAI score of the mice was evaluated based on body weight, stool consistency, and fecal occult blood (Figure 2B, Table S1). The DAI score of the DSS group mice was significantly elevated, indicating the severity of induced colitis. CBMS-treated mice showed a significant reduction in the DAI score, and the therapeutic effect of CBMS was significantly better than that of UCB and CIS. The spleen index, a marker of systemic immune activation, was significantly increased in the DSS-induced colitis model group compared to the Control group, reflecting notable immune system activation. After CBMS treatment, the spleen index of the mice significantly decreased, with CBMS showing a more pronounced inhibitory effect compared to UCB and CIS (Figure 2C). DSS-induced colitis often involves compensatory hypertrophy of the colon tissue, leading to significant thickening of the colon wall and shortening of the colon length. Therefore, colon shortening was used as an indirect indicator of colitis. The colon length of DSS group mice was significantly shorter than that of the Control group, while CBMS treatment significantly improved the symptoms, and the therapeutic effect of CBMS was superior to that of UCB and CIS (Figure 2D,E). Histological examination of CBMS’s effects on DSS-induced colitis was performed using H&E staining (Figure 2F,G, Table S2). DSS group mice showed marked signs of inflammation, including depletion of goblet cells, crypt damage, and infiltration of inflammatory cells into the colonic lamina propria. In contrast, histological features in CBMS-treated mice were significantly improved, indicating that CBMS could notably alleviate mucosal damage in DSS-induced colitis. The histological changes were quantified using a colon damage scoring system, with the DSS group exhibiting the highest score, while CBMS treatment reduced the score to near normal levels. The CCK-8 assay demonstrated that CBMS treatment for 12, 24, and 48 h caused no detectable cytotoxicity compared with the Control group (Figure S2). Compared to the Control group, there were no significant changes in the serum levels of alanine aminotransferase (ALT), aspartate aminotransferase (AST), blood urea nitrogen (BUN), or creatinine (CREA) in the CBMS-treated mice (Figure S3). Additionally, H&E staining of major organs showed no signs of pathological damage, such as structural abnormalities or inflammation (Figure S4). These results further support the biosafety and biocompatibility of CBMS.

### 3.3. CBMS Reduces the Activity of Digestive Proteases in the Intestinal Lumen of DSS-Induced UC Mice

In addition to the gut microbiota, digestive proteases such as trypsin and chymotrypsin are also present in the intestinal lumen, playing a crucial role in the degradation of dietary proteins. Under normal physiological conditions, these proteases do not cause harm to the gut. However, in germ-free animals, a significant amount of unneutralized digestive proteases has been detected, suggesting a close relationship between the gut microbiota and the inactivation of digestive proteases [18]. The enzymatic activity of proteases plays a vital role in maintaining intestinal barrier homeostasis, as excessive protease activity accelerates the degradation of mucin, leading to structural mucosal damage [4].

Bilirubin in the intestinal lumen is initially secreted in its conjugated form. It is then converted into UCB by the action of β-GD released from intestinal cells, which removes the glucuronic acid moiety. This conversion enables bilirubin to neutralize digestive proteases and reduce their ability to degrade mucins, thereby protecting the intestinal mucosa from damage [18].

**Figure 3:**
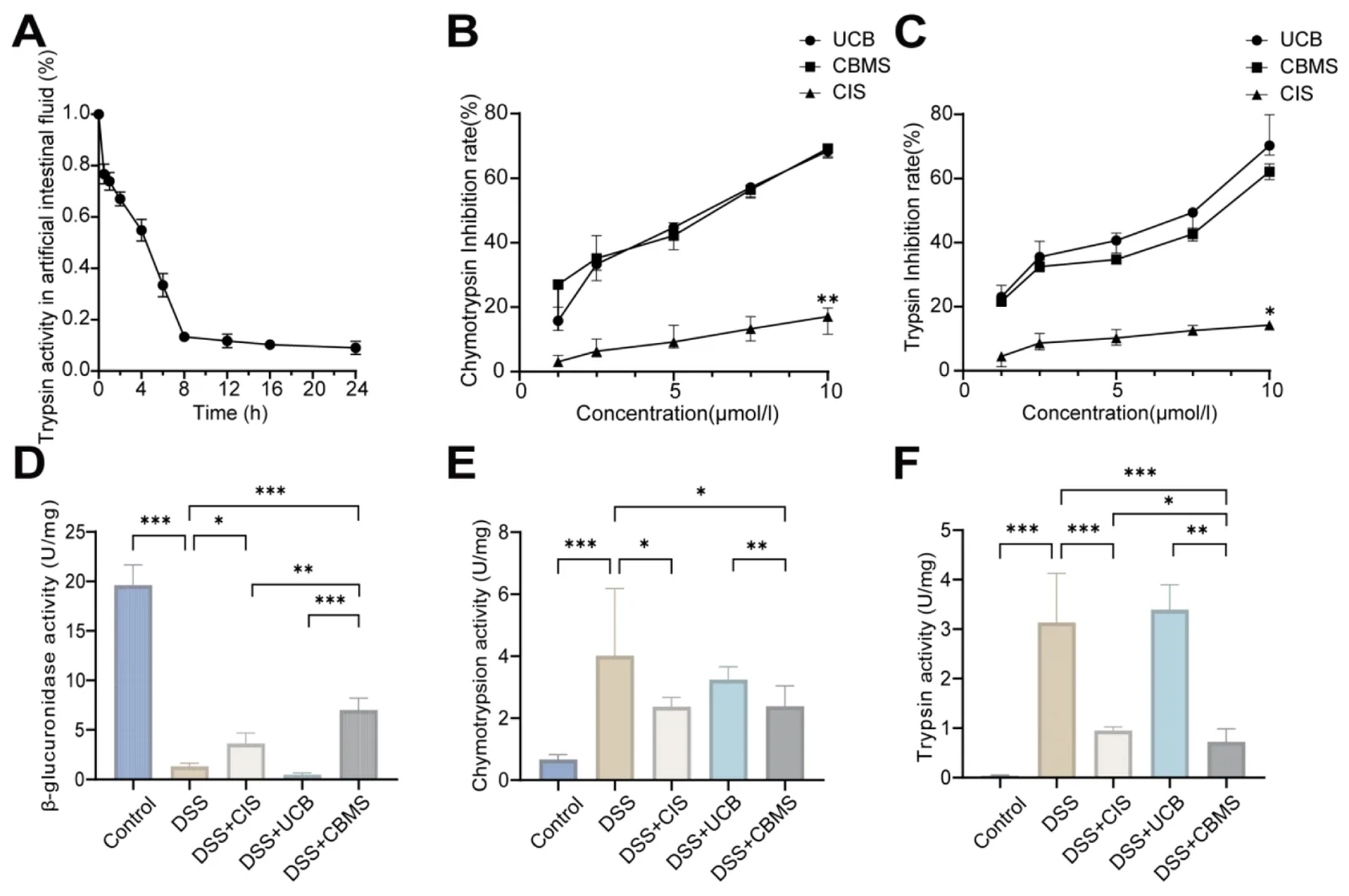
CBMS decreased the activity of intestinal lumen digestive proteases. (**A**) Inactivation of trypsin by CBMS in simulated artificial intestinal fluid. In vitro validation of the inhibitory effect of UCB and CBMS on trypsin (**B**), chymotrypsin (**C**). (The horizontal coordinate concentration values corresponding to CBMS in the figure refer specifically to the concentration of UCB in this composite system and not to the concentration parameter of CBMS.) (**D**) Fecal trypsin activity. (**E**) Fecal chymotrypsin activity. (**F**) Fecal β-GD activity. * *p* < 0.05, ** *p* < 0.01, *** *p* < 0.001. Data represent means ± SD. *n* ≥ 3.

To assess whether CBMS has the ability to inactivate digestive proteases, we evaluated its effect on trypsin in simulated artificial intestinal fluid. The results showed that after 8 h, CBMS achieved an 86.7% inactivation rate of trypsin (Figure 3A). We further evaluated the inactivation abilities of CBMS, UCB, and CIS on both trypsin and chymotrypsin in vitro, using benzoyl-L-arginine-p-nitroanilide (BAPNA) and N-benzoyl-L-tyrosine ethyl ester (BTEE) as substrates to measure their activities. The results indicated that CBMS effectively inactivated digestive proteases, demonstrating a similar efficacy to UCB and significantly outperforming CIS (Figure 3B,C). Our previous studies have shown that bacterial-derived β-GD can effectively neutralize digestive proteases. In the UC disease model, the activities of trypsin, chymotrypsin, and β-GD exhibit a negative correlation [19].

Next, we assessed the levels of digestive proteases in vivo. By measuring the activity of trypsin, chymotrypsin, and β-GD in the feces of mice, we observed that in the DSS group, the activities of trypsin and chymotrypsin were significantly elevated, while β-GD activity was markedly reduced. After CBMS treatment, the activities of trypsin and chymotrypsin were significantly decreased (Figure 3D,E), while β-GD activity was significantly increased (Figure 3F).

### 3.4. CBMS Restores the Intestinal Barrier in DSS-Induced UC Mice

Intestinal epithelial cell death and widespread epithelial erosion are significant features of IBD. The disruption of the intestinal mucosal barrier allows pathogens to directly contact intestinal epithelial cells, triggering an inflammatory response that forms a vicious cycle, further compromising the integrity of the intestinal mucosa. Intestinal stem cells located at the crypt base continuously promote the renewal of intestinal epithelial cells in mice, with the Wnt signaling pathway playing a crucial role in intestinal epithelial renewal and maintaining homeostasis [19]. Intestinal epithelial cells are interconnected through tight junction proteins, which are key components of the mucosal barrier, including ZO-1, E-cadherin, Occludin, and Claudin.

**Figure 4:**
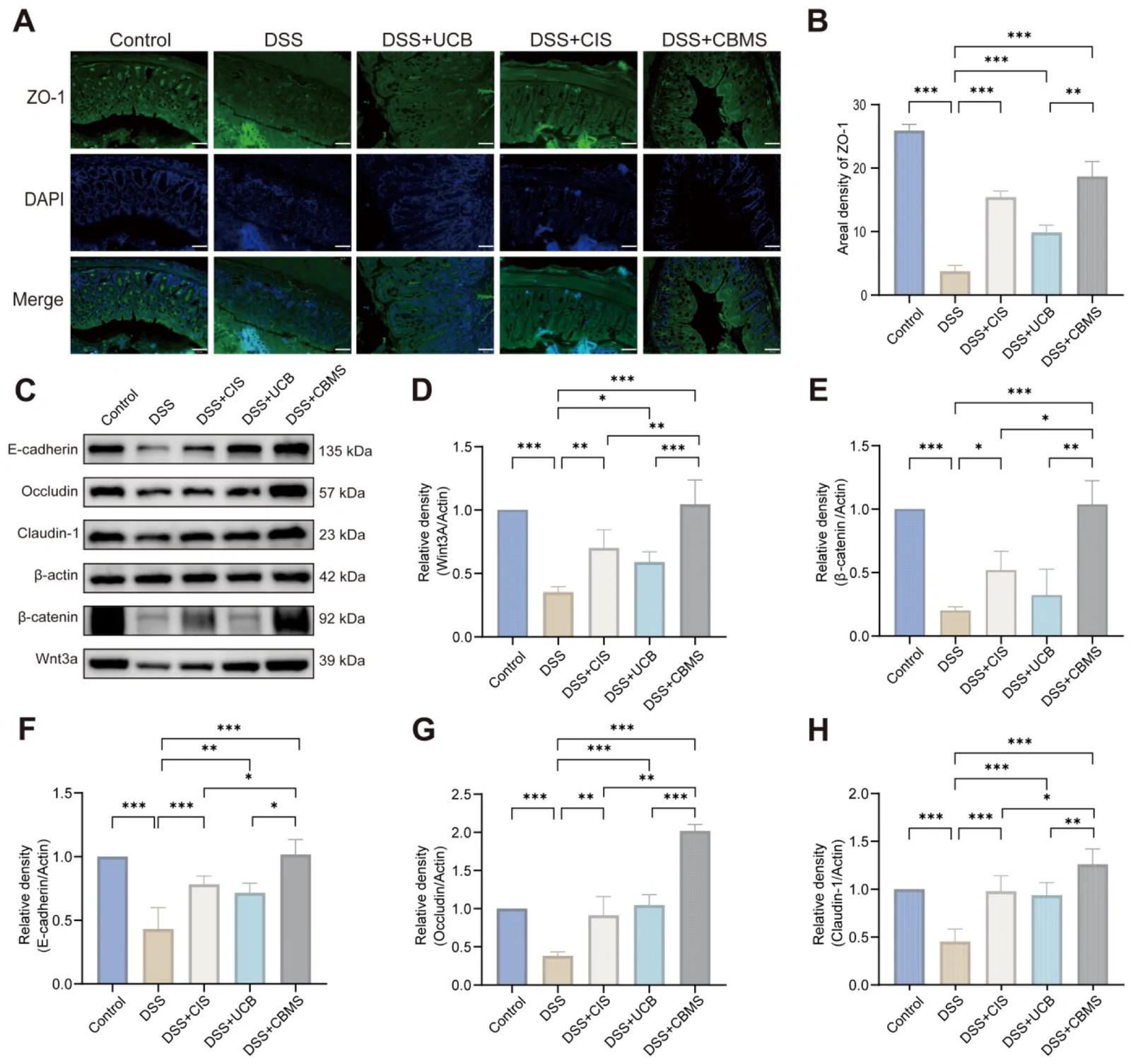
CBMS improves intestinal barriers in DSS-induced UC mice. (**A**) ZO-1 immunofluorescence (green) and Hoechst 33258 nuclear staining (blue) in colon tissues. Scale bar: 50 μm. (**B**) ZO-1 fluorescence density quantification. (**C**) Protein expression of E-cadherin, Occludin, Claudin-1, Wnt3A, and β-catenin. (**D**–**H**) Relative grayscale values of E-cadherin, Occludin, Claudin-1, Wnt3A, and β-catenin. * *p* < 0.05, ** *p* < 0.01, *** *p* < 0.001. Data represent means ± SD. *n* ≥ 3. Original uncropped Western blot images are provided in Supplementary Material S1.

To assess the distribution and expression levels of tight junctions between intestinal epithelial cells in the colon, we analyzed colon tissue sections from mice using immunofluorescence and Western blotting. Immunofluorescence results showed that in the DSS group, the distribution and fluorescence intensity of ZO-1 in the colon were significantly reduced (Figure 4A,B). Compared to the DSS group, CBMS treatment partially restored the expression level of ZO-1 in the colon, and the effect was significantly better than that of UCB and CIS. Western blot analysis further confirmed that the expression levels of Wnt3A, β-catenin, E-cadherin, Occludin, and Claudin-1 in the colon of DSS-treated mice were significantly decreased (Figure 4C–H). In contrast, in the CBMS treatment, the expression levels of these proteins were significantly increased, and the therapeutic effect of CBMS was significantly superior to that of UCB and CIS.

These results indicate that CBMS moderately activates the Wnt/β-catenin signaling pathway, promoting the renewal of intestinal epithelial cells and the proliferation of intestinal stem cells, thereby restoring tight junctions and repairing the intestinal mucosal barrier.

### 3.5. CBMS Modulates Intestinal Immune Homeostasis in DSS-Induced UC Mice

**Figure 5:**
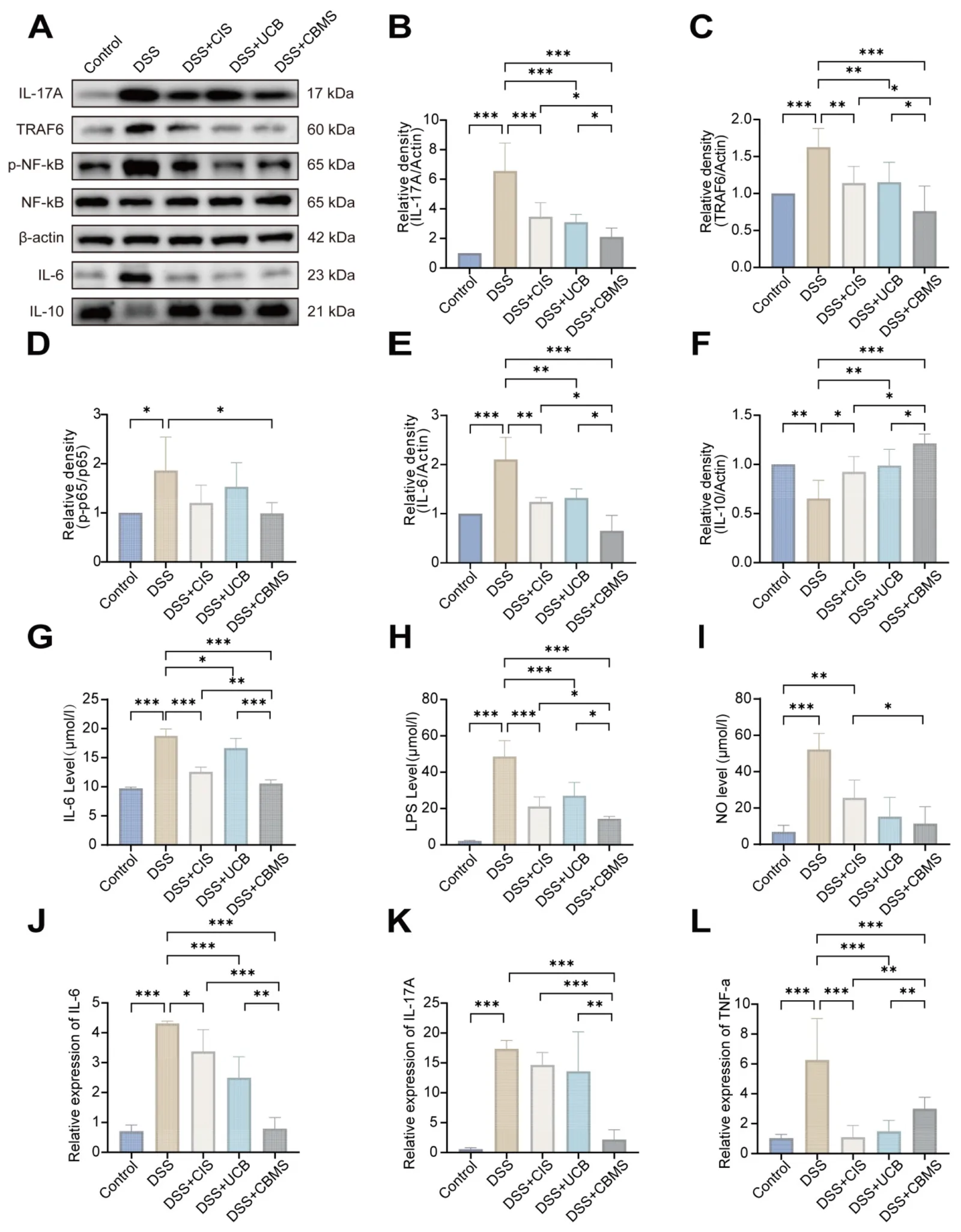
CBMS modulates inflammatory signaling pathways and cytokines in DSS-induced UC mice. (**A**) Protein expression of IL-17, TRAF6, P-NF-κB, NF-κB, IL-6, and IL-10 in intestinal tissues. (**B**–**F**) Relative grayscale values of IL-17, TRAF6, P-NF-κB/NF-κB, IL-6, and IL-10. (**G**) Serum LPS levels by ELISA. (**H**) Serum IL-6 levels by ELISA. (**I**) Serum NO levels by ELISA. (**J**–**L**) mRNA expression of IL-6, IL-17A, and TNF-α. * *p* < 0.05, ** *p* < 0.01, *** *p* < 0.001. Data represent means ± SD. *n* ≥ 3.

Next, we evaluated the immune response and inflammatory status in the colon and systemic circulation of the mice. IL-17 and NF-κB are crucial inflammatory signaling pathways [20]. Receptor proteins in these inflammatory pathways can regulate the transcription and expression of genes by binding to promoter and enhancer sequences of various pro-inflammatory genes, thereby exacerbating the inflammatory response [21,22]. The NF-κB dimer is sequestered in the cytoplasm of unstimulated cells through interaction with its inhibitor IκB. Upon LPS stimulation, IκB is phosphorylated, leading to the release of NF-κB. Phosphorylated NF-κB translocates to the nucleus and activates cytokine expression [22]. Western blot results revealed that key molecules of the IL-17-TRAF6-NF-κB axis (IL-17, TRAF6, p-NF-κB, and NF-κB) were significantly upregulated in the colons of DSS-treated mice, confirming the activation of this pathway. CBMS treatment suppressed this pathway to some extent, and its inhibitory effect was significantly superior to that of UCB and CIS (Figure 5A–D). Further analysis of inflammation markers in both the colon and serum showed that in the DSS group, the expression levels of the pro-inflammatory cytokine IL-6 and the anti-inflammatory cytokine IL-10 were significantly reduced in the colon. CBMS treatment reversed this trend, with a significantly greater effect than that of UCB and CIS (Figure 5E,F). ELISA results showed that levels of LPS, IL-6, and NO were significantly elevated in the serum of the DSS group, whereas CBMS treatment significantly reduced these levels, with CBMS outperforming UCB and CIS in its therapeutic effects (Figure 5G–I). RT-qPCR results demonstrated that the expression levels of pro-inflammatory cytokines IL-6, IL-17A, and TNF-α were significantly increased in the colon of DSS-treated mice. CBMS treatment significantly reduced these levels, and its therapeutic efficacy was significantly superior to that of UCB and CIS (Figure 5J–L).

These results indicate that CBMS significantly inhibits both intestinal and systemic immune responses and inflammation. By modulating the balance of pro-inflammatory and anti-inflammatory cytokines, CBMS effectively restores intestinal immune homeostasis.

### 3.6. CBMS Alleviates Dysbiosis in DSS-Induced UC Mice

**Figure 6:**
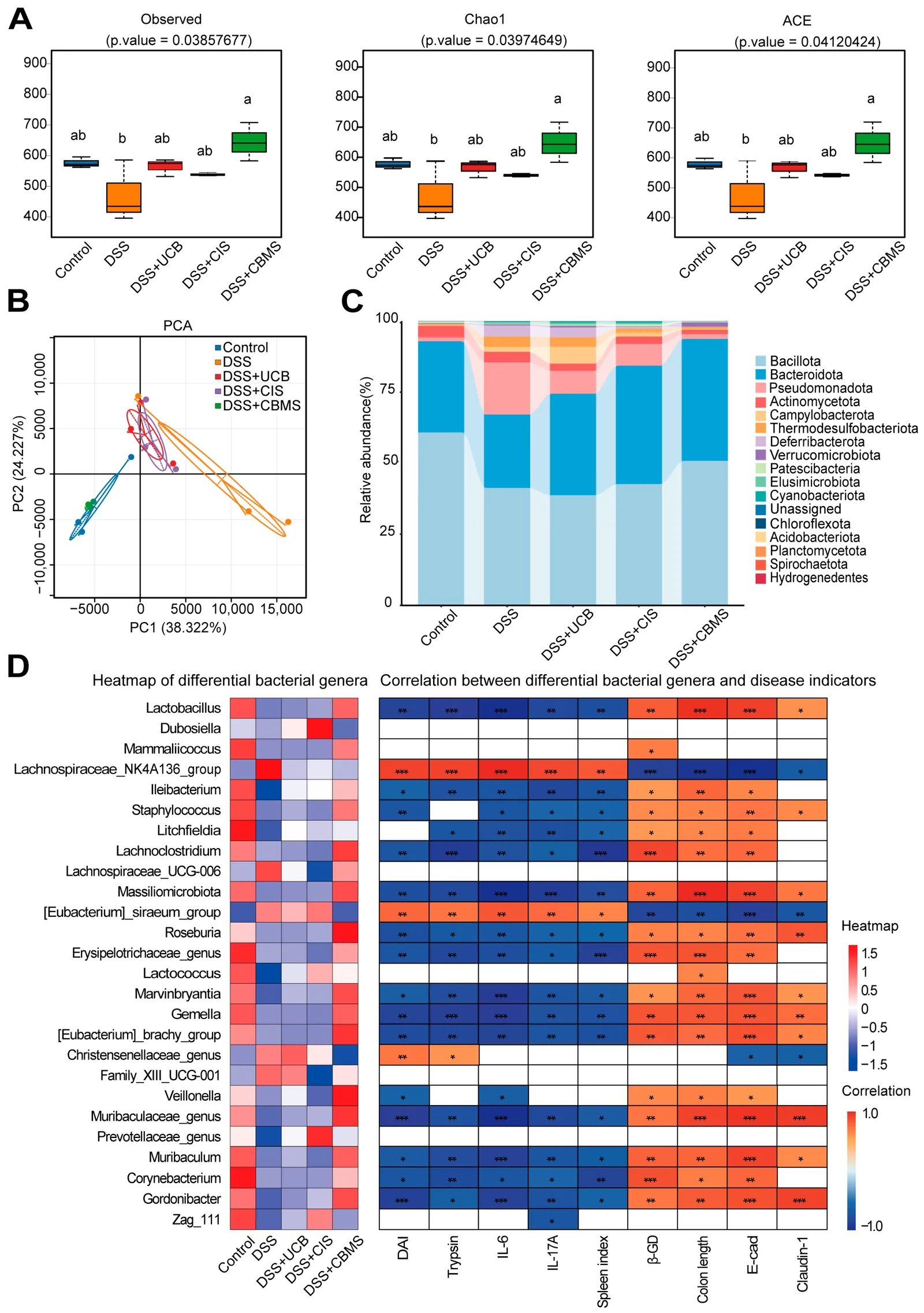
CBMS improves DSS-induced gut microbiota dysbiosis. (**A**) Alpha diversity of the gut microbiota between the four groups. (**B**) Beta diversity of the gut microbiota between the five groups was assessed by principal coordinate analysis using the Bray–Curtis index. (**C**) The relative abundance of bacteria at the phylum level between the five groups is presented by the colorful columns. (**D**) Heatmap showing the inter-group changes in the correlation between the differential bacterium genera and disease indicators on the left; on the right, the heatmap of the correlation between the differential genera. Different letters (a and b) indicate statistically significant differences among groups in diversity analyses (*p* < 0.05). Asterisks indicate the statistical significance of correlations: * *p* < 0.05, ** *p* < 0.01, and *** *p* < 0.001.

In ulcerative colitis (UC), damage to the intestinal mucosal barrier and the activation of intestinal and systemic immune responses are closely associated with gut microbiota. To investigate the effect of CBMS on the gut microbiota in DSS-induced colitis mice, we performed in-depth analysis of the microbiome composition and structure using 16S rRNA gene sequencing. Alpha diversity analysis showed that the observed species, Chao1 index, and ACE index were significantly reduced in the DSS group, indicating a decrease in species richness. In contrast, CBMS treatment significantly restored these indices, with superior recovery compared to UCB and CIS (Figure 6A). Principal Component Analysis (PCA) revealed significant separation in the microbial community structure among the five groups: the Control and CBMS groups clustered together, while the UCB and CIS groups clustered separately, both distinctly separated from the DSS group. This indicates that CBMS more effectively restored the microbial structure to a more normal state (Figure 6B).

At the phylum level, compared to the Control group, DSS-induced dysbiosis in the gut microbiota was evident, with significant increases in the relative abundance of *Bacteroidota*, *Pseudomonadota*, *Campylobacterota*, *Thermodesulfobacteriota*, *Deferribacterota*, *Verrucomicrobiota*, *Elusimicrobiota*, *Cyanobacteria*, and *Chloroflexota*. Conversely, the abundance of *Bacillota*, *Actinomycetota*, *Patescibacteria*, *Acidobacteriota*, *Planctomycetota*, *Spirochaetota*, and *Hydrogenedentes* was significantly reduced. CBMS treatment markedly reversed this dysbiosis: the phyla that were abnormally elevated in the DSS group were suppressed in the CBMS, returning to levels close to or lower than those of the Control group. Additionally, phyla that were reduced (e.g., *Bacillota*, *Actinomycetota*) showed significant recovery, with some even surpassing Control group levels. In contrast, UCB and CIS had weaker regulatory effects: UCB was unable to effectively restore beneficial microbiota such as *Bacillota* and *Actinomycetota*, while CIS only suppressed a few phyla (e.g., *Pseudomonadota*, *Campylobacterota*), with overall recovery less pronounced than CBMS (Figure 6C). At the genus level, compared to the Control group, the DSS group showed significant changes in nine genera, including *Lactobacillus*, *Lachnospiraceae_NK4A136_group*, *Leibacterium*, *Eubacterium_siraeum_group*, *Lactococcus*, *Gemella*, *Muribaculaceae_genus*, and *Zag_111*, among others. Of these, *Lachnospiraceae_NK4A136_group* was restored in all three treatment groups. However, *Lactobacillus*, *Leibacterium*, *Eubacterium_siraeum_group*, *Lactococcus*, *Gemella*, and *Muribaculaceae_genus* were significantly restored only in the CBMS group. Additionally, genera such as *Mammaliicoccus*, *Staphylococcus*, *Litchfieldia*, *Lachnoclostridium*, *Massilia*, *Roseburia*, *Erysipelotrichaceae_genus*, [*Eubacterium*]*_brachy_group*, *Christensenellaceae_genus*, *Veillonella*, *Prevotellaceae_genus*, *Muribaculum*, *Corynebacterium*, and *Gordonibacter* also showed significant changes exclusively in the CBMS (Figure 6D). Pearson correlation analysis revealed that, among 26 differential genera, 23 were associated with disease indicators, and 22 were linked to CBMS treatment efficacy. Most genera (e.g., *Lactobacillus*, *Lachnoclostridium*, *Litchfieldia*, *Muribaculaceae_genus*, *Muribaculum*) showed significant negative correlations with inflammation indicators (DAI, Trypsin, IL-6, IL-17A, spleen index), and positive correlations with barrier and tissue protection markers (β-GD, colon length, E-cadherin, Claudin-1).

In summary, CBMS performs better than UCB and CIS in correcting DSS-induced gut microbiota dysbiosis, thereby aiding in the restoration of intestinal microbial balance.

### 3.7. CBMS Promotes the Formation of a New Balance in the Gut Microbiota of DSS-Induced UC Mice

**Figure 7:**
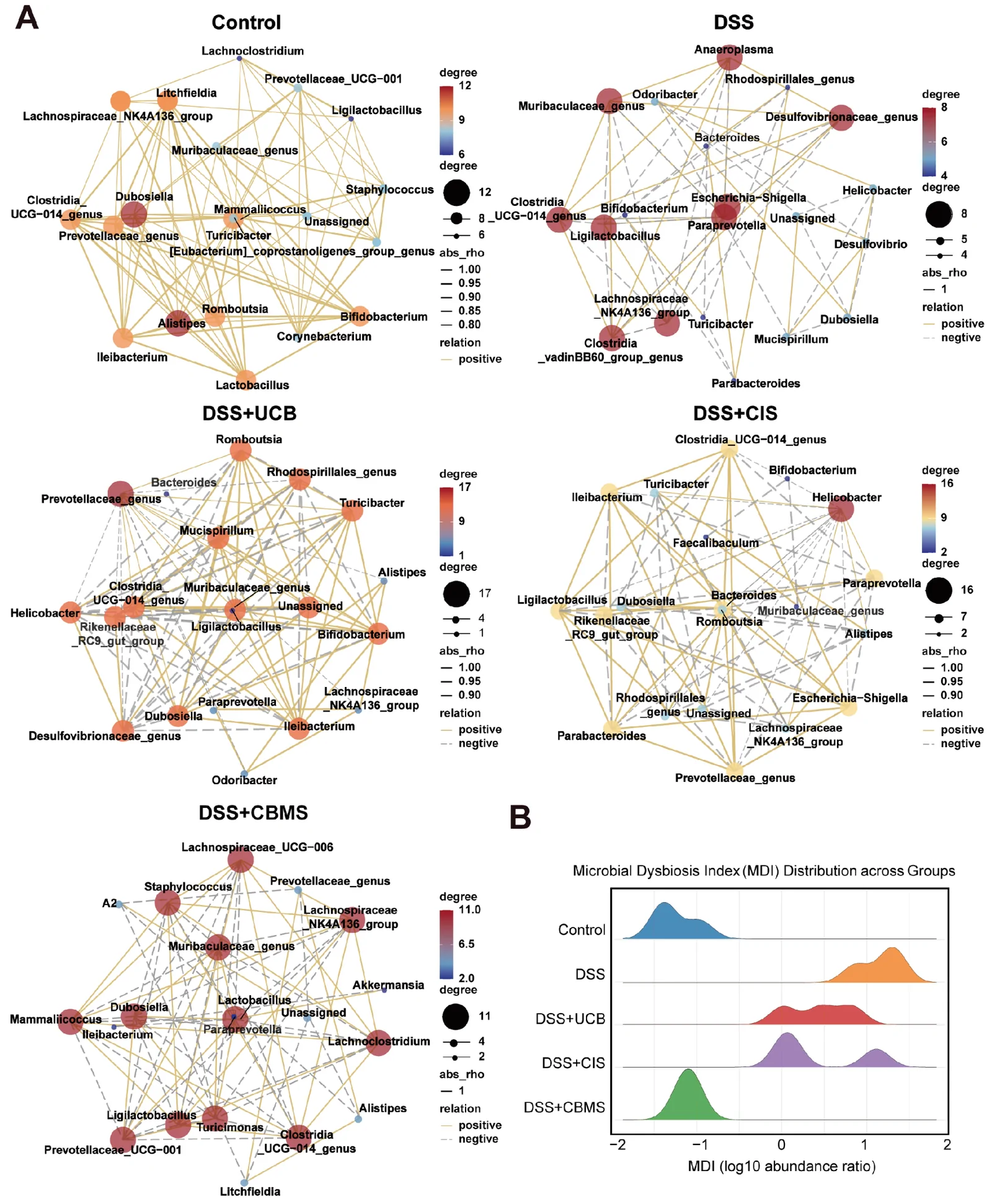
CBMS promotes the establishment of a new balance in the gut microbiota of DSS-induced UC mice. (**A**) Co-occurrence network analysis of gut microbiota in five groups of mice. (**B**) Microbial Dysbiosis Index (MDI) of the five groups of mice.

To investigate the bacterial co-occurrence network and homeostasis of the gut microbiota after CBMS intervention in DSS-induced UC mice, we constructed a bacterial co-occurrence network at the genus level using Spearman correlation analysis and analyzed the co-occurrence networks across five groups of mice (Figure 7A). The results showed that the network correlation and scale of the gut microbiota in the DSS group were significantly reduced. However, after CBMS intervention, both the network correlation and scale of the gut microbiota were restored, although the composition of the network genera changed. This suggests that CBMS intervention led to the formation of a new homeostasis in the gut microbiota of the mice. Furthermore, we calculated the Microbial Dysbiosis Index (MDI). The results indicated that the MDI in the Control group was concentrated between −2 and −1, with a significant peak, indicating stable microbiota structure and low dysbiosis. In contrast, the MDI in the DSS group shifted significantly to the right, ranging from 1 to 2, and was completely separated from the Control group, indicating significant dysbiosis following DSS induction. In the treatment groups, the MDI in the CIS and UCB was distributed between 0 and 1, showing bimodal or multimodal characteristics, suggesting that single treatments partially alleviated dysbiosis but did not fully restore microbial balance. In contrast, the MDI in the CBMS closely overlapped with the Control group, concentrated between −2 and −1, indicating that CBMS treatment significantly reversed DSS-induced dysbiosis and restored the gut microbiota to a state close to health (Figure 7B). These results confirm that CBMS intervention not only restored the overall stability of the gut microbiota co-occurrence network but also altered the genera composition within the network. MDI analysis further substantiated that CBMS could significantly reverse dysbiosis and restore the microbial distribution to a healthy range.

### 3.8. CBMS Restores Gut Homeostasis by Modulating Gut Microbial Function and Immune Response

**Figure 8:**
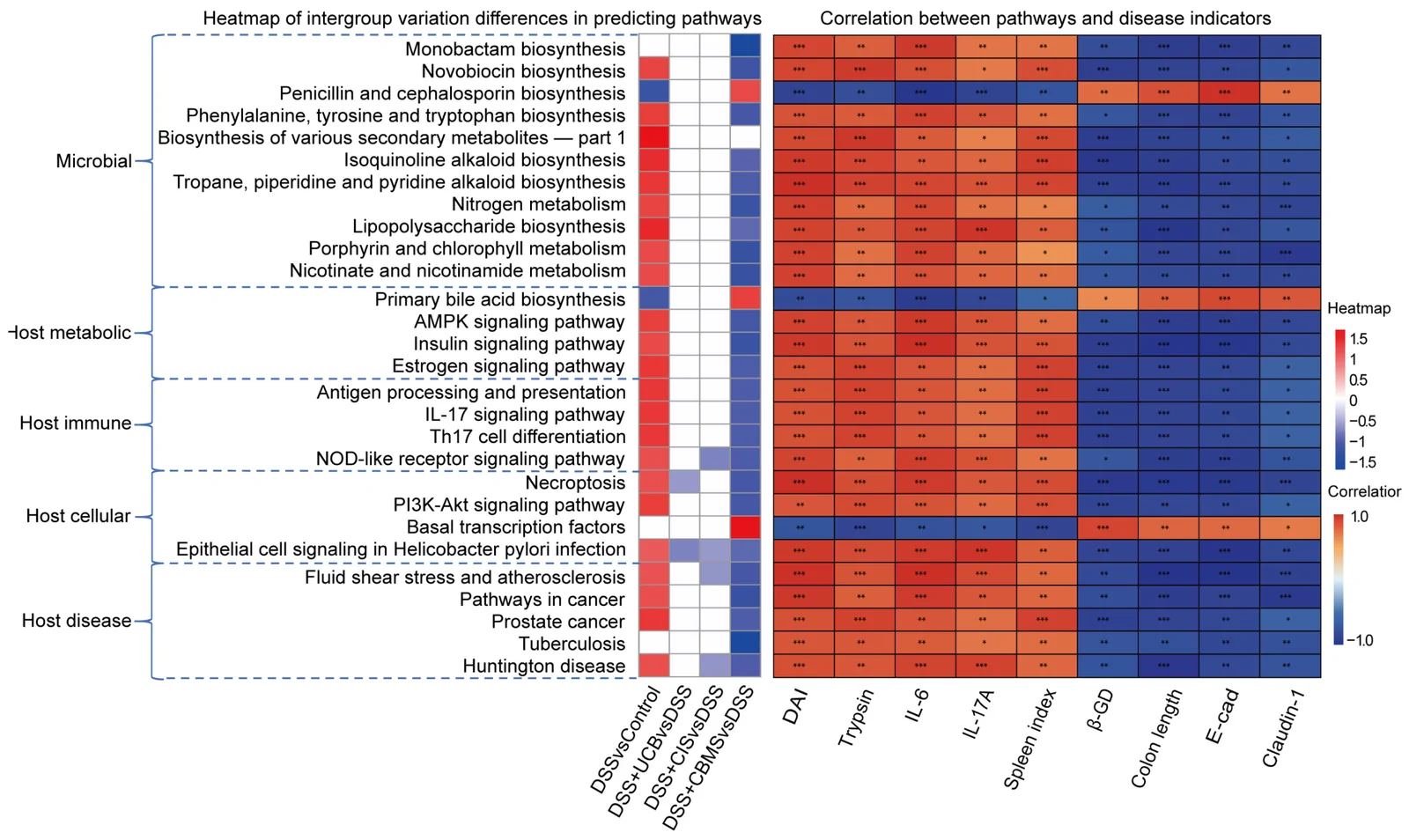
The impact of gut microbiota modulation on predicted functions based on PICRUSt2 analysis of 16S rRNA sequencing data. The heatmap on the left shows the inter-group variation in the correlation between different pathways and disease indicators; on the right, the heatmap illustrates the correlation between pathways and disease indicators. Asterisks indicate the statistical significance of correlations: * *p* < 0.05, ** *p* < 0.01, and *** *p* < 0.001.

Functional prediction analysis based on PICRUSt2 identified 75 pathways with significant inter-group differences. These pathways were further correlated with disease markers using Spearman correlation analysis, revealing 29 pathways associated with disease markers and 28 pathways significantly correlated with CBMS intervention (Figure 8). Overall, the results indicate significant changes in several microbial functional pathways. Compared to the Control group, multiple microbial metabolic pathways in the Disease group showed notable alterations, primarily involving processes such as antibiotic biosynthesis, amino acid metabolism, nitrogen metabolism, lipopolysaccharide synthesis, and cofactor and vitamin metabolism. Generally, the microbial metabolic potential was enhanced in the disease state, while CBMS treatment significantly reversed these pathway changes.

Specifically, pathways related to the biosynthesis of phenylalanine, tyrosine, and tryptophan, secondary metabolites biosynthesis (part 1), biosynthesis of isoquinoline alkaloids, tropane alkaloids, and pyridine alkaloids, nitrogen metabolism, lipopolysaccharide biosynthesis, and porphyrin and chlorophyll metabolism, as well as niacin and nicotinamide metabolism, were significantly upregulated in the Disease group but downregulated in the CBMS treatment group. This suggests that CBMS effectively suppresses microbial functional abnormalities associated with inflammation, endotoxin production, and energy metabolism reprogramming.

In addition to microbial metabolic pathways, functional prediction also identified several pathways related to host metabolism, immune response, and disease, including the IL-17 signaling pathway, Th17 cell differentiation, and necroptosis. It is important to note that PICRUSt2 uses 16S rRNA sequencing data to infer microbial function, and the predicted results primarily reflect the potential metabolic capabilities of the gut microbiota and its inflammatory-related functional features. These results do not directly represent real changes in host immune pathways or signal transduction. Therefore, changes in host immune and disease-related pathways across different treatment groups can only be considered as indirect clues to understanding “microbiota–host interactions,” providing directional reference for subsequent mechanistic studies.

In previous experiments, we assessed the changes in lipopolysaccharide (LPS) and the IL-17 signaling pathway (Figure 5). The results showed that the trends in LPS and IL-17 changes in the Control, Disease, and CBMS treatment groups were consistent with the pathway predictions. Both LPS and IL-17 were significantly upregulated in the Disease group and markedly downregulated after CBMS treatment. This suggests that CBMS may modulate specific immune pathways or associated bacterial populations, thereby regulating gut immune and inflammatory responses. Overall, these findings demonstrate that CBMS effectively restores gut immune homeostasis, suppresses excessive inflammation, and aligns with its mechanism of modulating gut microbiota functionality.

## 4. Discussion

UCB is the primary form of bilirubin in the body and has demonstrated therapeutic effects in various disease animal models, such as cancer, ischemia–reperfusion injury, and IBD [23]. However, its poor water solubility and susceptibility to oxidation limit its clinical application. CIS, with its excellent biocompatibility and abundant active sites, has been widely utilized in drug delivery systems. In this study, we successfully developed a sustained-release system (CBMS) and demonstrated its significant therapeutic efficacy in a DSS-induced UC model. The DSS-induced acute colitis model in mice is a widely used model that mimics the clinical and histological characteristics of human ulcerative colitis, including intestinal inflammation, epithelial barrier dysfunction, activation of innate immunity, dysbiosis of the gut microbiota, severe bleeding, and eventual mortality [24]. CBMS, by continuously releasing UCB within the intestinal lumen, effectively inactivates overactive digestive proteases, thereby synergistically remodeling the gut microbiota, suppressing excessive immune responses, and repairing intestinal barrier function, providing a sustained and effective solution for UCB-based treatment of IBD.

In IBD, elevated fecal proteases primarily originate from the host [25,26], a finding consistent with the notable changes in pancreatic proteases such as trypsin and chymotrypsin detected in the feces of patients [27,28,29]. Our results confirmed this observation, showing a significant increase in trypsin and chymotrypsin activity in the DSS group (Figure 7). However, CBMS intervention significantly improved these changes, markedly reducing the activity of digestive proteases, indicating that CBMS effectively suppresses overactive digestive proteases and helps protect the intestinal barrier.

The intestinal mucosa serves as a physical barrier that prevents microorganisms and harmful substances from entering the bloodstream. Under normal conditions, protease activity exists in the intestinal mucosa, participating in mucus viscosity regulation and antigen processing. However, exposure to high concentrations of proteases leads to the degradation of intestinal barrier proteins, damaging the mucosal barrier function and triggering bacterial translocation [30]. We assessed the expression of tight junction proteins in intestinal tissue and found that in the DSS group, the expression of tight junction proteins, including Claudin-1, ZO-1, and E-cadherin, was significantly reduced. In the CBMS treatment, the expression of these tight junction proteins was significantly restored (Figure 8), suggesting that CBMS can effectively protect the intestinal barrier. The Wnt/β-catenin signaling pathway plays a critical role in maintaining intestinal stem cell function, promoting epithelial proliferation, and facilitating tissue repair after injury. However, aberrant and sustained activation of this pathway is closely associated with colorectal carcinogenesis [31]. In the present study, DSS-induced inflammatory injury suppressed Wnt/β-catenin signaling activity, whereas CBMS treatment restored pathway activity and promoted epithelial barrier repair. These findings suggest that the effect of CBMS represents a functional restoration of impaired Wnt/β-catenin signaling following inflammatory injury rather than the induction of pathological pathway hyperactivation.

In addition to producing proteases, the gut microbiota also secretes protease inhibitors and protease-degrading enzymes, which contribute to maintaining proteolytic balance. β-GD, a key enzyme secreted by gut bacteria, can convert conjugated bilirubin into UCB [19]. UCB can directly inactivate digestive proteases, thereby reducing protease-mediated intestinal barrier damage. In the DSS-induced colitis model, β-GD activity was significantly decreased, whereas CBMS treatment markedly restored β-GD activity. Moreover, PICRUSt2-based functional prediction indicated that the recovery of β-GD-related microbial functions following CBMS treatment was associated with gut microbiota remodeling (Figure S5). These findings suggest that CBMS preserves intestinal barrier integrity through dual mechanisms: enhancing β-GD activity by improving the intestinal microenvironment and restoring β-GD-producing microbial communities, while UCB released from CBMS directly inhibits digestive proteases, including trypsin and chymotrypsin. Collectively, CBMS-mediated regulation of microbial enzymatic functions, together with the protease-inhibitory activity of UCB, contributes to the alleviation of DSS-induced UC.

Although UCB alone can also exert similar effects and significantly improve colitis, its effect is not as pronounced as that of CBMS. Notably, when CIS was used alone, no significant differences in digestive protease activity or β-GD activity were observed compared to the DSS group. This may be due to the degradation of CIS in vivo by microbial enzymes, lysozyme, and chitinase [32]. Lysozyme is present in animal mucosal secretions, while chitinase is found in gastric juices. Nevertheless, significant recovery of intestinal tight junction proteins was observed in the CIS. Other studies have also confirmed that CIS can promote the expression of tight junction proteins, such as Claudin-1, Occludin, and ZO-1 [33]. This suggests that CIS may exert its effects through other mechanisms, potentially by maintaining a certain structure in the gut due to the higher viscosity of CIS solutions, which physically contacts the intestinal mucosa, thereby providing a protective effect. Additionally, digestive proteases may be diluted and their concentrations reduced, mitigating their damaging effects on the mucosal barrier.

The gut microbiota plays a critical role in the pathogenesis of IBD. IBD patients often exhibit dysbiosis, including reduced microbial diversity, changes in microbial composition and function, reduced beneficial bacteria, increased harmful bacteria, and a predominance of pro-inflammatory over anti-inflammatory bacteria. This dysbiosis disrupts normal intestinal function and triggers excessive immune responses [34]. Our previous studies have confirmed significant structural changes in the gut microbiota at the phylum level in the DSS-induced UC mouse model [35]. In this study, we reached similar conclusions. Compared to the Control group, the DSS group showed significant reductions in α-diversity and β-diversity (Figure 5), indicating marked changes in the microbial composition and dysbiosis. After CBMS treatment, both α-diversity and β-diversity showed an upward trend, suggesting that CBMS can improve dysbiosis. Further analysis at the genus level revealed that CBMS treatment restored the gut microbiota to a new homeostasis, as evidenced by the recovery of microbial network correlation and scale. CBMS significantly suppressed harmful phyla such as *Pseudomonadota*, *Campylobacterota*, and *Thermodesulfobacteriota*, while significantly restoring beneficial phyla like *Bacteroidota* and *Verrucomicrobiota*. Specifically, *Pseudomonadota* contains several potentially pathogenic bacteria, such as *Pseudomonas*, which can cause infections in immunocompromised hosts [36]; *Campylobacterota* is associated with intestinal infections and diarrhea [37]; and *Thermodesulfobacteriota* includes some thermophilic sulfate-reducing bacteria, which may cause health issues when they overgrow in the gut [38]. *Bacteroidota* is typically considered a hallmark of gut health, helping in fiber degradation, short-chain fatty acid production, immune regulation, and maintaining intestinal barrier function [39], while *Verrucomicrobiota* is considered beneficial, participating in glucose homeostasis, exhibiting anti-inflammatory properties, and supporting the maintenance of the intestinal barrier, with its abundance being inversely related to diseases like obesity and diabetes [40].

At the genus level, CBMS significantly restored several beneficial genera, including *Lactobacillus*, widely used as a probiotic to help maintain gut health and regulate the microbiota [41]; *Eubacterium_siraeum_group*, involved in short-chain fatty acid production, which is generally considered beneficial for gut health [42]; *Lactococcus*, a member of lactic acid bacteria that helps maintain intestinal microbiota balance [43,44]; *Roseburia*, known for producing short-chain fatty acids with anti-inflammatory effects that protect the intestinal barrier [45]; *Christensenellaceae_genus*, involved in fiber fermentation and inversely related to inflammation and metabolic disorders [46]; *Muribaculum*, a commensal bacterium that is a predominant species in healthy mice [47]; and *Lachnoclostridium*, which generates short-chain fatty acids to protect the intestinal barrier and may help suppress colonic inflammation [48]. The *Eubacterium brachy group* is reported to be negatively correlated with DSS-induced colitis but positively correlated with the Control group [49]. However, CBMS also indirectly regulated several potentially harmful bacteria, including Staphylococcus, some species of which, like *Staphylococcus* aureus, are known pathogens that can cause intestinal infections or inflammation [50]; *Erysipelotrichaceae_genus*, whose species may be linked to gut diseases and inflammation [51]; and *Corynebacterium*, which is associated with intestinal inflammation and immune responses, showing potential harmful effects [52]. This suggests that the therapeutic effect of CBMS primarily operates by increasing the abundance of beneficial bacteria and indirectly regulating harmful bacteria.

Functional prediction analysis based on PICRUSt2 showed significant changes in several microbial metabolic pathways in the DSS group compared to the Control group, involving processes such as antibiotic biosynthesis, amino acid metabolism, nitrogen metabolism, lipopolysaccharide synthesis, and vitamin metabolism, with most pathways showing enhanced trends in the disease state. CBMS treatment significantly reversed these changes, especially in pathways related to inflammation, endotoxin generation, and energy metabolism reprogramming, suggesting that CBMS regulates microbial community functions to suppress metabolic abnormalities. In particular, pathways related to the biosynthesis of phenylalanine, tyrosine, and tryptophan, as well as secondary metabolites, were significantly upregulated in the DSS group but downregulated after CBMS treatment. However, some pathways, such as secondary metabolite biosynthesis, showed elevated levels in the DSS group, with no significant changes after CBMS treatment, suggesting that these may be associated with microbial dysfunction at specific disease stages and are less responsive to CBMS intervention. Antibiotic-related pathways exhibited different response patterns, with CBMS modulating antibiotic metabolism-related pathways to restore microbial homeostasis. Furthermore, no significant changes were observed in the UCB and CIS treatment compared to the DSS group, further highlighting the unique advantages of CBMS in regulating gut microbial metabolic functions.

Functional prediction analysis also identified several pathways associated with host immunity and metabolism, including the IL-17 signaling pathway, Th17 cell differentiation, and necroptosis. While the predicted results reflect the potential metabolic capacity of the gut microbiota, they do not directly represent the true changes in host immune responses. Experimental validation of the LPS and IL-17 pathways was consistent with the functional prediction analysis, further confirming the regulatory effect of CBMS on inflammation. Overall, CBMS treatment modulates the metabolic functions of the gut microbiota, thereby indirectly influencing the host’s inflammatory and immune responses, while UCB and CIS effects are more dependent on direct immune modulation. The PICRUSt2 analysis revealed the abnormal activation of gut microbiota metabolism in disease states and highlighted the potential of CBMS in reversing these abnormalities, providing important insights for further mechanistic studies.

In summary, our study demonstrates that CBMS protects the mucus layer and underlying intestinal tissues by inactivating digestive proteases. It activates the Wnt/β-catenin signaling pathway, normalizing the structure and function of goblet cells, epithelial cells, and stem cells. Consequently, CBMS enhances tight junction proteins to protect the intestinal mucosal barrier, inhibiting the penetration of bacteria and other toxic substances from the gut lumen. Additionally, CBMS suppresses the IL-7/TRAF6/NF-κB inflammatory signaling pathway, alleviating inflammation, maintaining normal proliferation and differentiation of intestinal epithelial cells, supporting intestinal function, and restoring a balanced gut microbiota composition.

Although this study focused on DSS-induced UC, the modular design of CBMS may provide potential applications for other intestinal inflammatory disorders, including Crohn’s disease. Future optimization of particle properties, release kinetics, and disease-specific targeting strategies will be required to adapt CBMS for different pathological conditions.

## 5. Conclusions

This study developed CBMS which safely delivers UCB while showcasing robust therapeutic efficacy in ulcerative colitis. CBMS integrates symptom alleviation, anti-inflammatory action, barrier restoration, and microbiota modulation into a unified strategy, offering novel insights for UC clinical management. Future research will focus on optimizing fabrication protocols, elucidating mechanistic pathways, and advancing translational applications to bridge preclinical and clinical gaps.

## Data Availability

All data needed to evaluate the conclusions in the paper are present in the paper and/or the Supplementary Materials.

## Supplementary Materials

The following supporting information can be downloaded at https://www.mdpi.com/article/10.3390/biom16081140/s1. Figure S1: Drug Delivery Characteristics of CBMS. (A) Full-wavelength absorption spectrum of UCB solution. (B) Absorbance spectrum of UCB standard solutions at concentrations ranging from 0.1 to 0.6 mg/mL. (C) Standard curve for UCB. (D) Absorbance spectrum of the solution formed after dissolving 3 mg of CBMS.; Figure S2: Detect the cell viability of NCM460 cell at 12, 24, and 48 h using CCK-8 experiment.; Figure S3: Serum biochemical analysis of (A) ALT, (B) AST, (C) BUN, and (D) CREA levels after treatment (*n* = 3).; Figure S4: Safety Profile of CBMS in Mice. HE staining of heart, liver, spleen, lung, and kidney tissues in mice (*n* = 3).; Figure S5: Predicted bacterial genera contributing to GUSB (K01195) abundance based on PICRUSt2 functional analysis.; Table S1: The criteria of DAI; Table S2: The criteria of histology analysis for colonic damage score; Table S3: Primers used for colon tissue RNA qRT-PCR; Table S4: The results of drug release model fitting of CBMS; Material S1: Original images of Western blot analysis in Figure 4 and Figure 5.

## Abbreviations

Abcb1a	ATP-binding cassette, sub-family B member 1A
BAPNA	N-Benzoyl-DL-arginine-4-nitroanilide hydrochloride
BTEE	N-Benzoyl-L-tyrosine ethyl ester
CBMS	Chitosan–bilirubin microspheres
CIS	Chitosan
DAI	Disease activity index
DLS	Dynamic Light Scattering
DMSO	Dimethyl sulfoxide
DSS	Dextran sulfate sodium
FTIR	Fourier transform infrared spectroscopy
IL-10	Interleukin-10
IL-17A	Interleukin-17a
IL-6	Interleukin-6
LPS	Lipopolysaccharide
NF-κB	Nuclear factor kappa-B
NO	Nitrogen monoxide
PCoA	Principal coordinate analysis
p-NF-κB	Phospho-nuclear factor kappa-B
PNPG	4-Nitrophenyl β-D-glucopyranoside
ROS	Reactive oxygen species
SEM	Scanning electron microscopy
TBIL	Total bilirubin
TNF-α	Tumor Necrosis Factor-α
TRAF6	TNF receptor associated factor 6
UC	Ulcerative colitis
UCB	Unconjugated bilirubin
ZO-1	Zonula occludens-1
β-GD	β-glucuronidase
