# Supplementary material for: Chitosan–Unconjugated Bilirubin Microspheres Alleviate Dysbiosis, Immune Dysregulation, and Intestinal Barrier Damage in Ulcerative Colitis
Source: Biomolecules. 2026 Aug 5;16(8):1140. doi: 10.3390/biom16081140 (PMC13511252; doi:10.3390/biom16081140)
Supplement: Supplementary file 1 [file biomolecules-16-01140-s001.zip › biomolecules-4403718-supplementary.pdf]

Chitosan–Unconjugated Bilirubin Microspheres Alleviate Dysbiosis, Immune Dysregulation, and Intestinal Barrier Damage in Ulcerative Colitis

Xinyu Lyu [1,2,†], Xiaotong Xu [1,†], Mengqi Shi [2], Rui Wang [2], Xiaoqing Yu [2], Yan Liu [2], Xiangyu Xue [2], Fengmin Zhang [1,3,*] and Xiuhong Wang [2,4,*]

1 Department of Microbiology, School of Basic Medical Sciences, WU Lien-Teh Institute, Harbin Medical University, Harbin 150081, China; 202501020@hrbmu.edu.cn (X.L.)

2 Department of Biochemistry and Molecular Biology, School of Basic Medical Sciences, Harbin Medical University, Harbin 150081, China

3 Heilongjiang Provincial Key Laboratory of Infection and Immunity, Harbin Medical University, Harbin 150081, China

4 Department of Ultrasound, Harbin Medical University Cancer Hospital, Harbin 150081, China

* Correspondence: fengminzhang@ems.hrbmu.edu.cn (F.Z.); wangxiuhong@hrbmu.edu.cn (X.W.)

† These authors contributed equally to this work.

Table S1. The criteria of DAI

DAI score	Weight loss(％)	Stool consistency	Occult/gross bleeding
0	None	Normal	Normal
1	1-5		
2	5-10	Loose stools	Hemoccult positive
3	10-15		
4	＞15	Diarrhea	Gross bleeding

Table S2. The criteria of histology analysis for colonic damage score

Colon damage score	0	1	2	3
Crypt architecture damage	None	Regeneration	Destruction	
Edema in sub-mucosa	None	Mild	Moderate	Severe
Inflammatory cells infiltration	None /rare	Lamina propria	Sub-mucosa	Muscle layer

Table S3. Primers used for colon tissue RNA qRT-PCR

Target gene	Primer sequence (5’-3’)
Gapdh	F: AGGTCGGTGTGAACGGATTTG
	R: GGGGTCGTTGATGGCAACA
TNF-α	F: CATCTTCTCAAAATTOGAGTGACA
	R: TGGGAGTAGACAAGGTACAACCC
IL-6	F: TACCACTTCACAAGTCGGAGGC
	R: CTGCAAGTGCATCATCGTTGTTC
IL-17A	F: TCAGCGTGTCCAAACACTGAG
	R: CGCCAAGGGAGTTAAAGACTT
IL-10	F: ATAACTGCACCCACTTCCCA
	R: GGGCATCACTTCTACCAGGT

Table S4. The results of drug release model fitting of CBMS

Model	Equation	Correlation coefficient
Ritger-peppas	$Q=13.6t^{0.35}$	R2=0.97158
Higuchi	$Q=7.52t^{1/2}+6.26$	R2=0.94633
First-order	$Q=36.33(1-e^{-0.28t})$	R2=0.90244
Zero-order	$Q=14.99+1.25t$	R2=0.81613

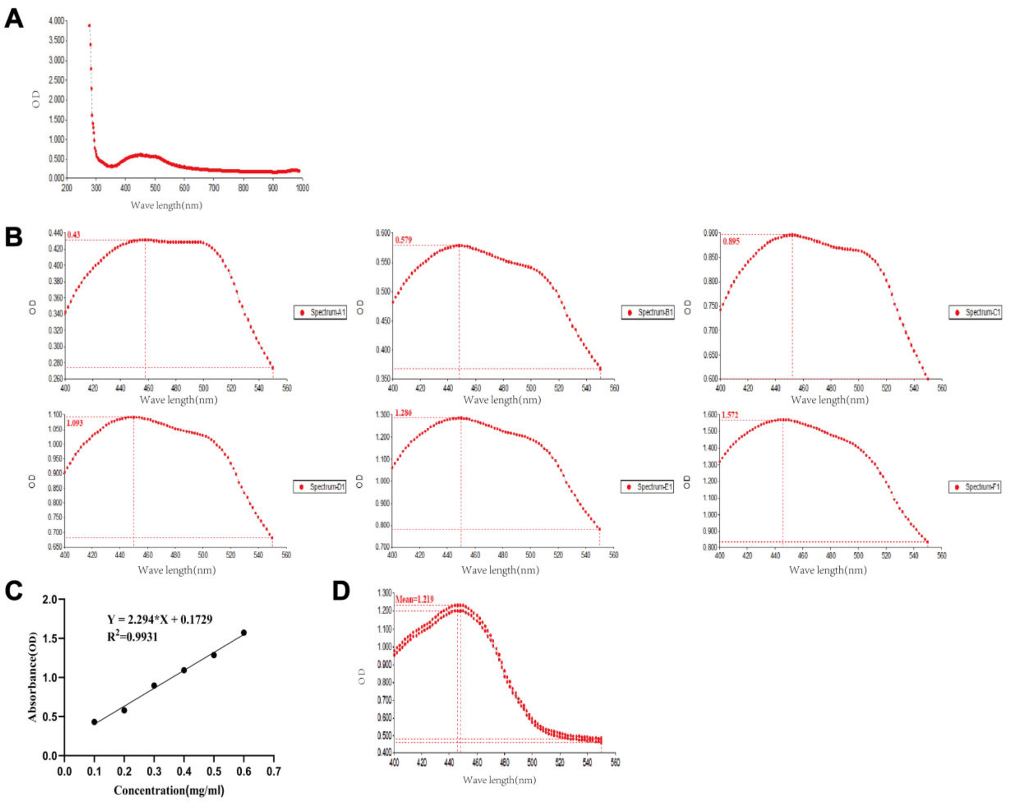

Figure S1. Drug Delivery Characteristics of CBMS. (A) Full-wavelength absorption spectrum of UCB solution. (B) Absorbance spectrum of UCB standard solutions at concentrations ranging from 0.1 to 0.6 mg/ml. (C) Standard curve for UCB. (D) Absorbance spectrum of the solution formed after dissolving 3 mg of CBMS.

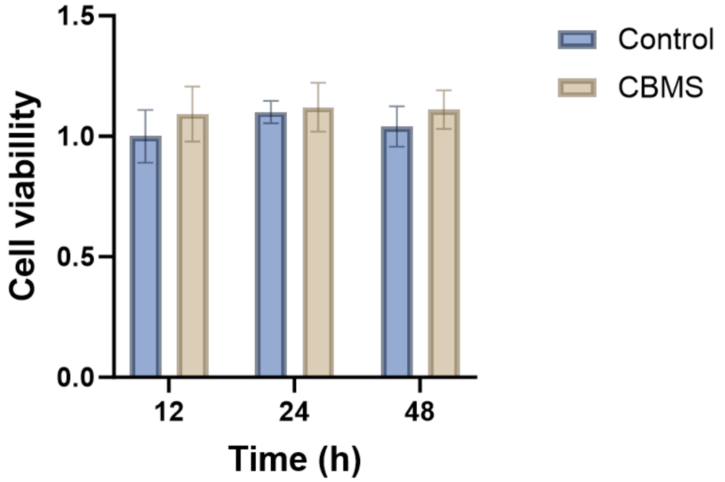

Figure S2. Detect the cell viability of NCM460 cell at 12, 24, and 48 hours using CCK-8 experiment.

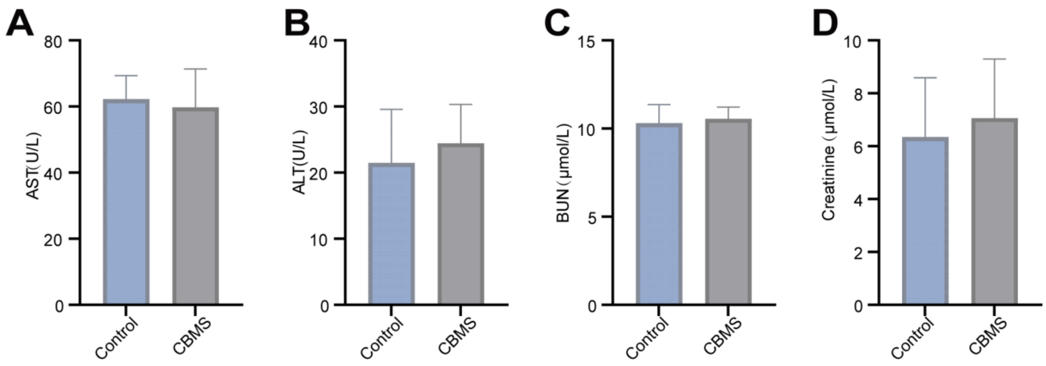

Figure S3. Serum biochemical analysis of (A) ALT, (B) AST, (C) BUN, and (D) CREA levels after treatment (n = 3).

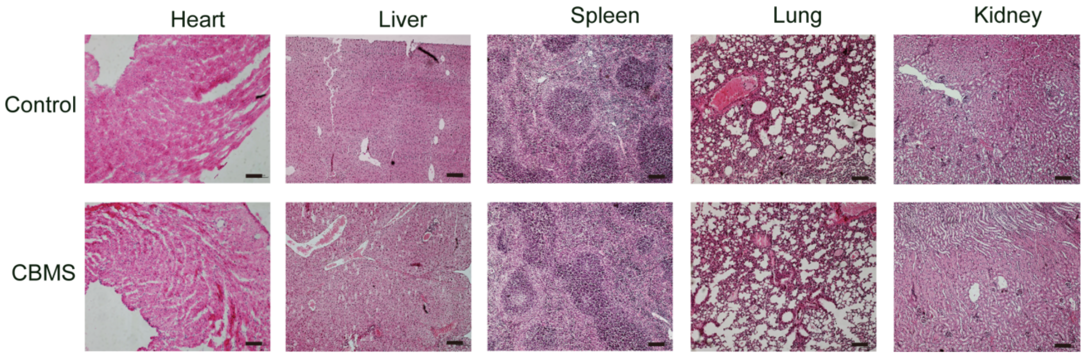

Figure S4. Safety Profile of CBMS in Mice. HE staining of heart, liver, spleen, lung, and kidney tissues in mice (n = 3).

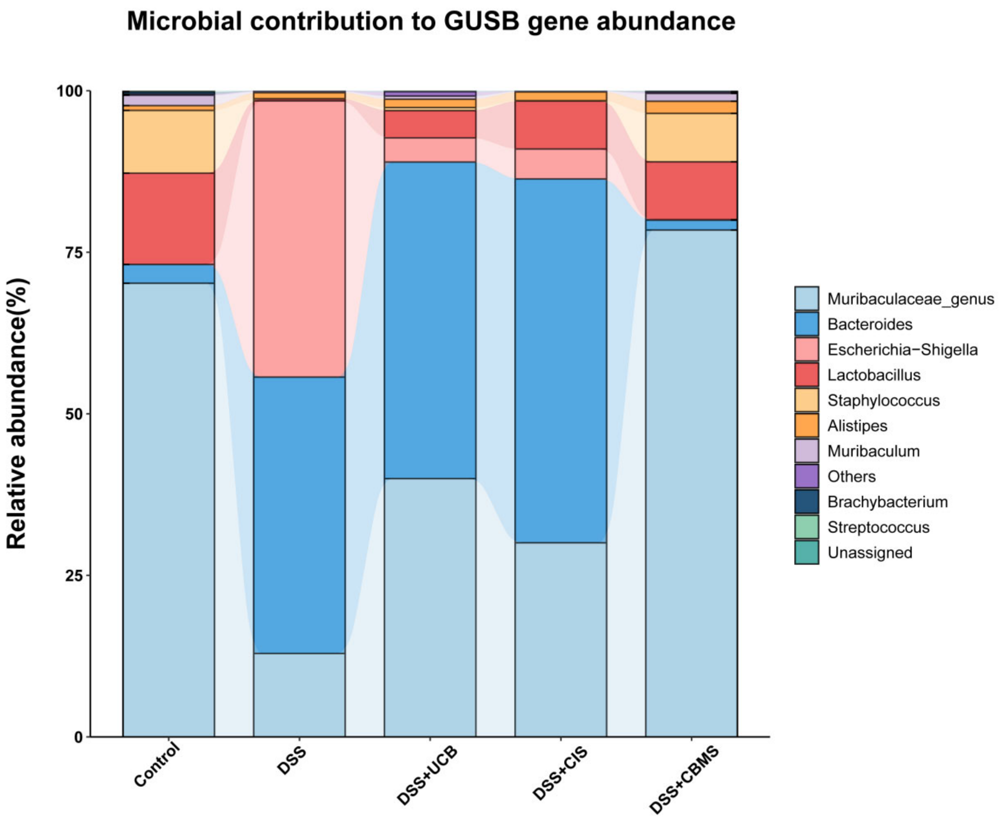

Figure S5. Predicted bacterial genera contributing to GUSB (K01195) abundance based on PICRUSt2 functional analysis.
