# Supplementary material for: Chitosan–Unconjugated Bilirubin Microspheres Alleviate Dysbiosis, Immune Dysregulation, and Intestinal Barrier Damage in Ulcerative Colitis
Source: Biomolecules. 2026 Aug 5;16(8):1140. doi: 10.3390/biom16081140 (PMC13511252; doi:10.3390/biom16081140)

Uncropped gels for Westem Blots in Figure 4 of E-cadherin

E-cadherin

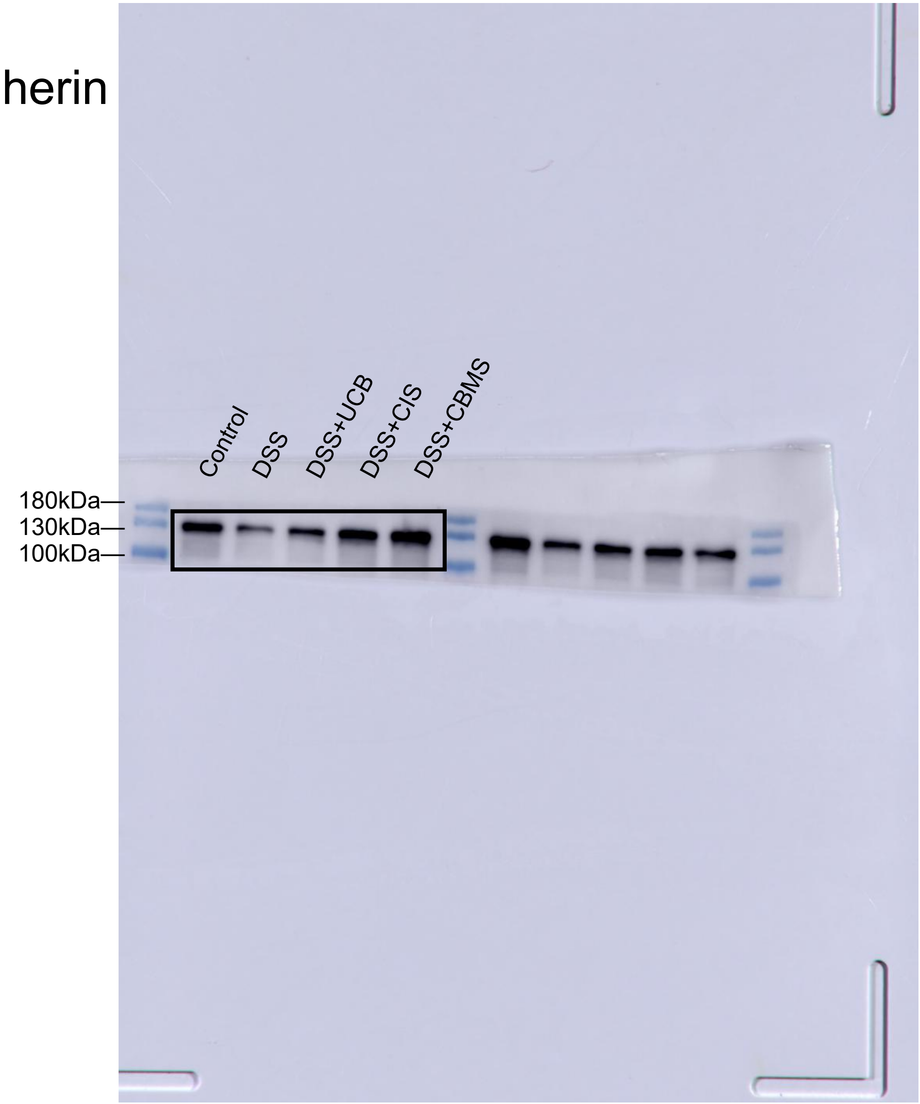

β-actin

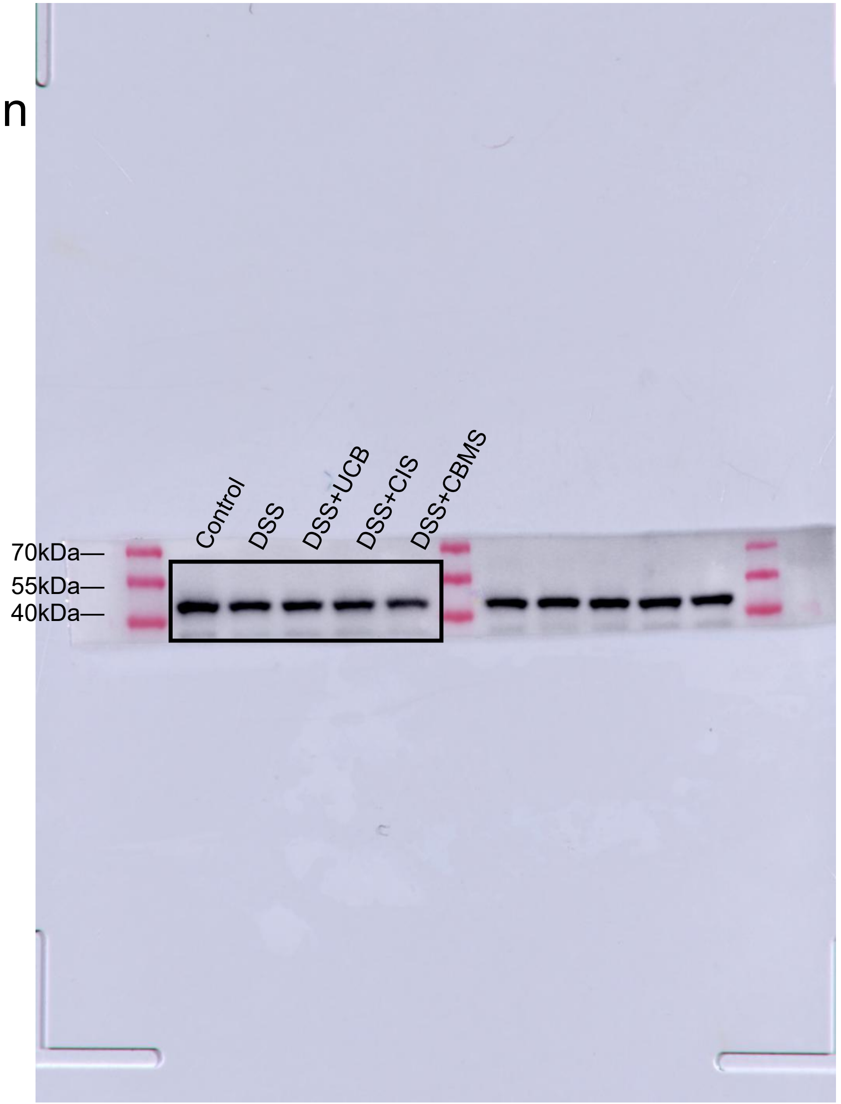

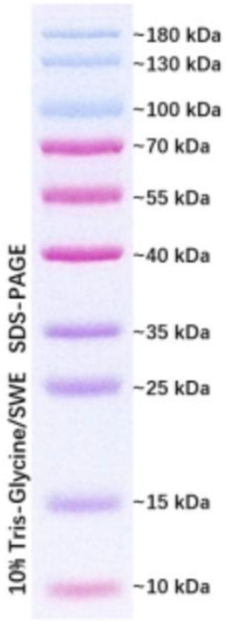

Uncropped gel from repeated Western blot analysis of E-cadherin

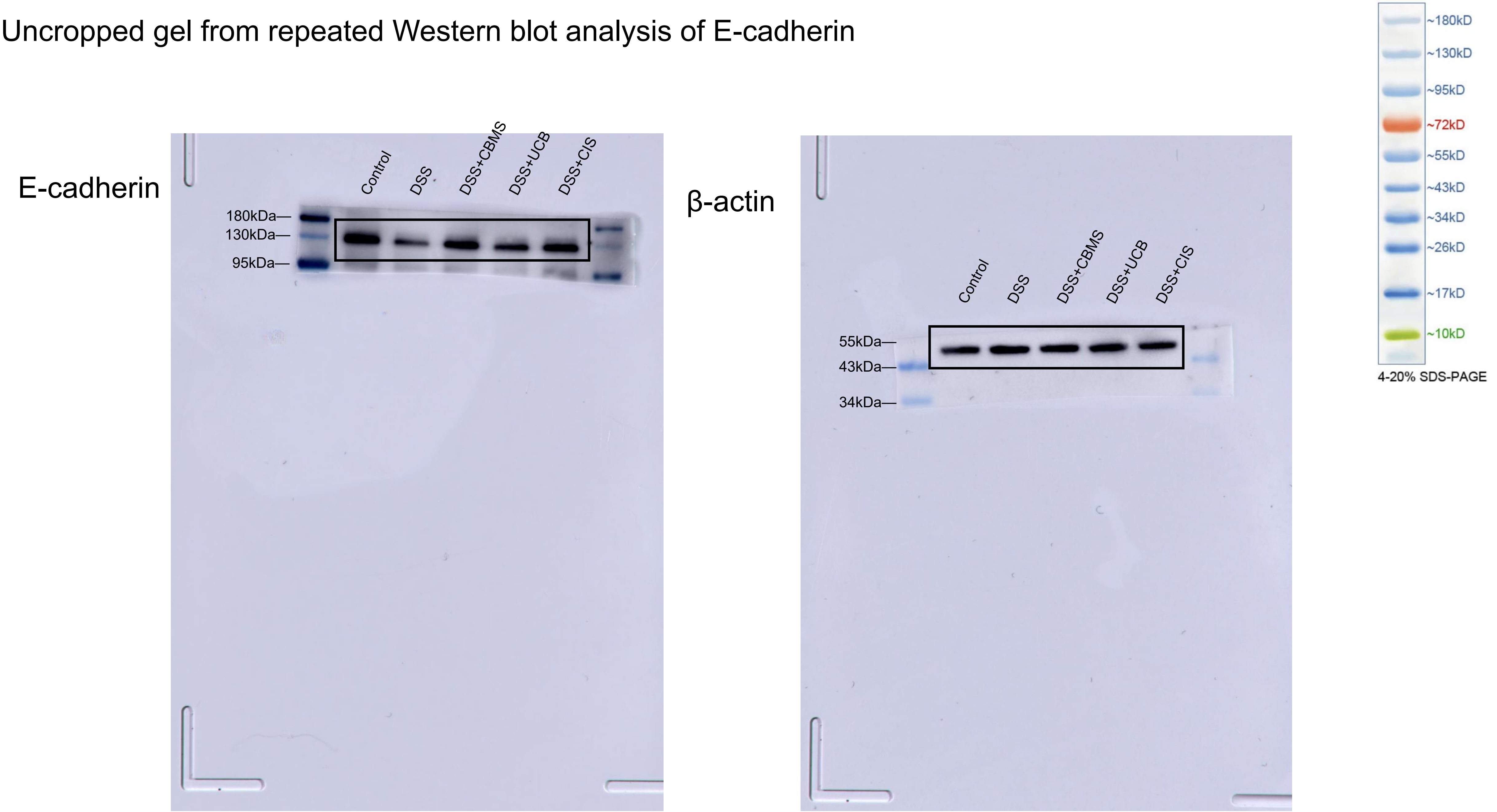

Uncropped gel from repeated Western blot analysis of E-cadherin

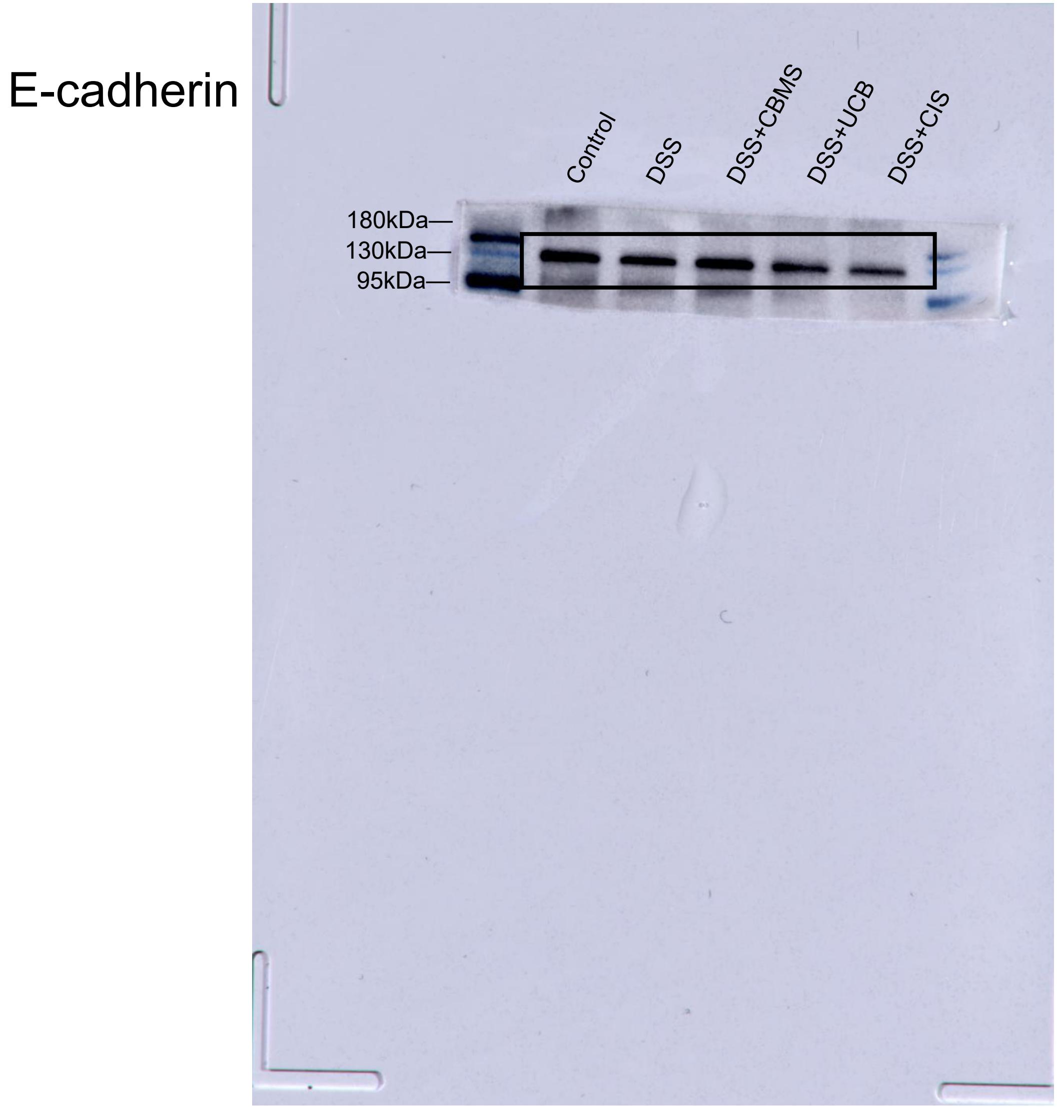

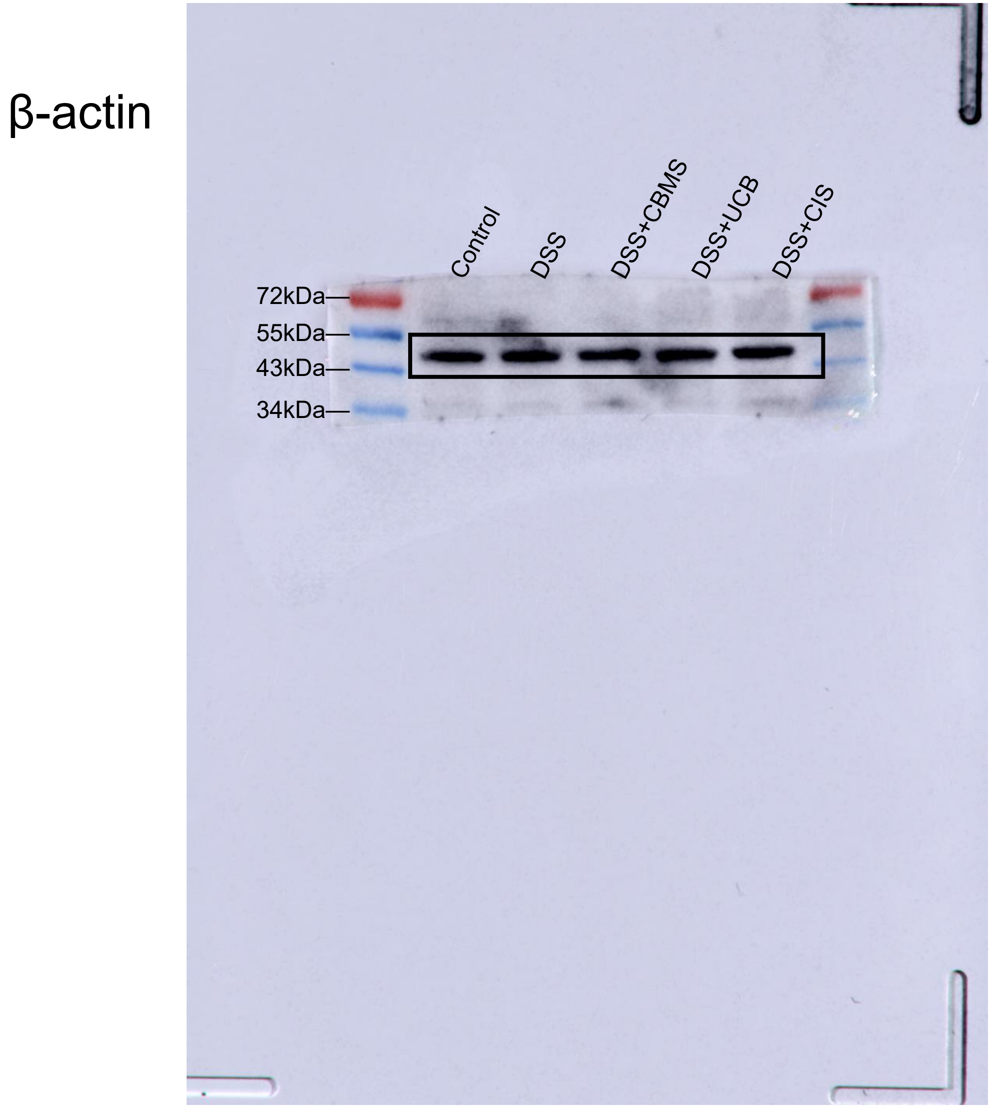

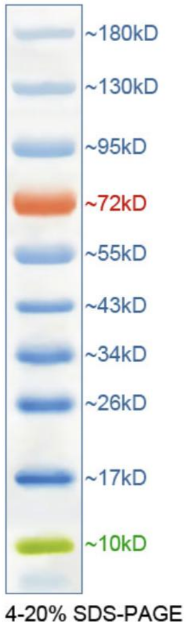

Uncropped gel from repeated Western blot analysis of E-cadherin

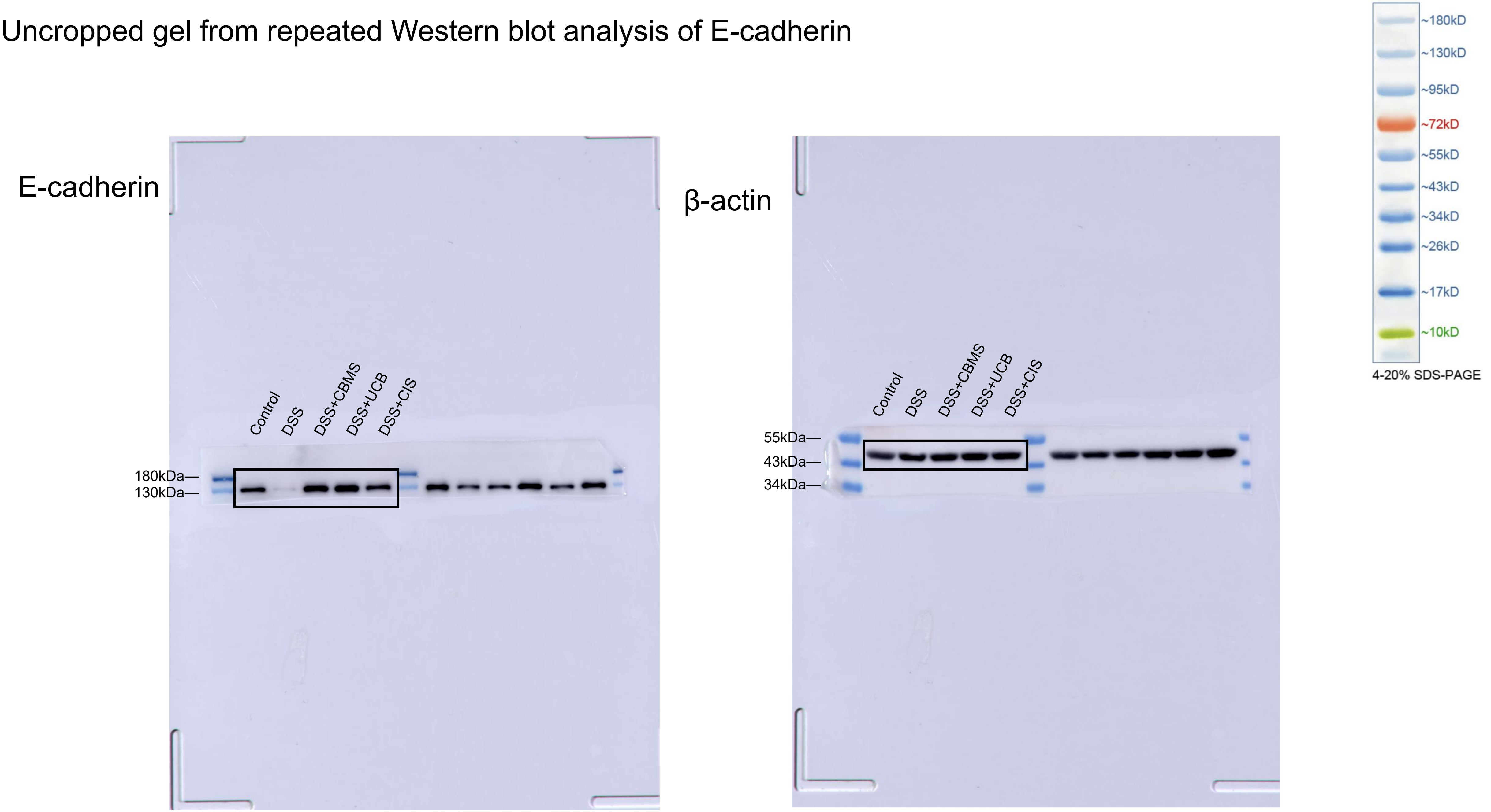

Uncropped gel from repeated Western blot analysis of Occludin

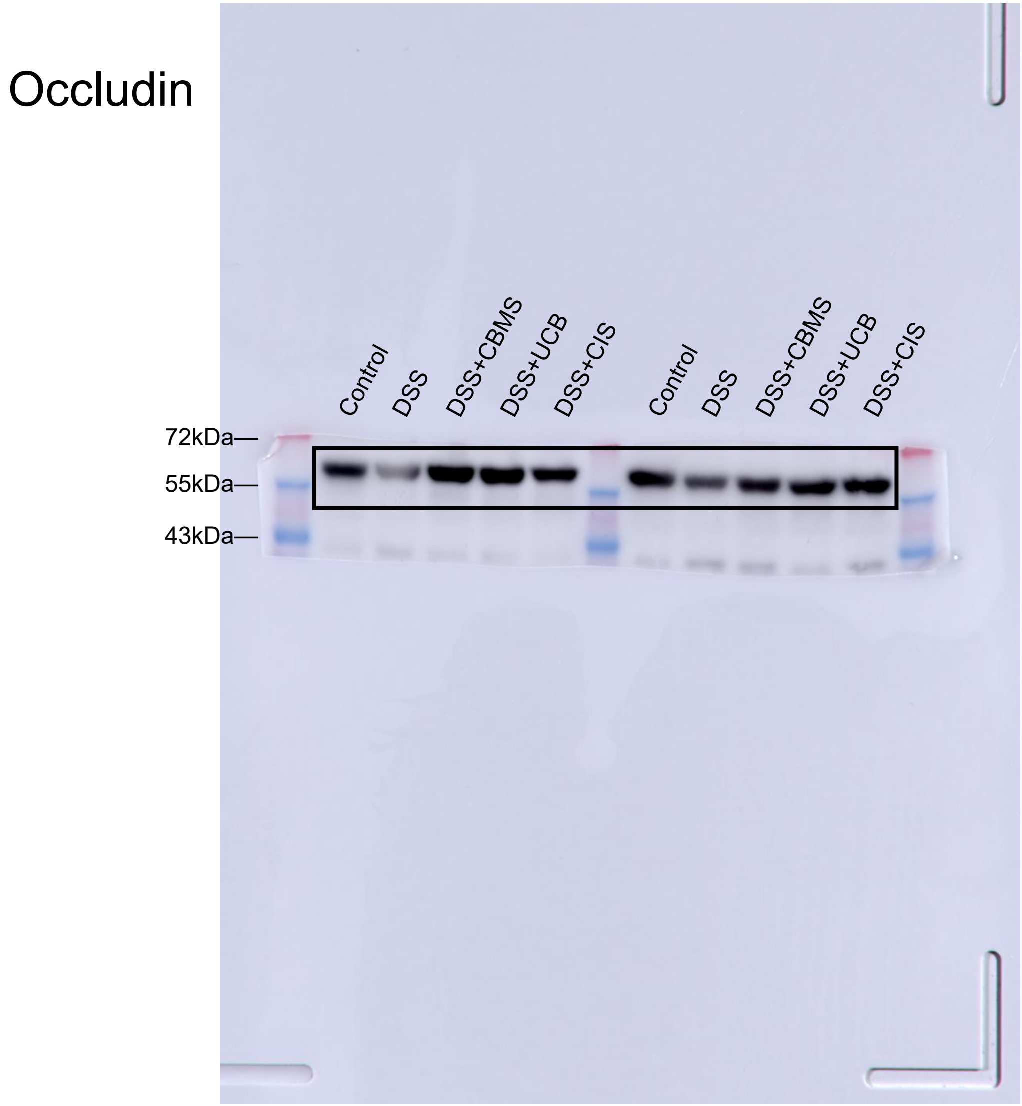

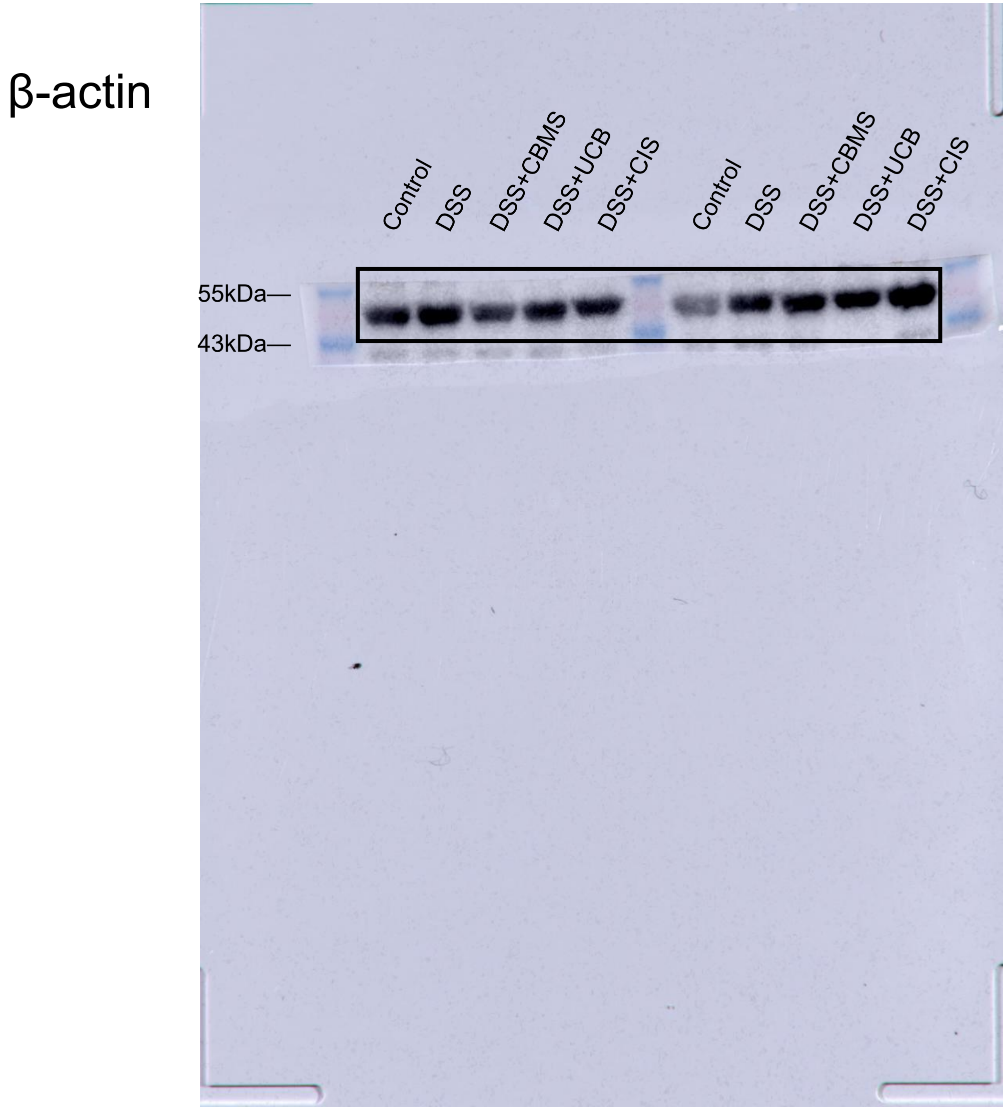

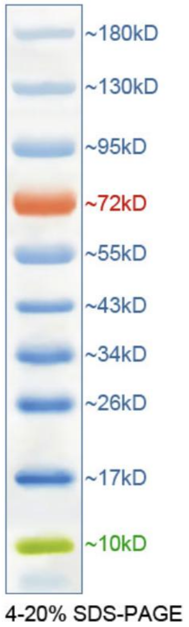

Uncropped gels for Westem Blots in Figure 4 of Occludin

Occludin

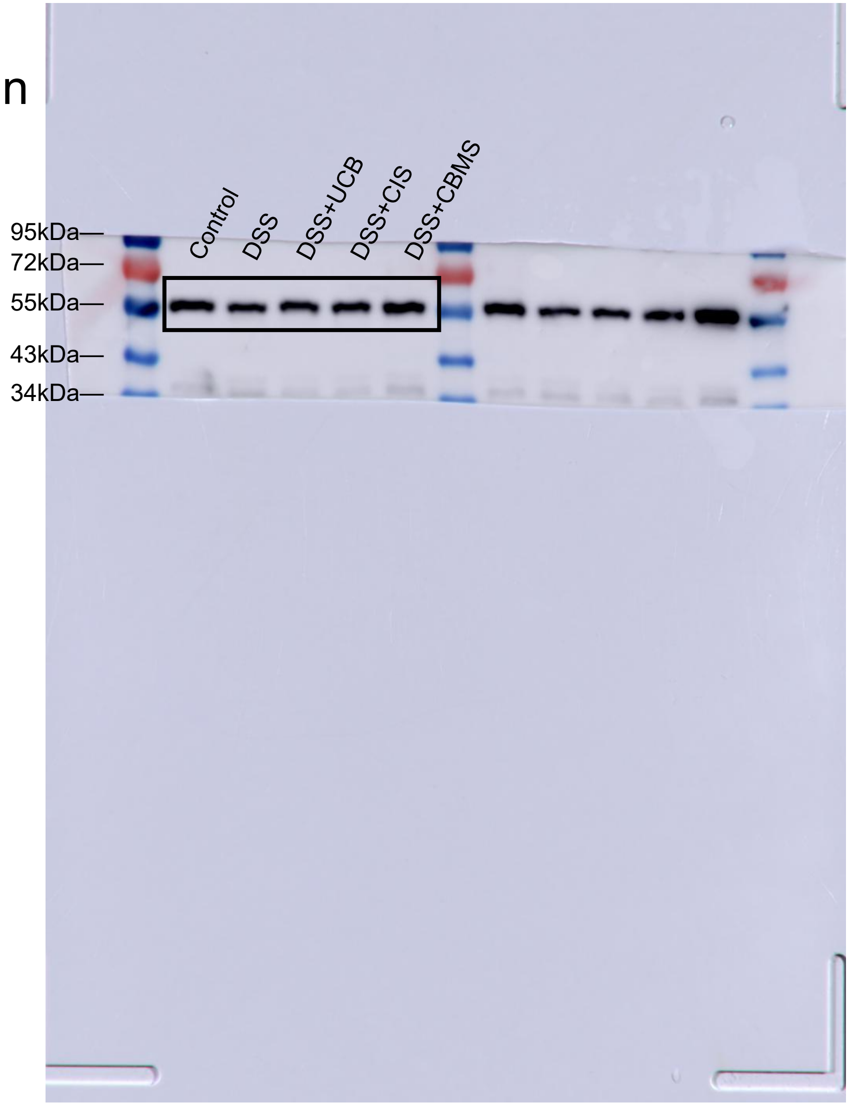

β-actin

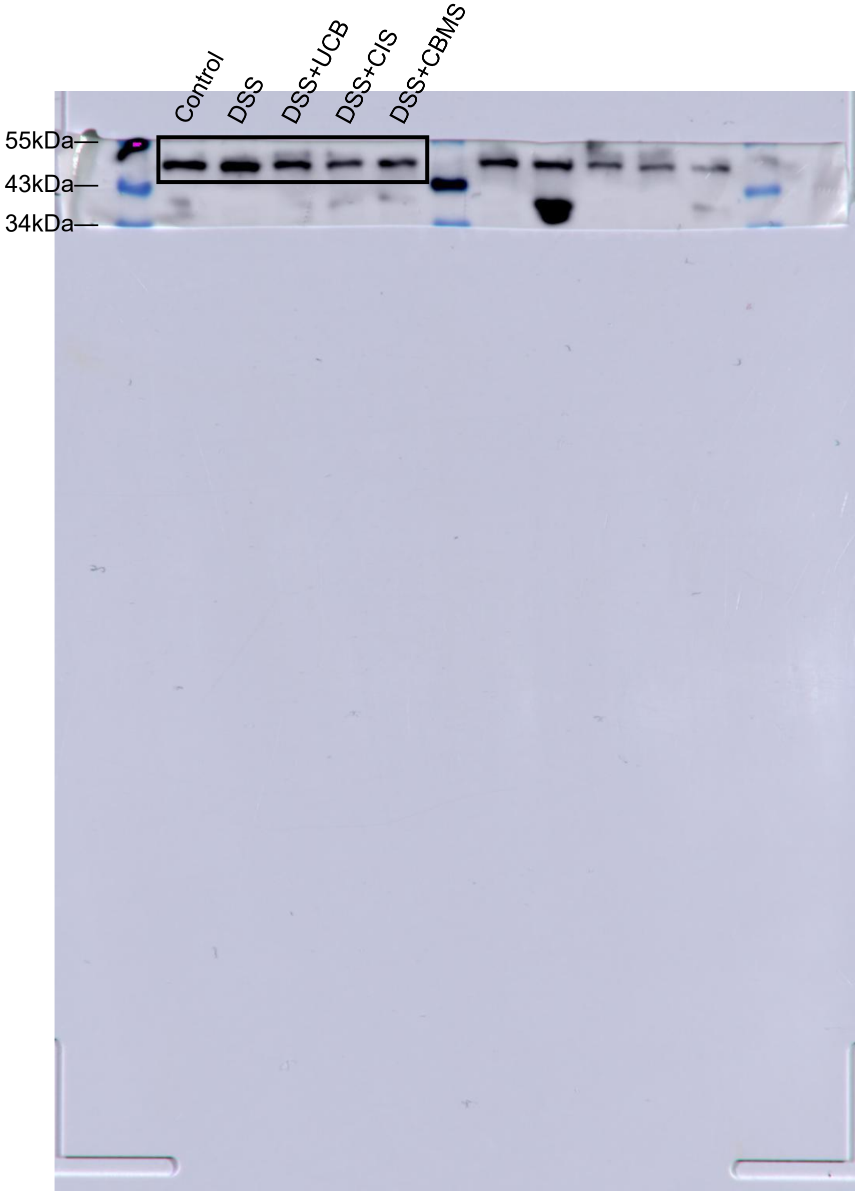

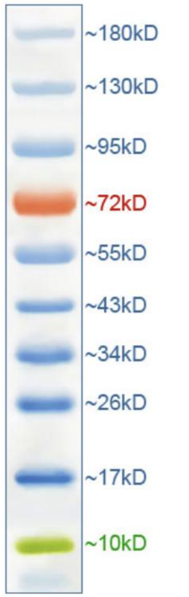

4-20% SDS-PAGE

Uncropped gels for Westem Blots in Figure 4 of Claudin-1

Claudin-1

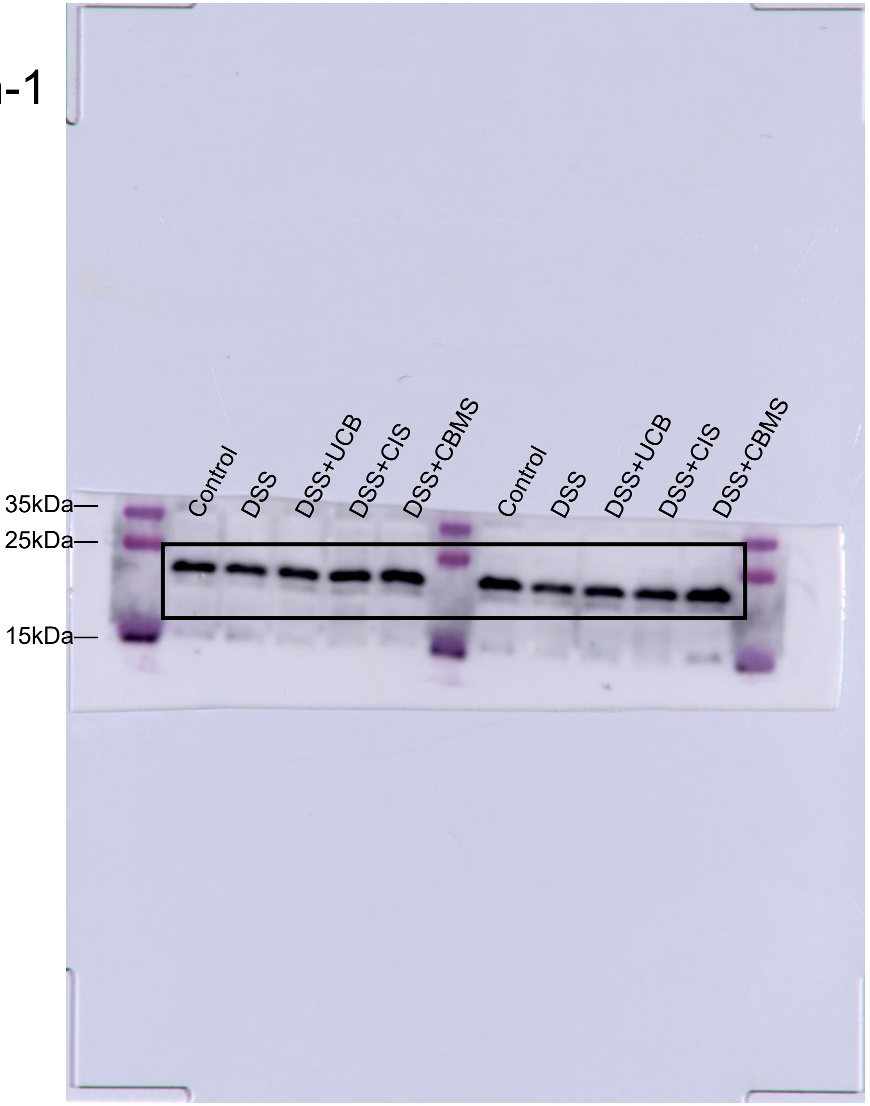

β-actin

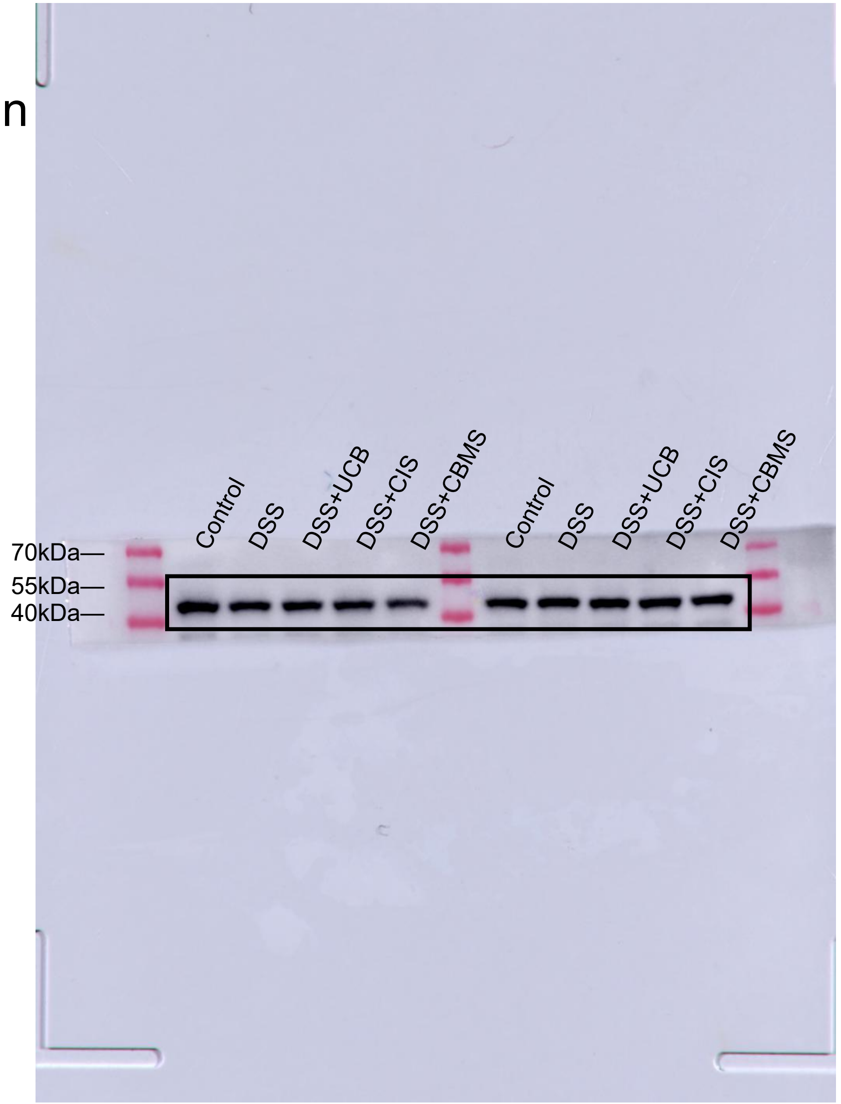

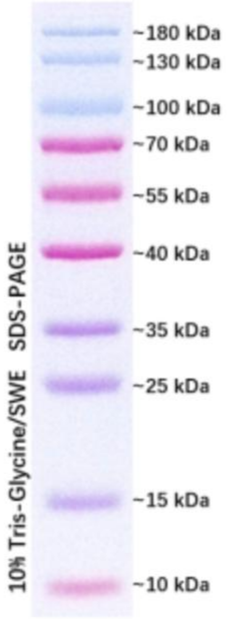

Uncropped gel from repeated Western blot analysis of Claudin-1

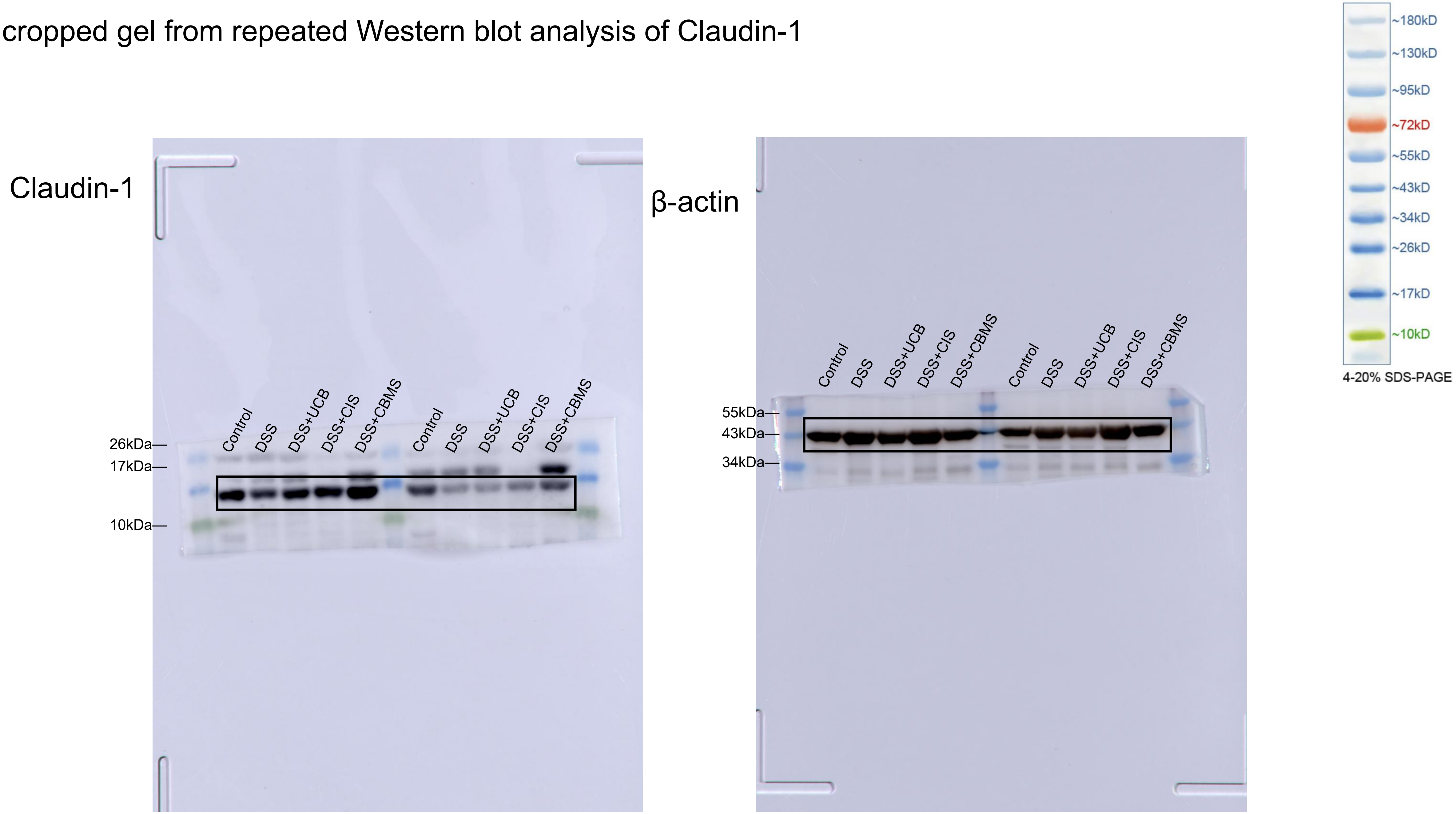

Uncropped gel from repeated Western blot analysis of Claudin-1

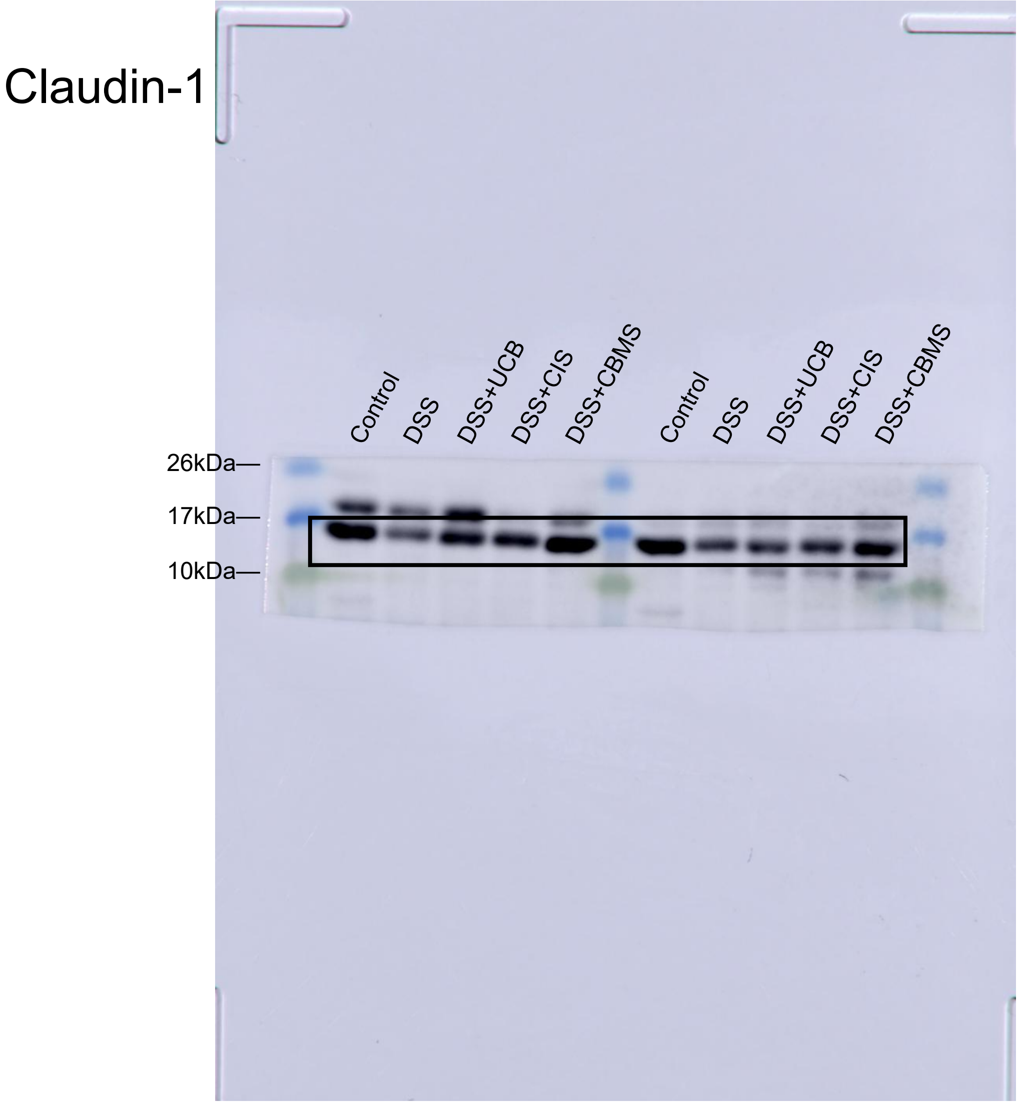

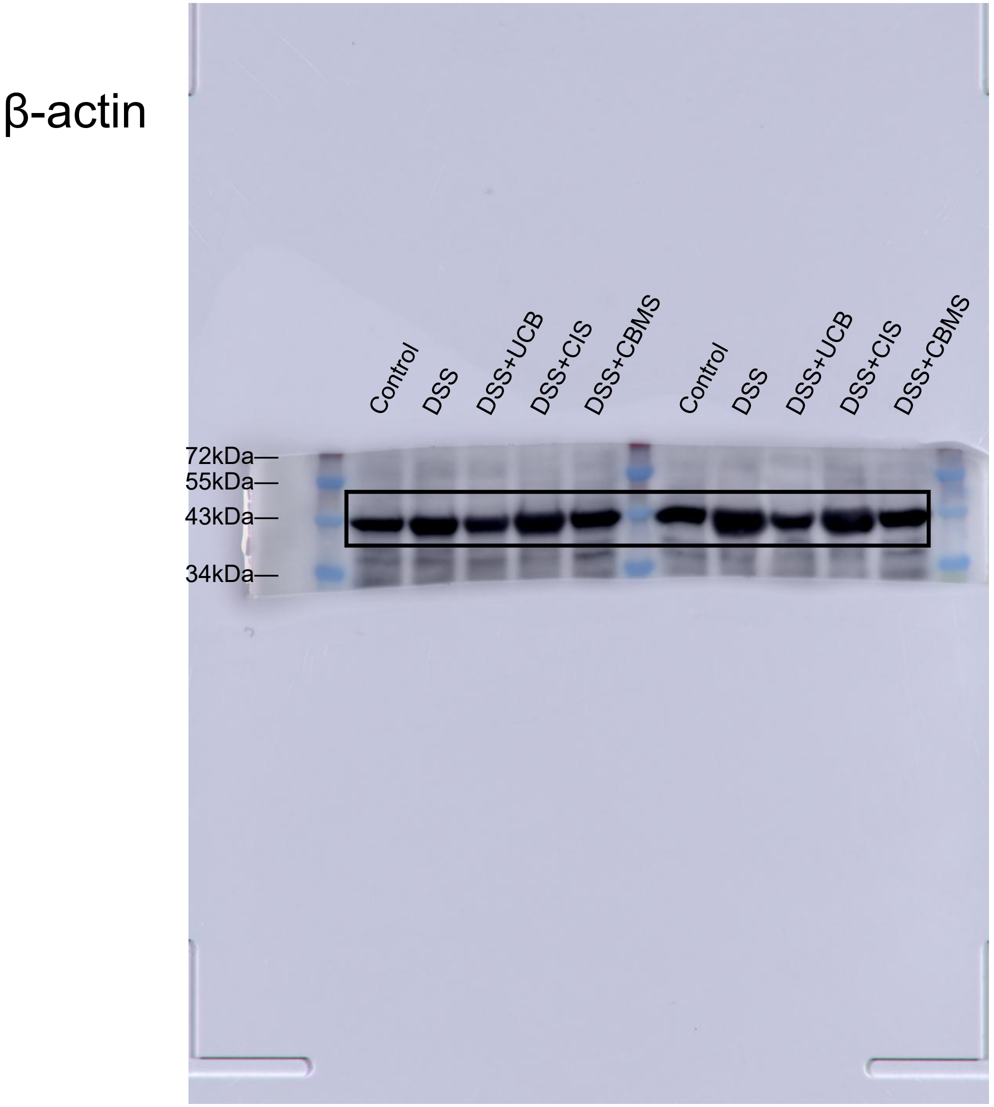

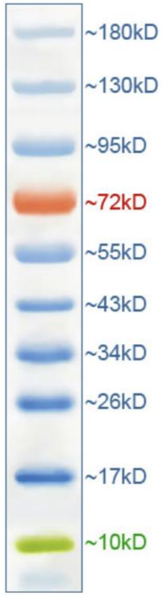

4-20% SDS-PAGE

Uncropped gels for Westem Blots in Figure 4 of β-catenin

β-catenin

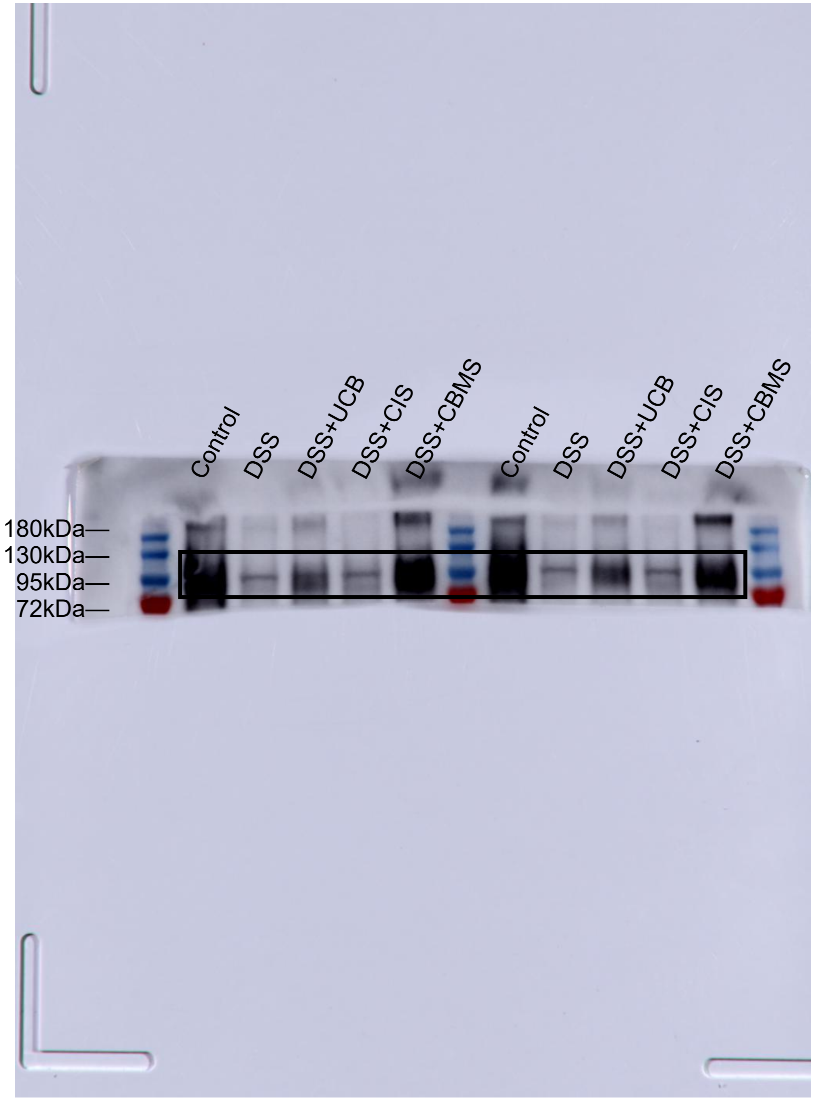

β-actin

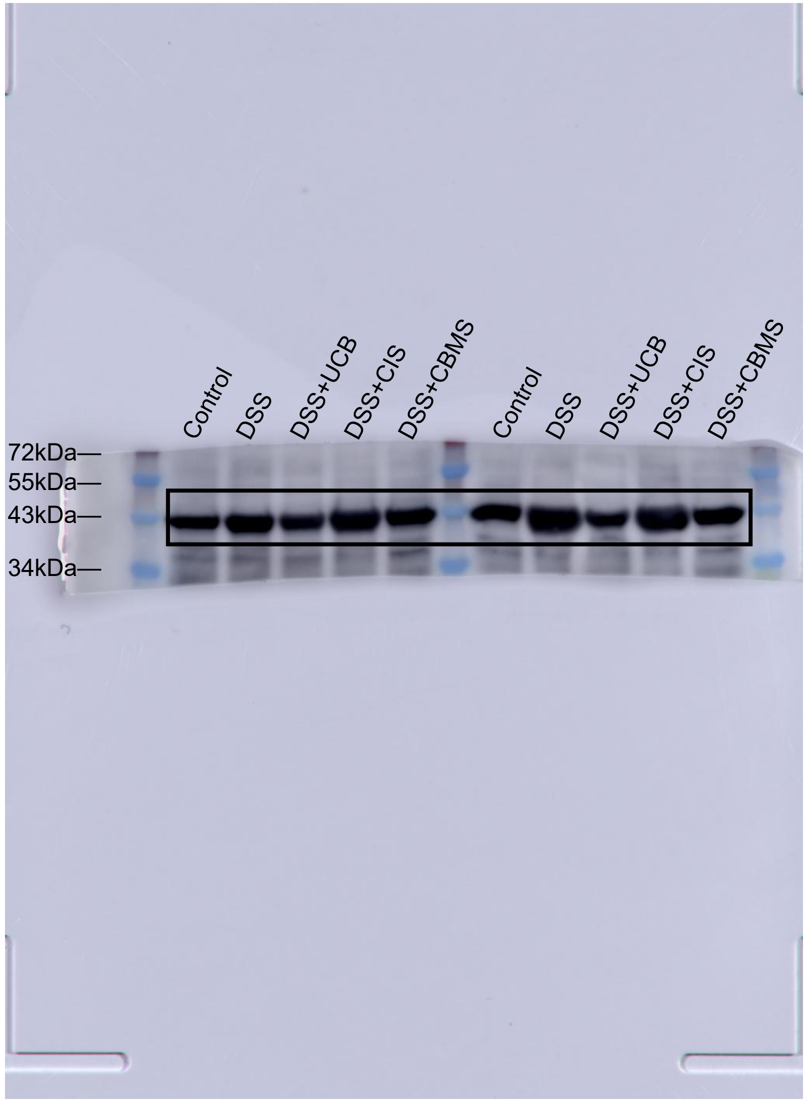

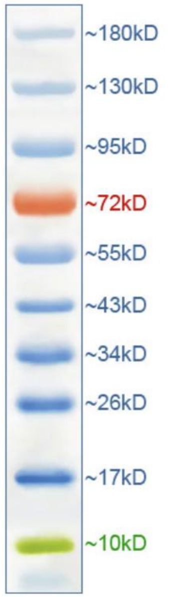

4-20% SDS-PAGE

Uncropped gel from repeated Western blot analysis of β-catenin

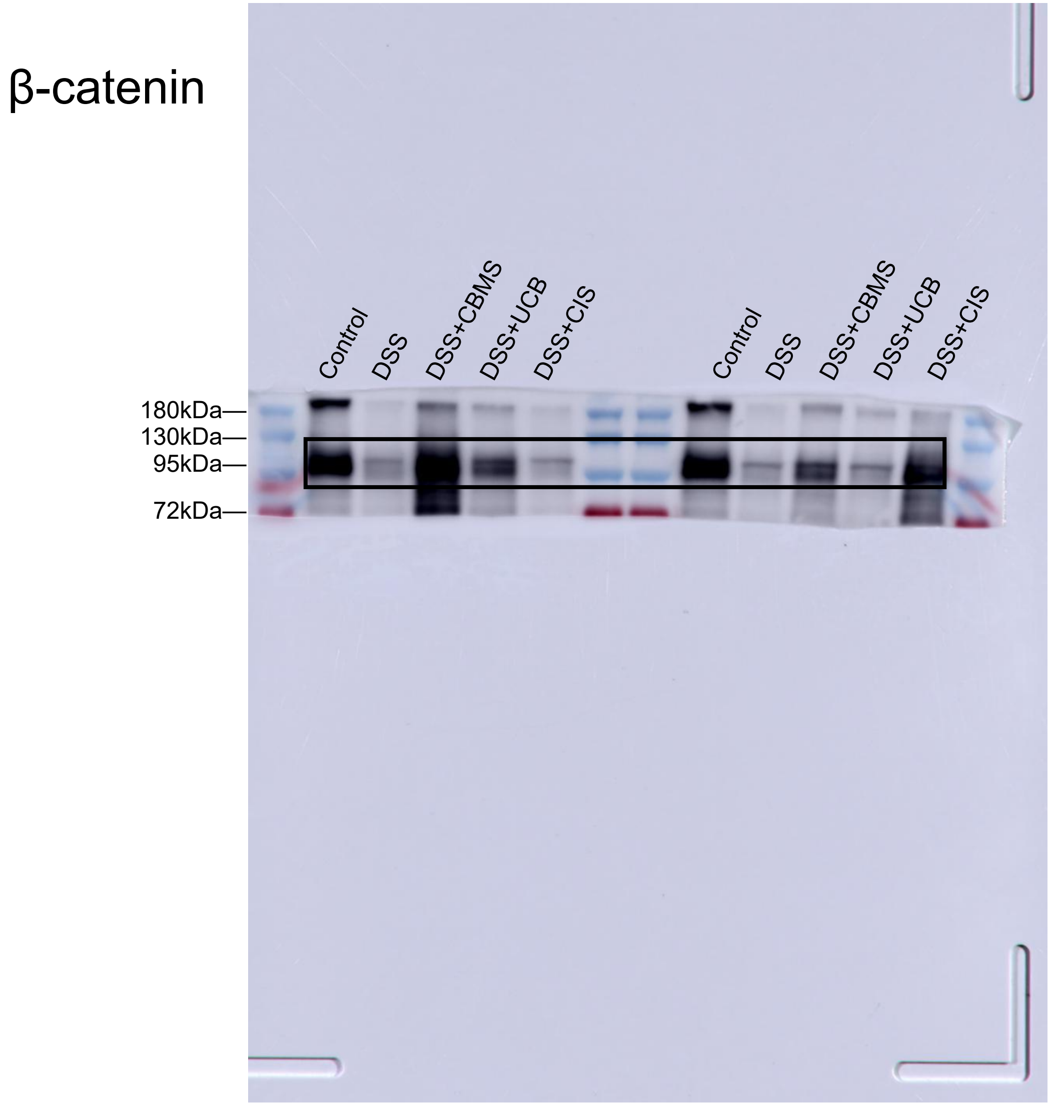

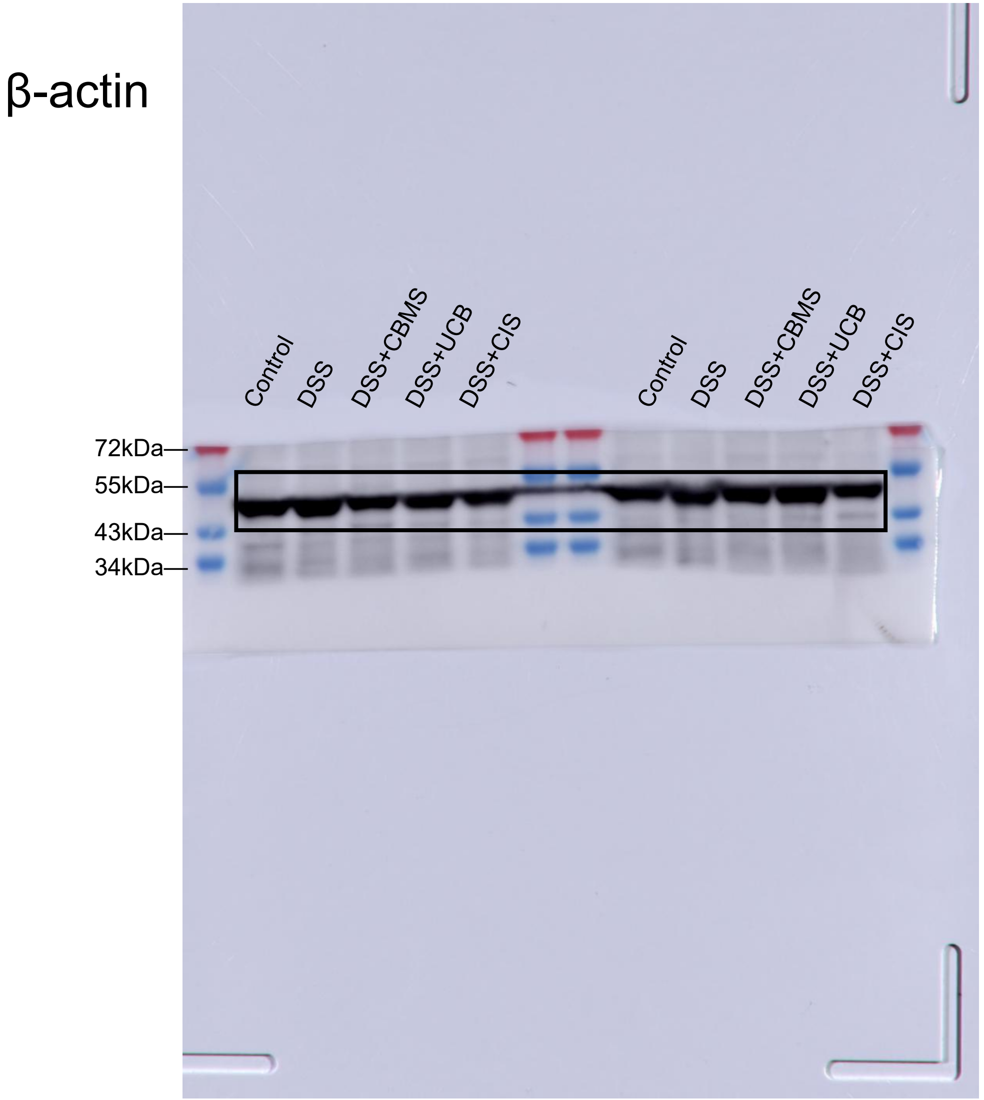

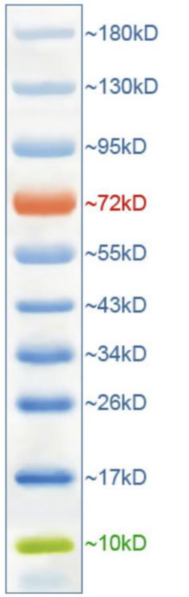

Uncropped gels for Westem Blots in Figure 4 of Wnt3a

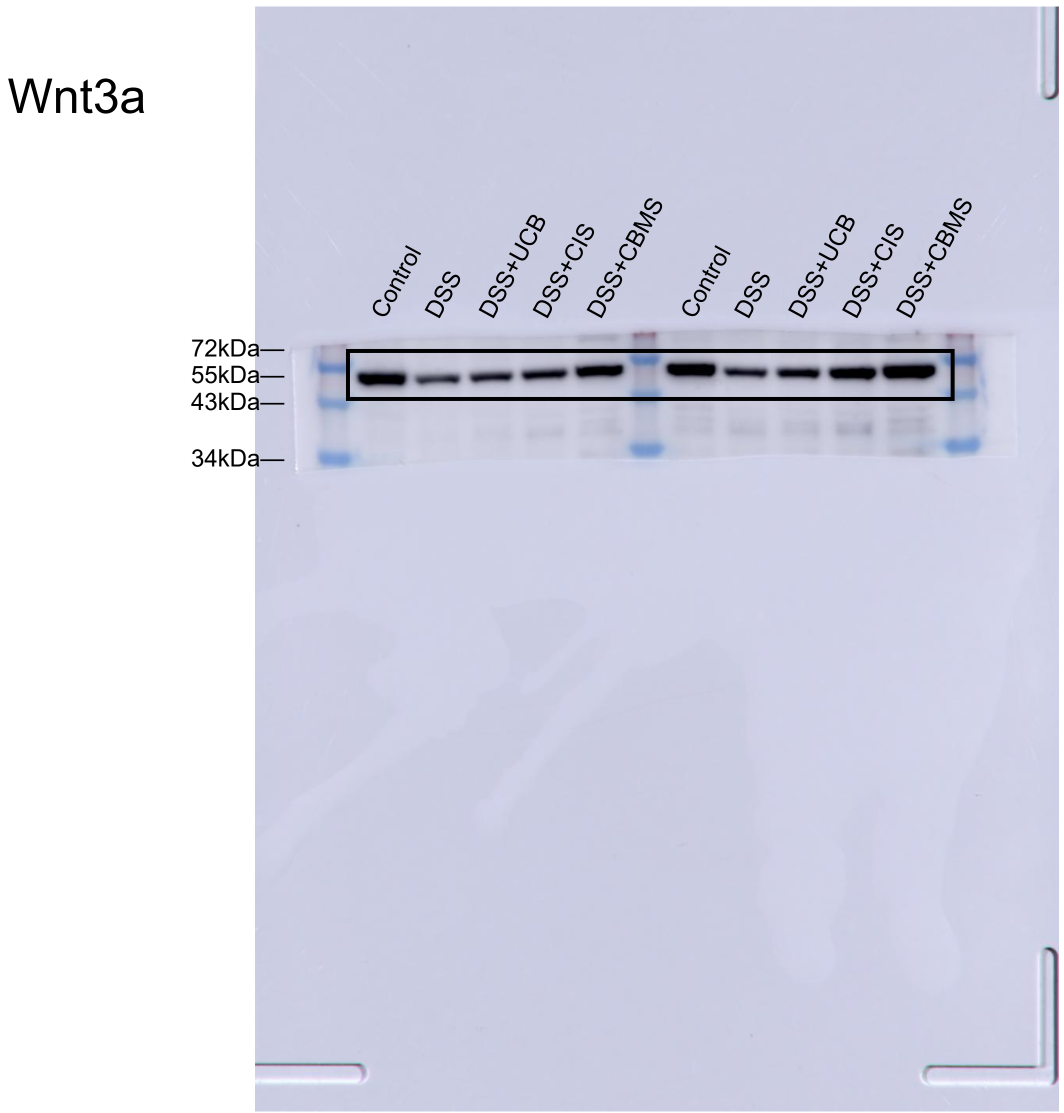

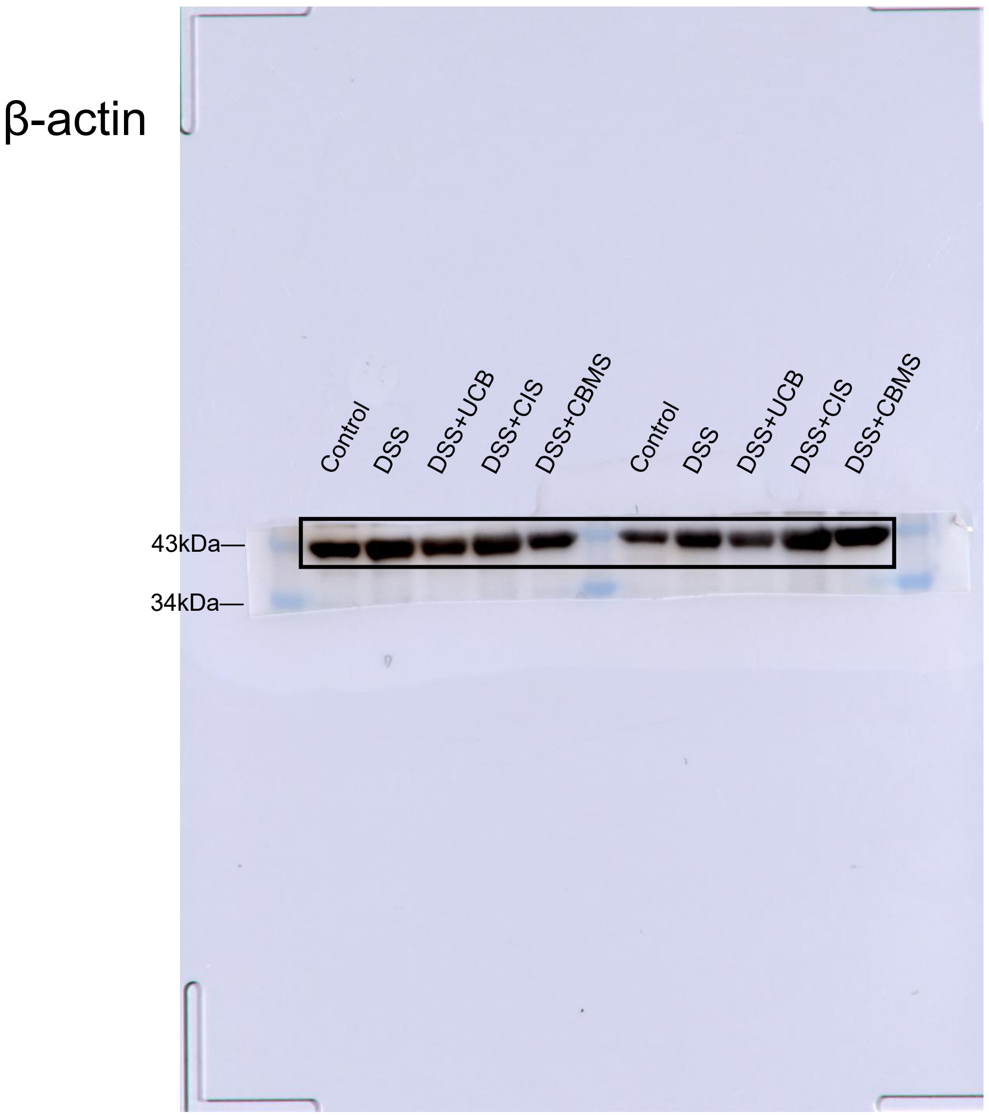

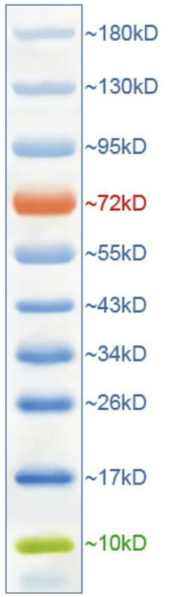

Uncropped gel from repeated Western blot analysis of Wnt3a

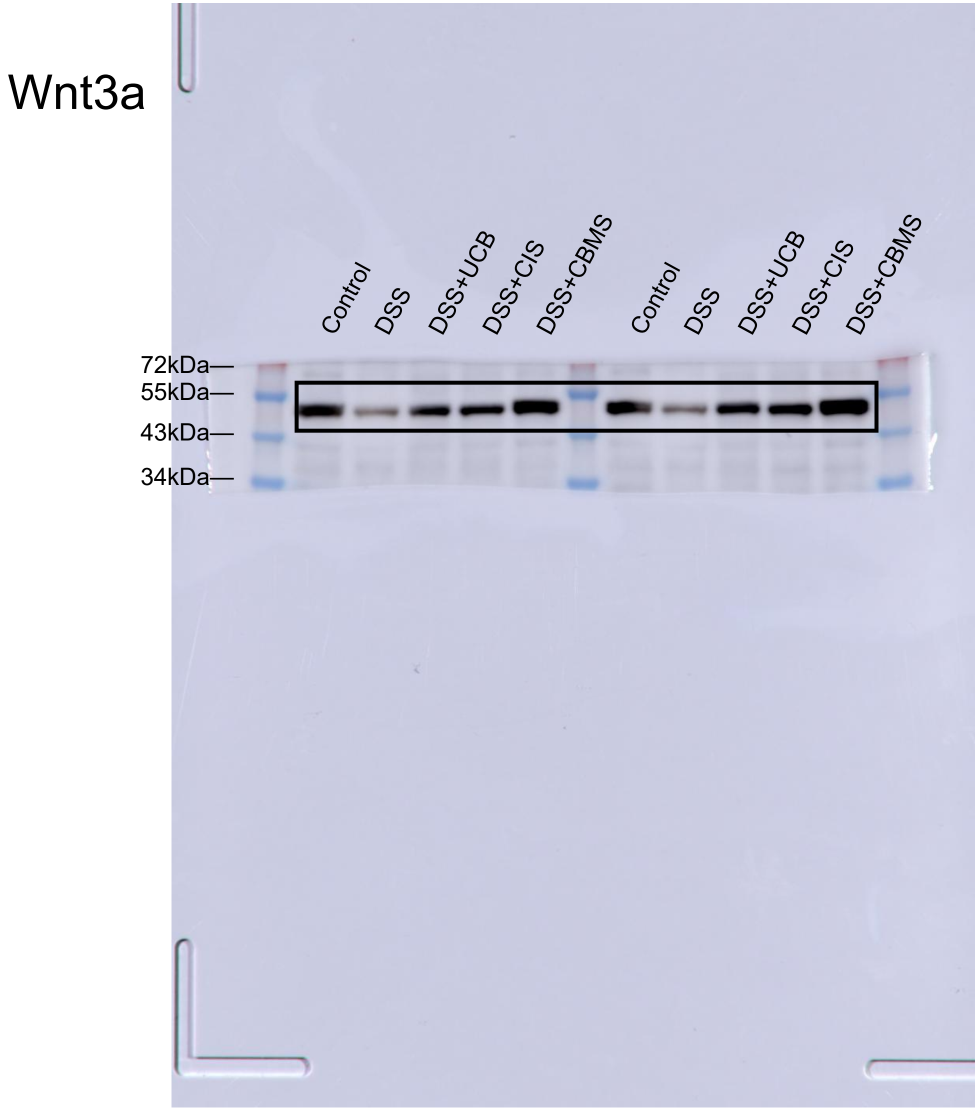

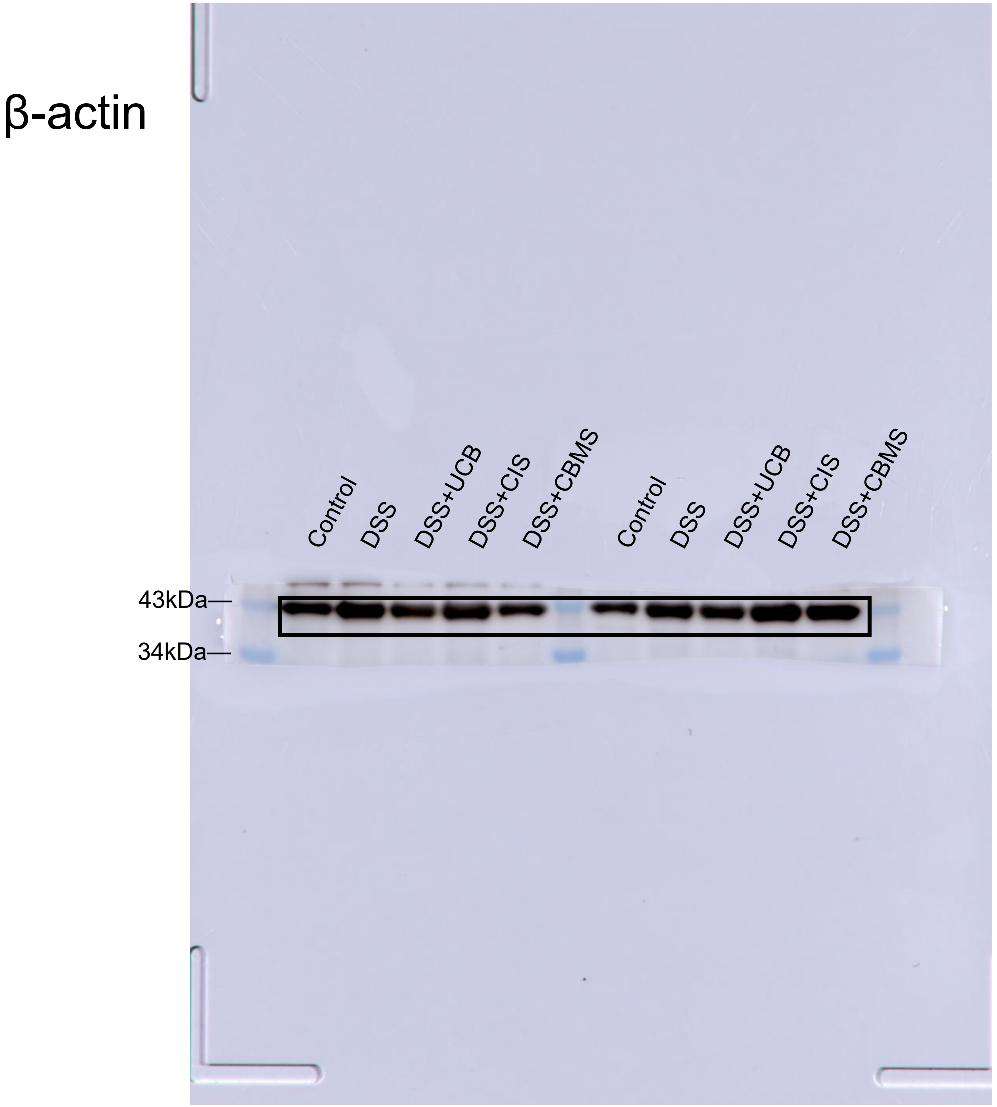

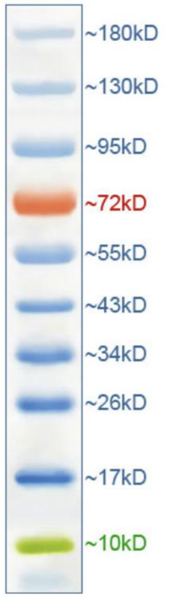

4-20% SDS-PAGE

Uncropped gels for Westem Blots in Figure 5 of IL-17A

IL-17A

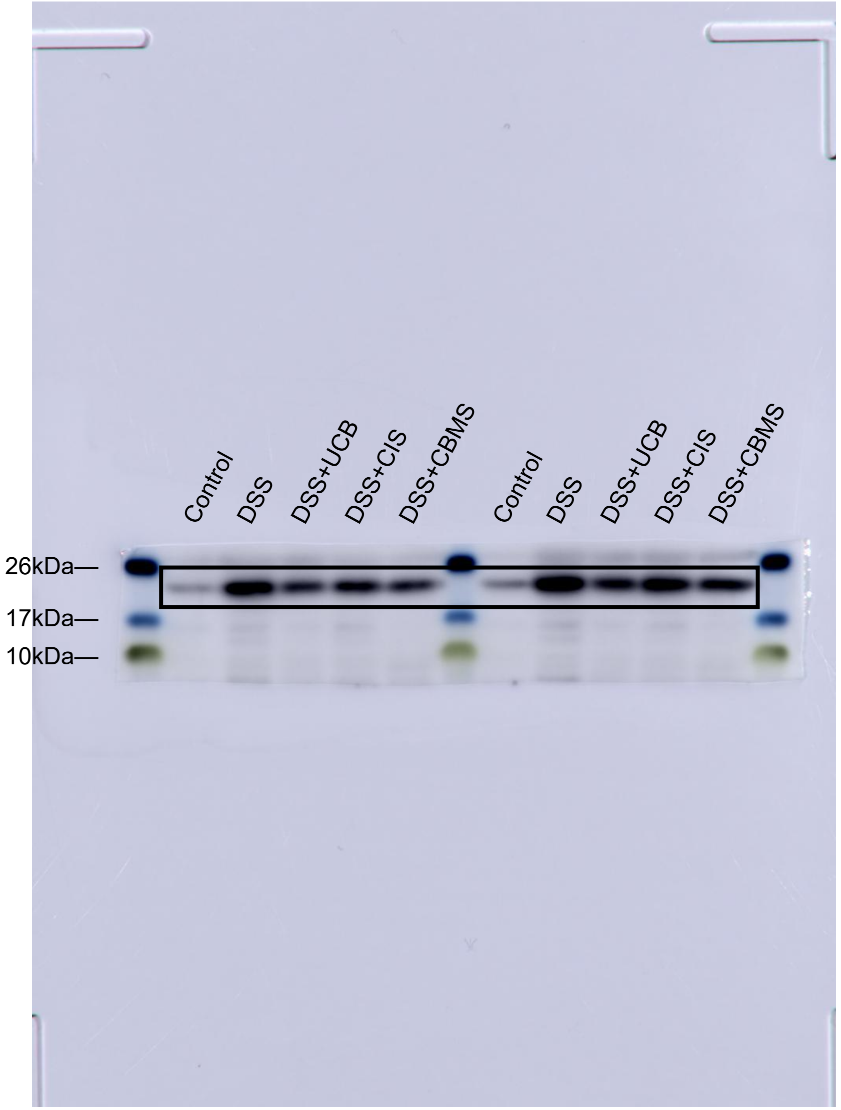

β-actin

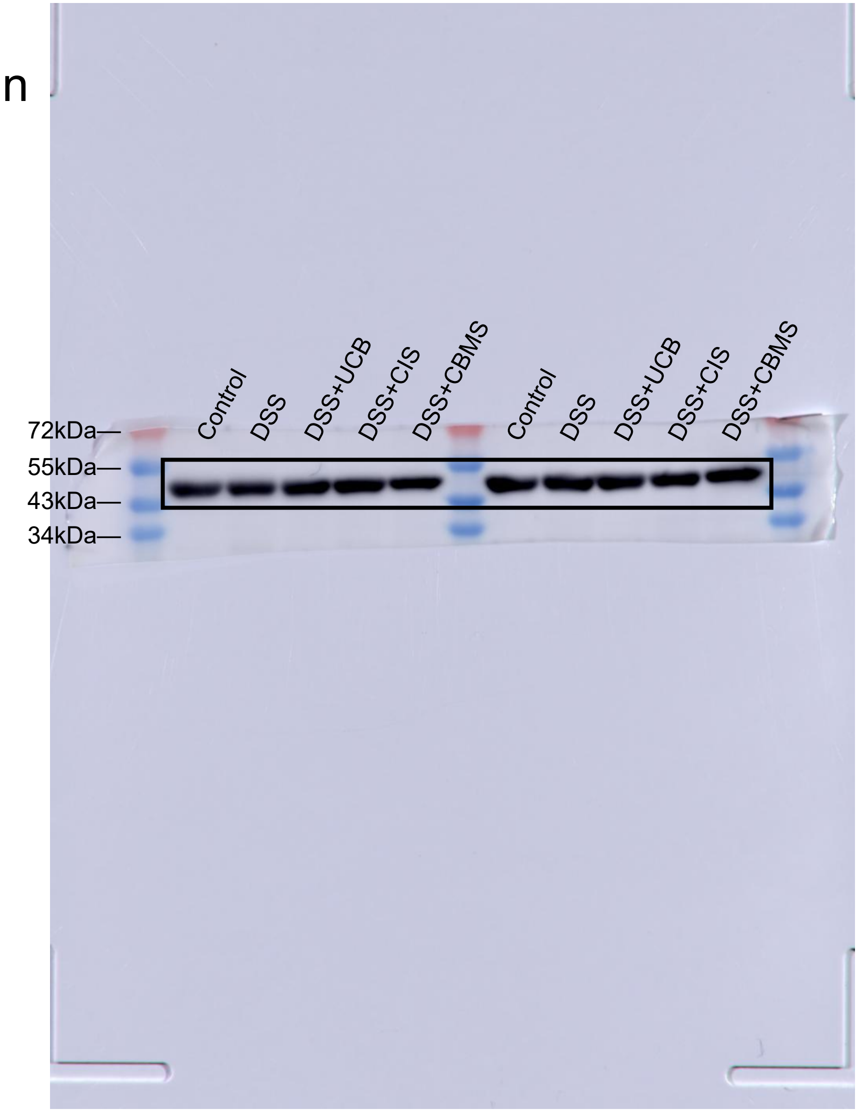

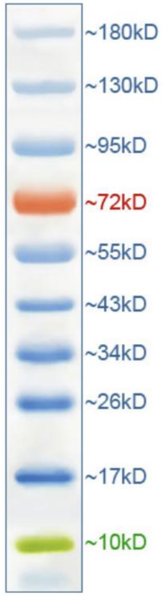

4-20% SDS-PAGE

Uncropped gel from repeated Western blot analysis of IL-17A

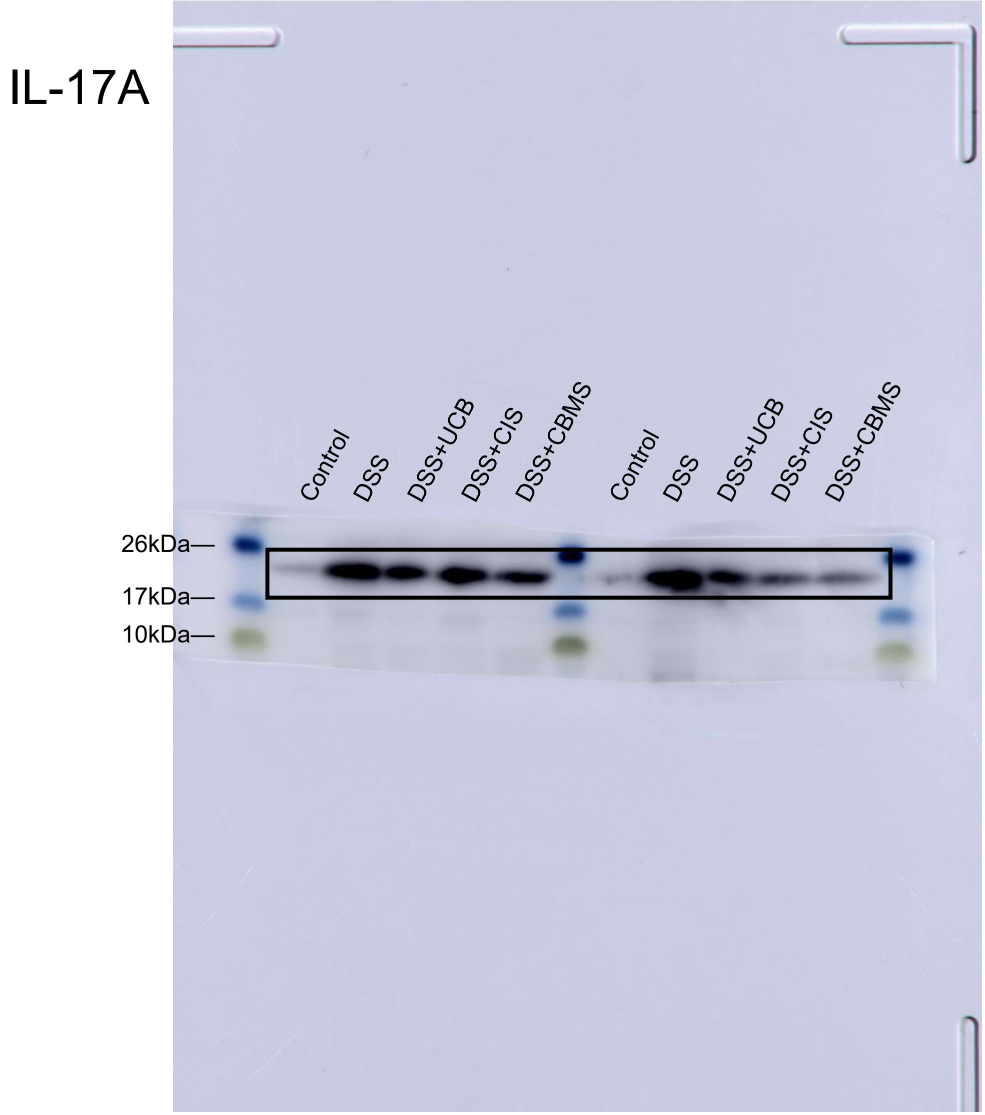

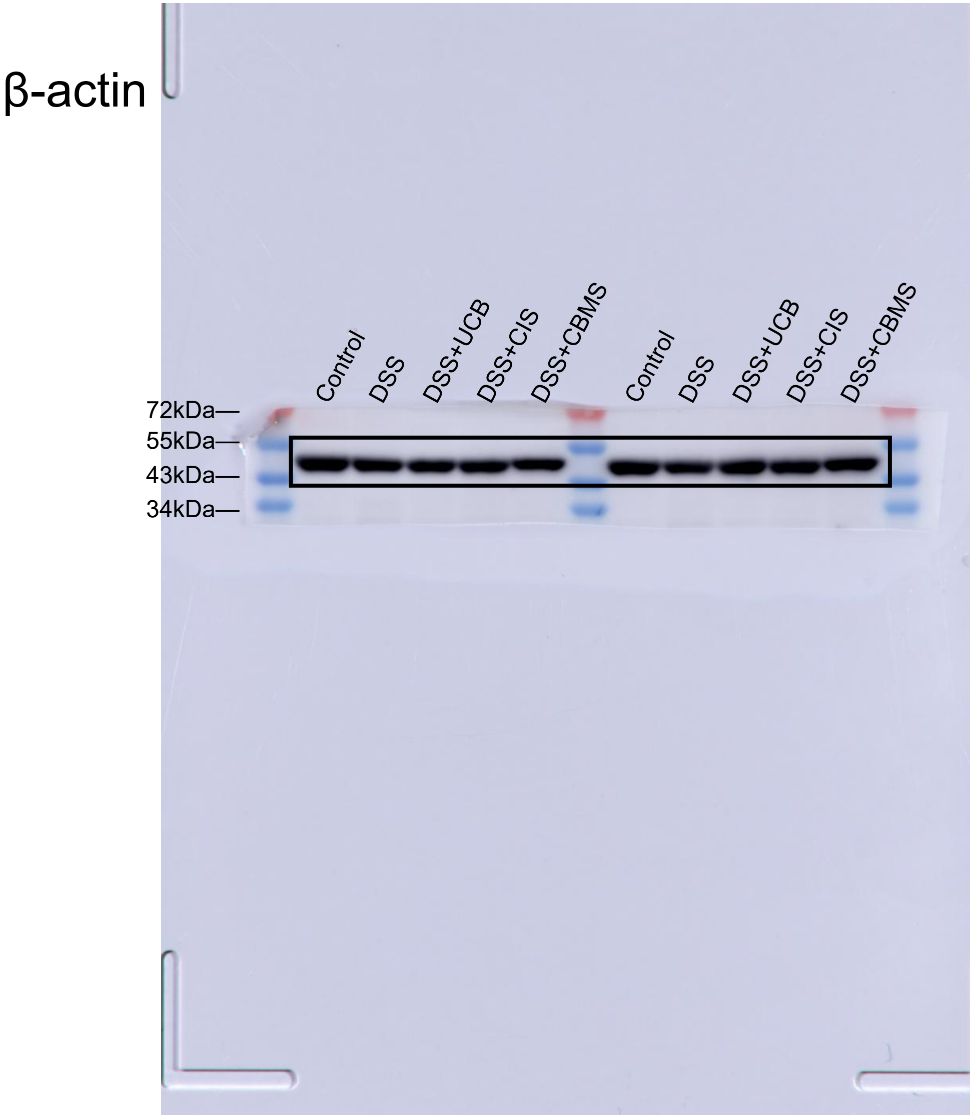

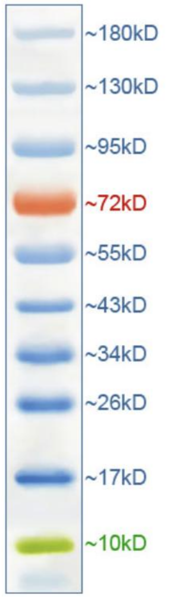

4-20% SDS-PAGE

Uncropped gel from repeated Western blot analysis of IL-17A

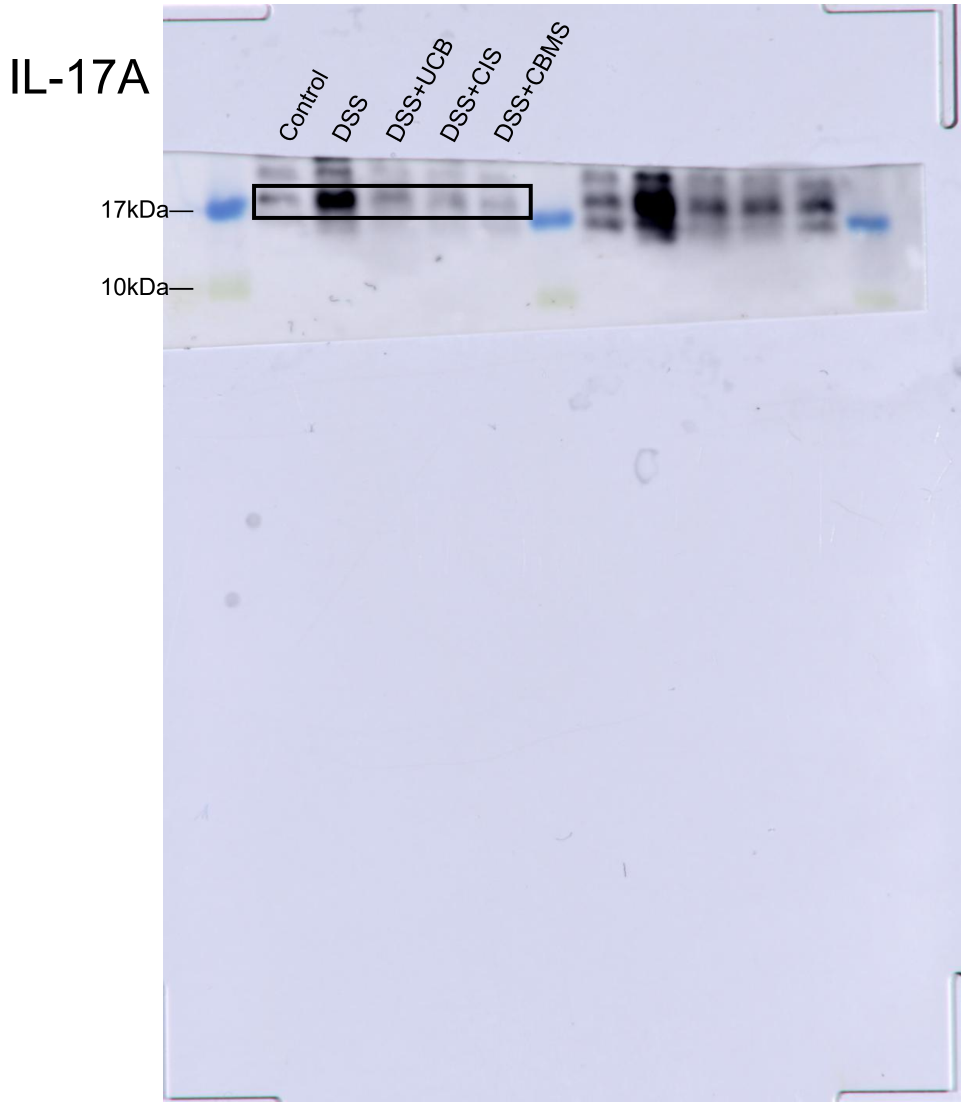

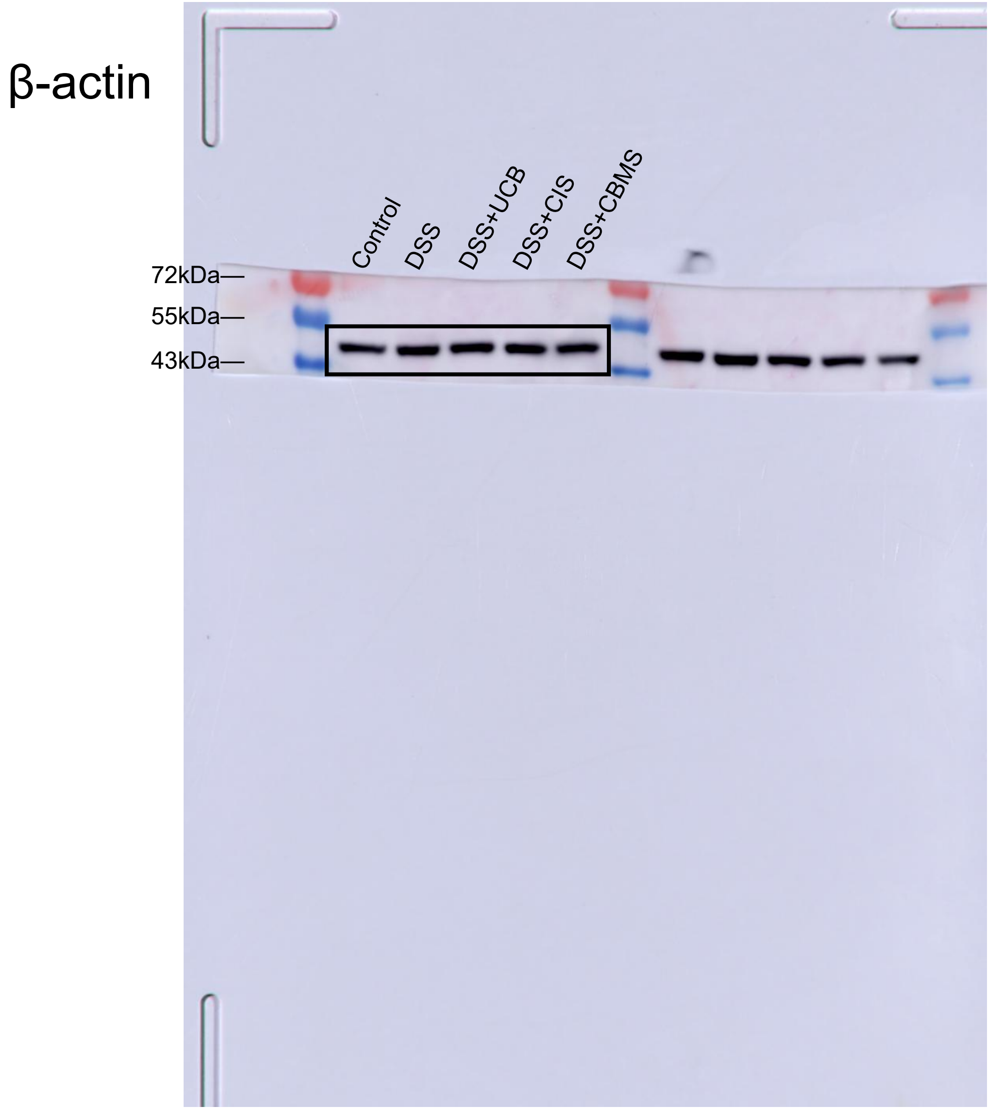

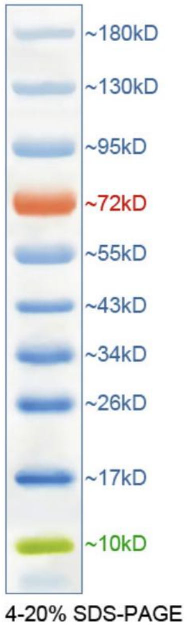

Uncropped gels for Westem Blots in Figure 5 of TRAF6

TRAF6

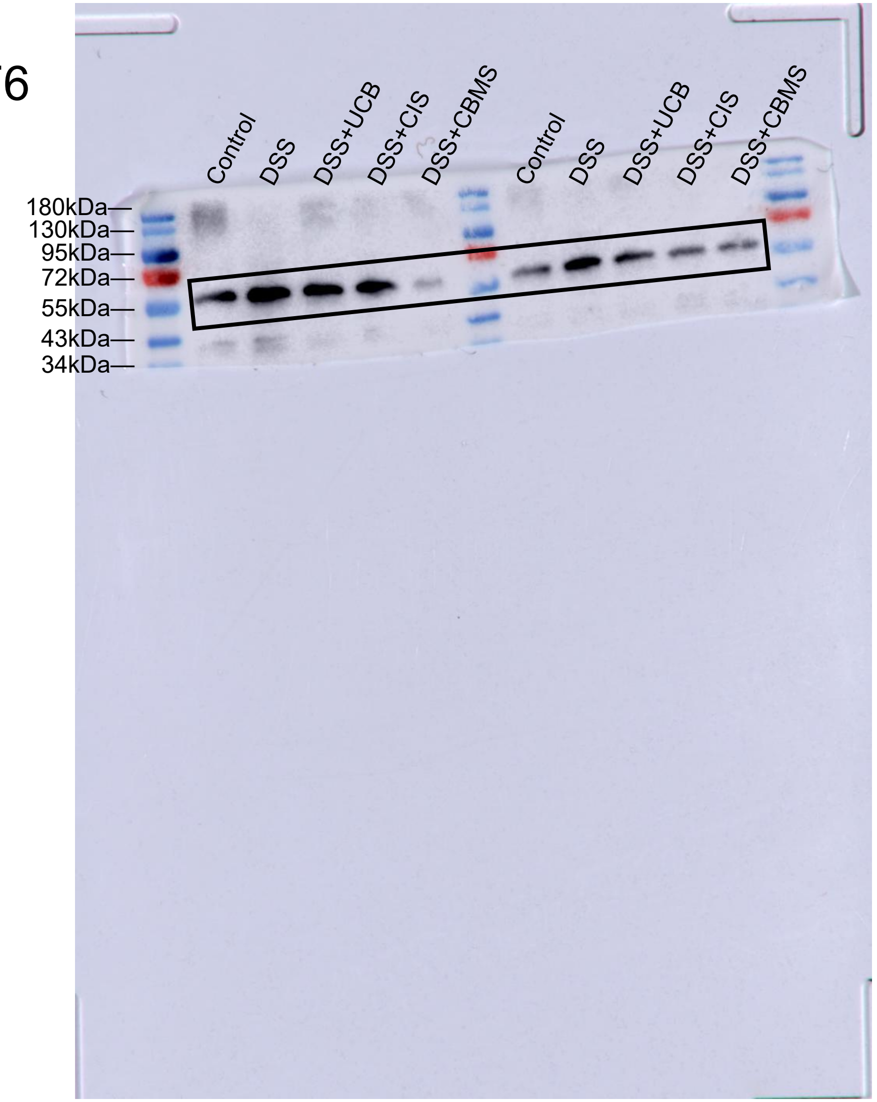

β-actin

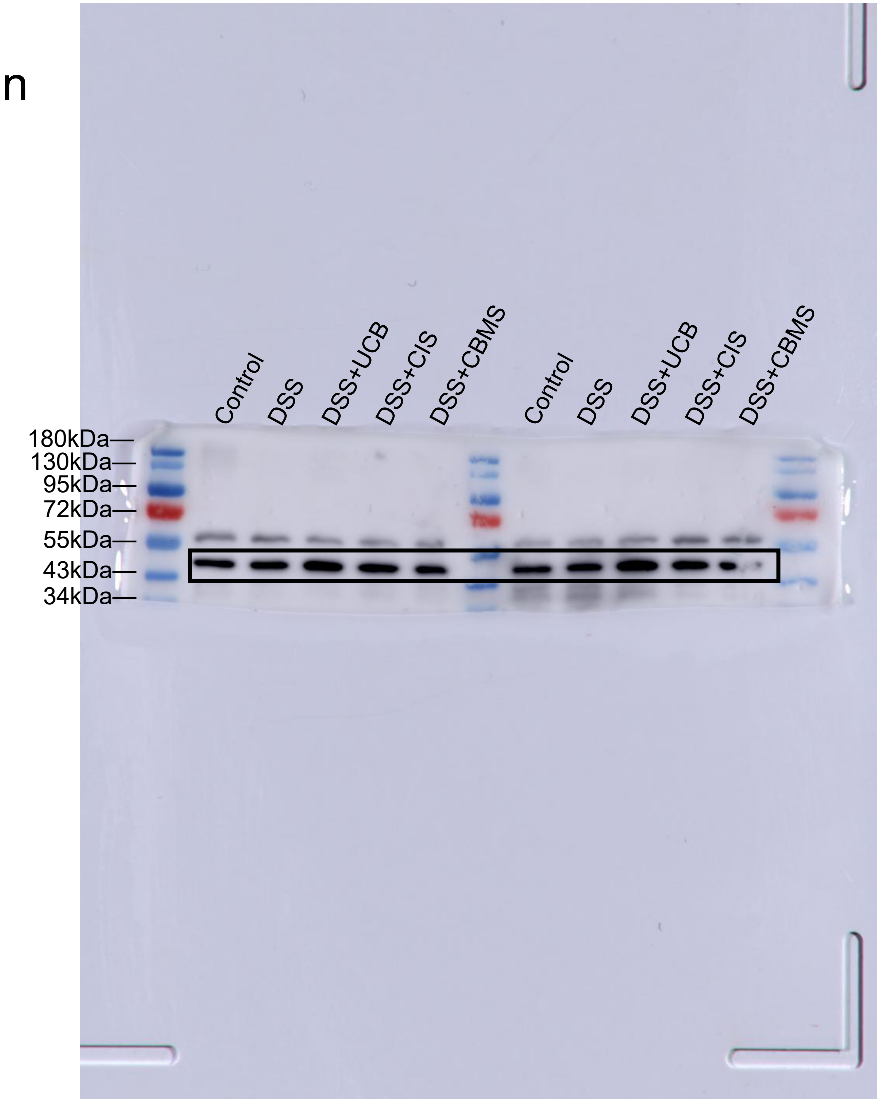

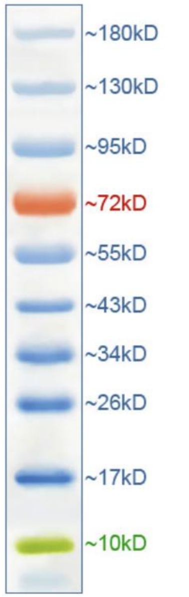

4-20% SDS-PAGE

Uncropped gel from repeated Western blot analysis of TRAF6

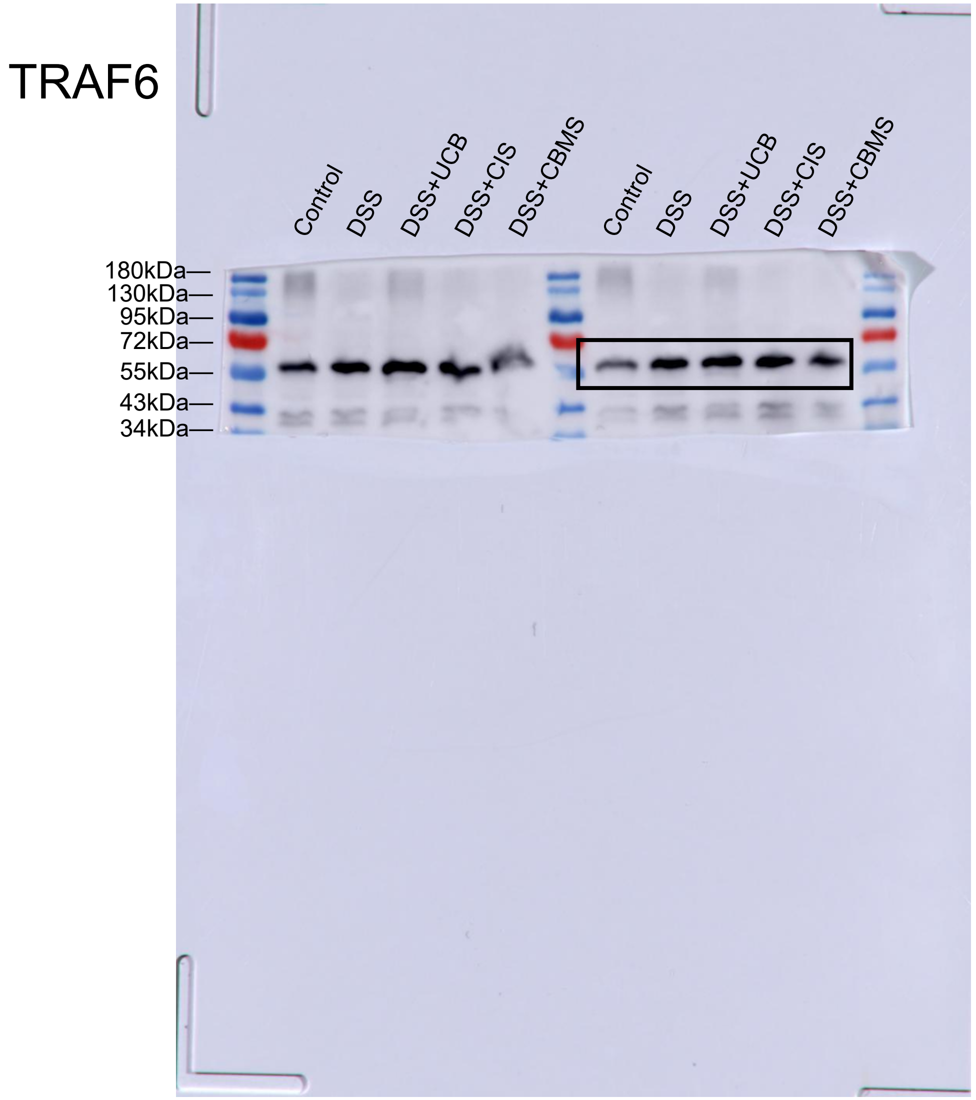

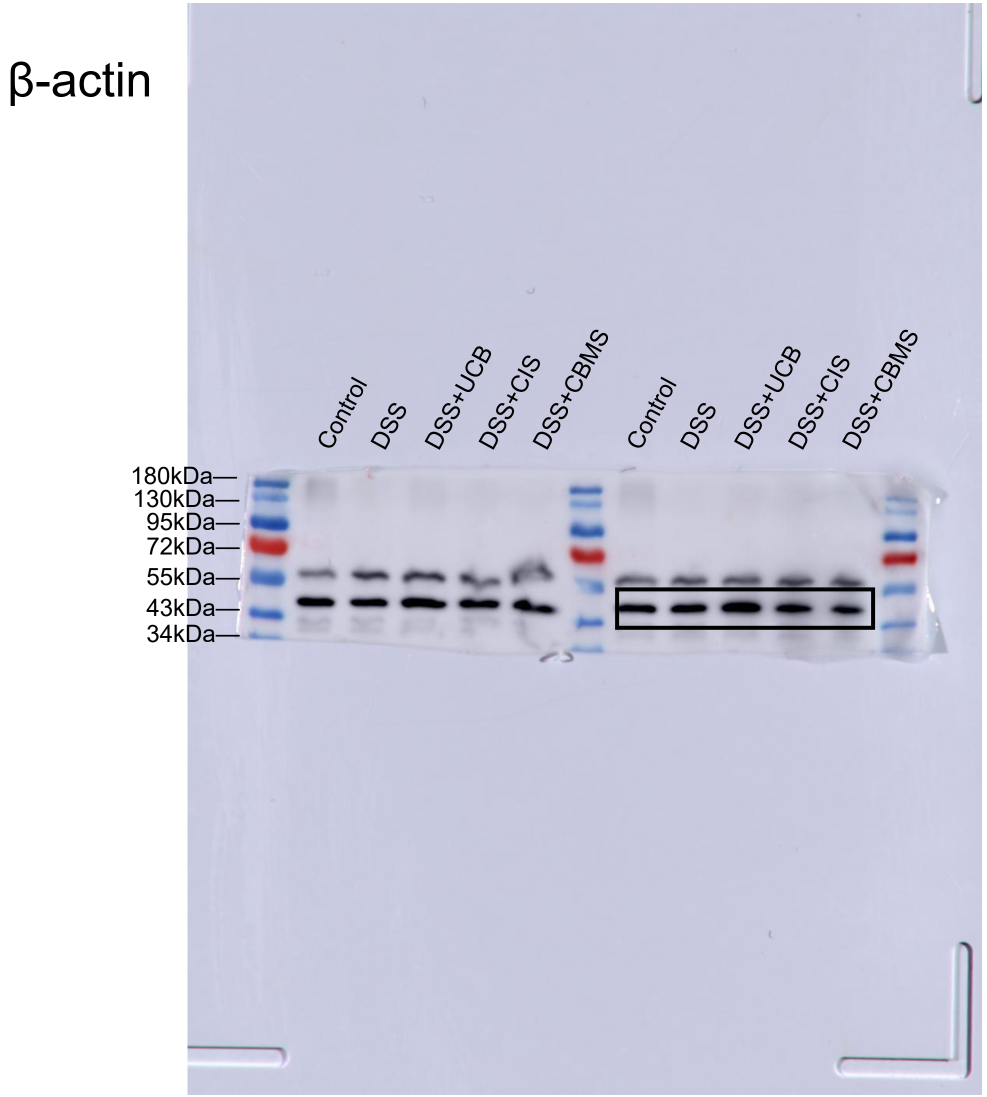

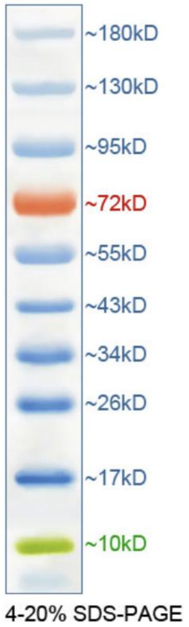

Uncropped gel from repeated Western blot analysis of TRAF6

TRAF6

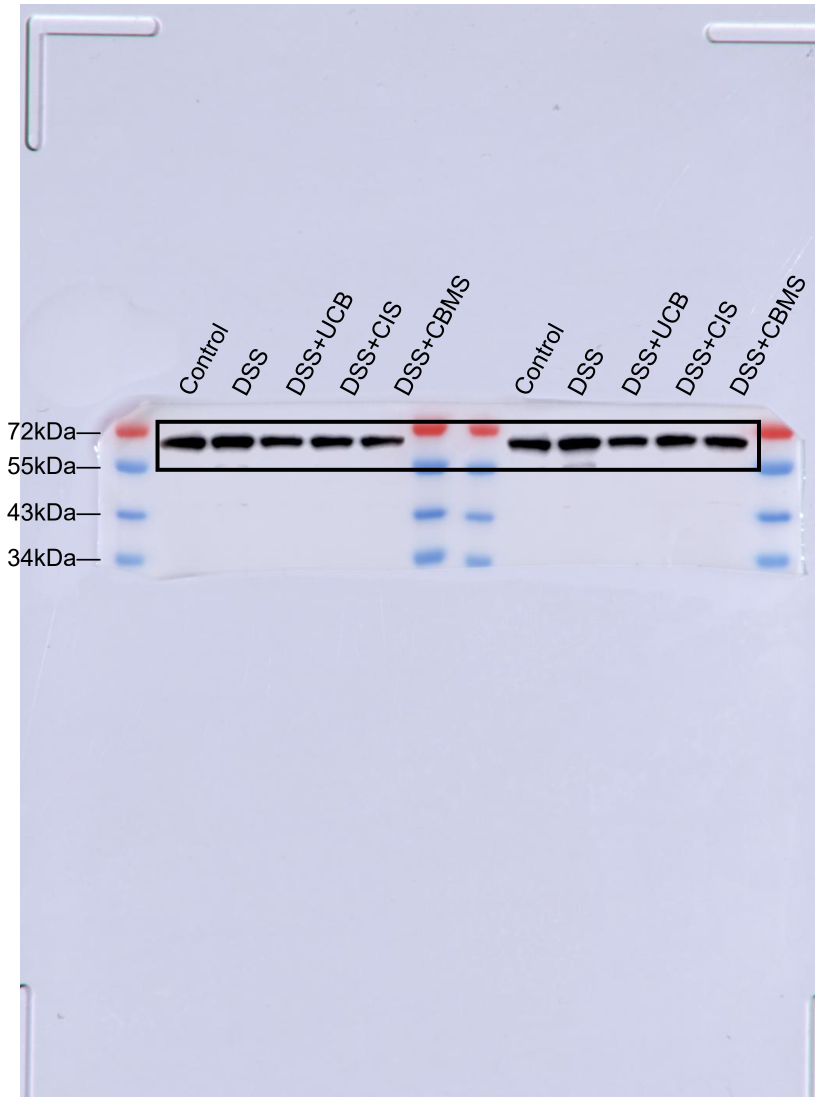

β-actin

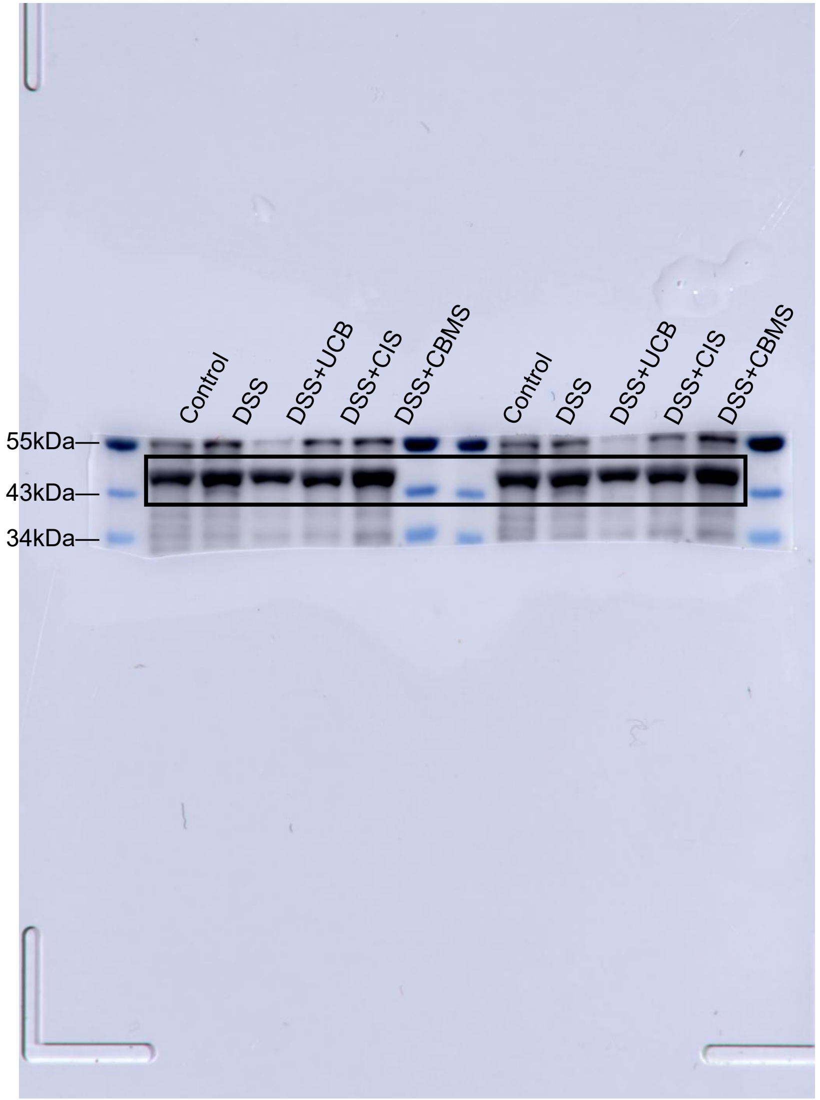

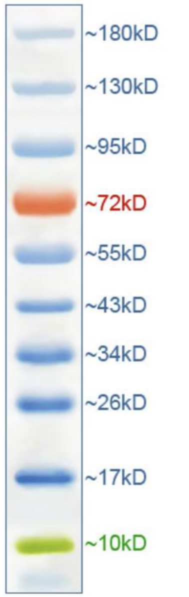

4-20% SDS-PAGE

Uncropped gel from repeated Western blot analysis of TRAF6

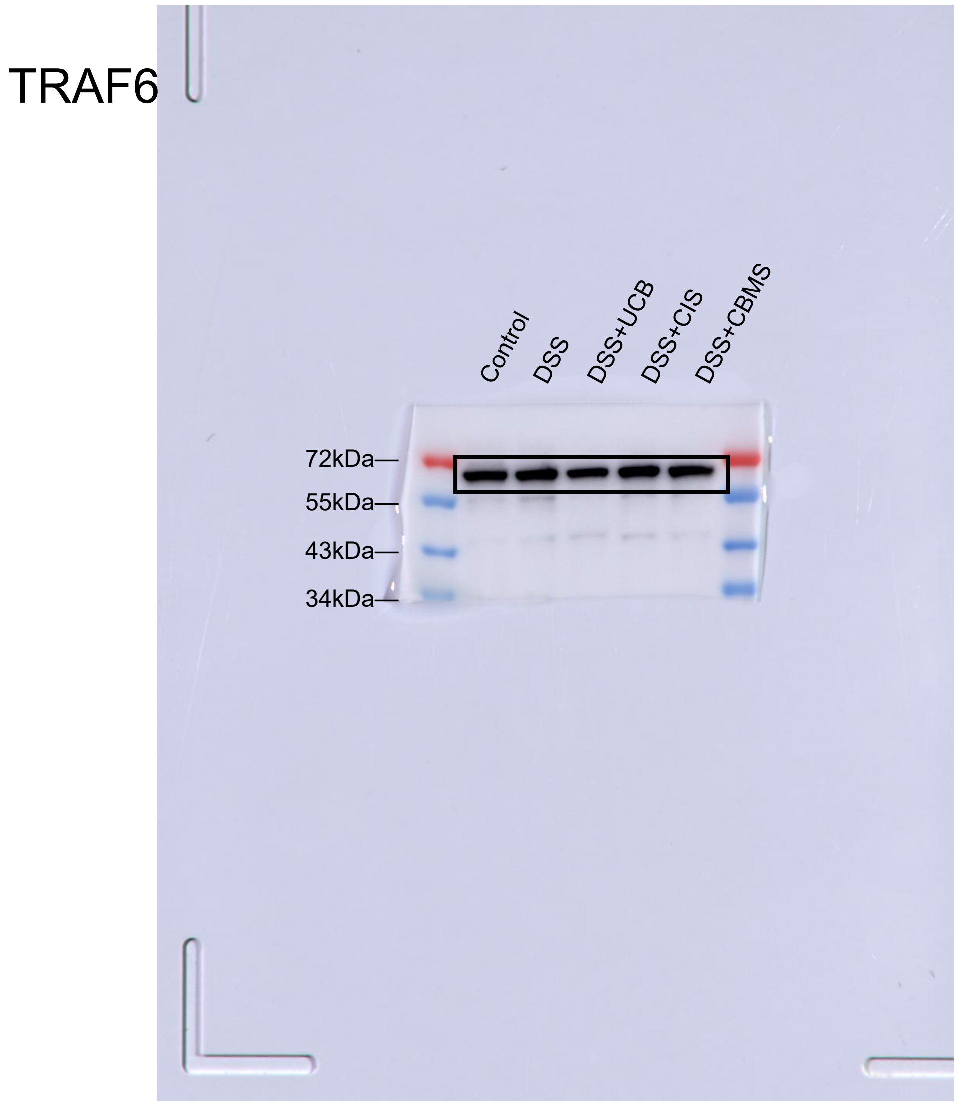

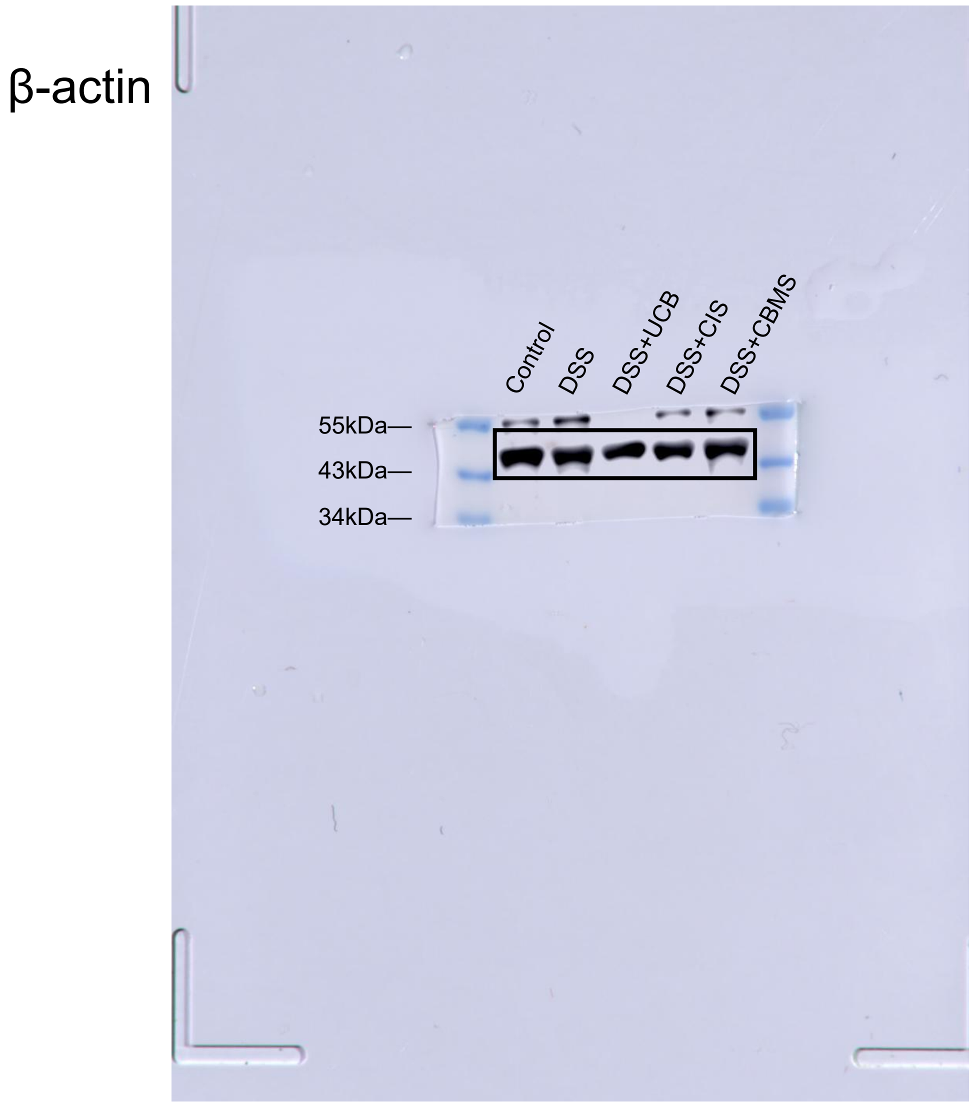

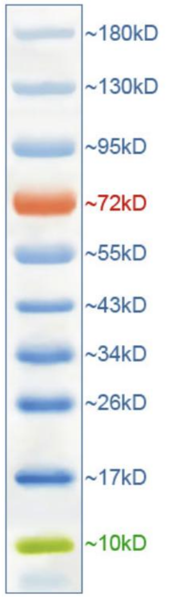

4-20% SDS-PAGE

Uncropped gels for Westem Blots in Figure 5 of p-NF-kB

p-NF-kB

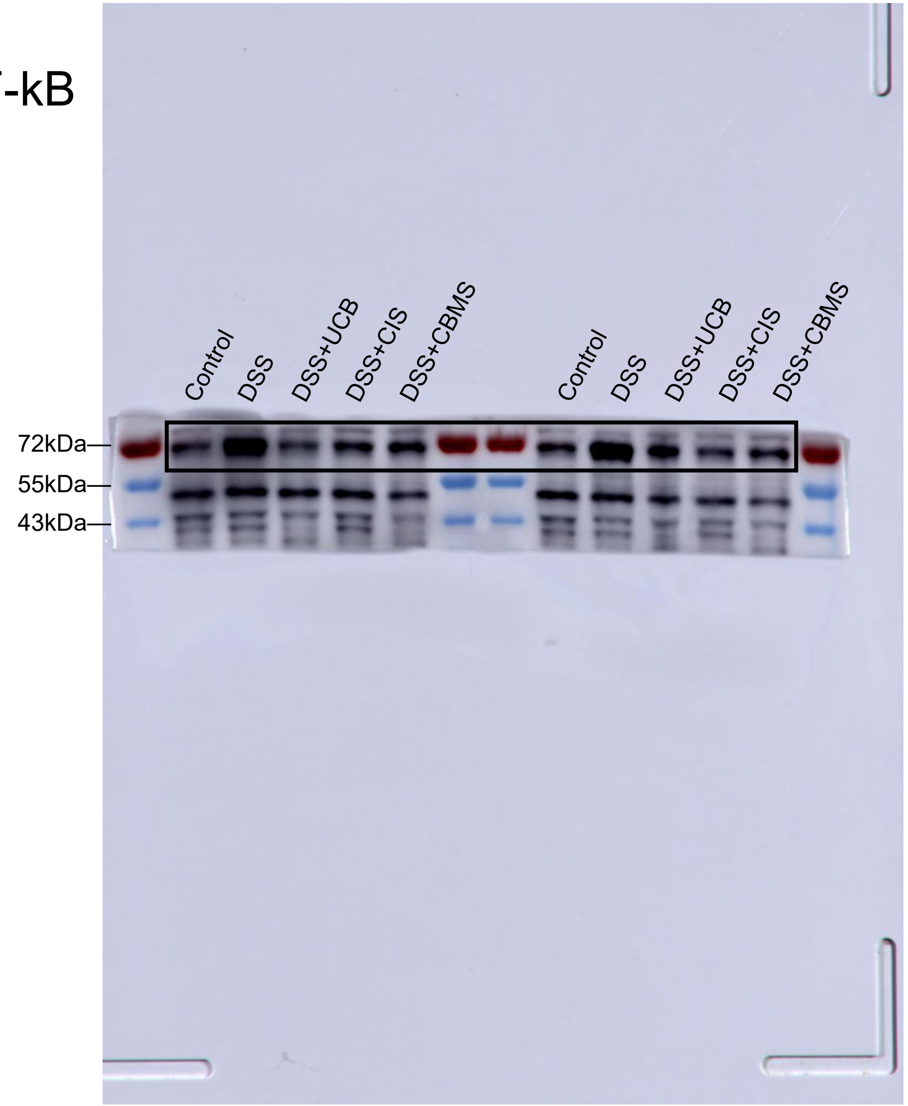

β-actin

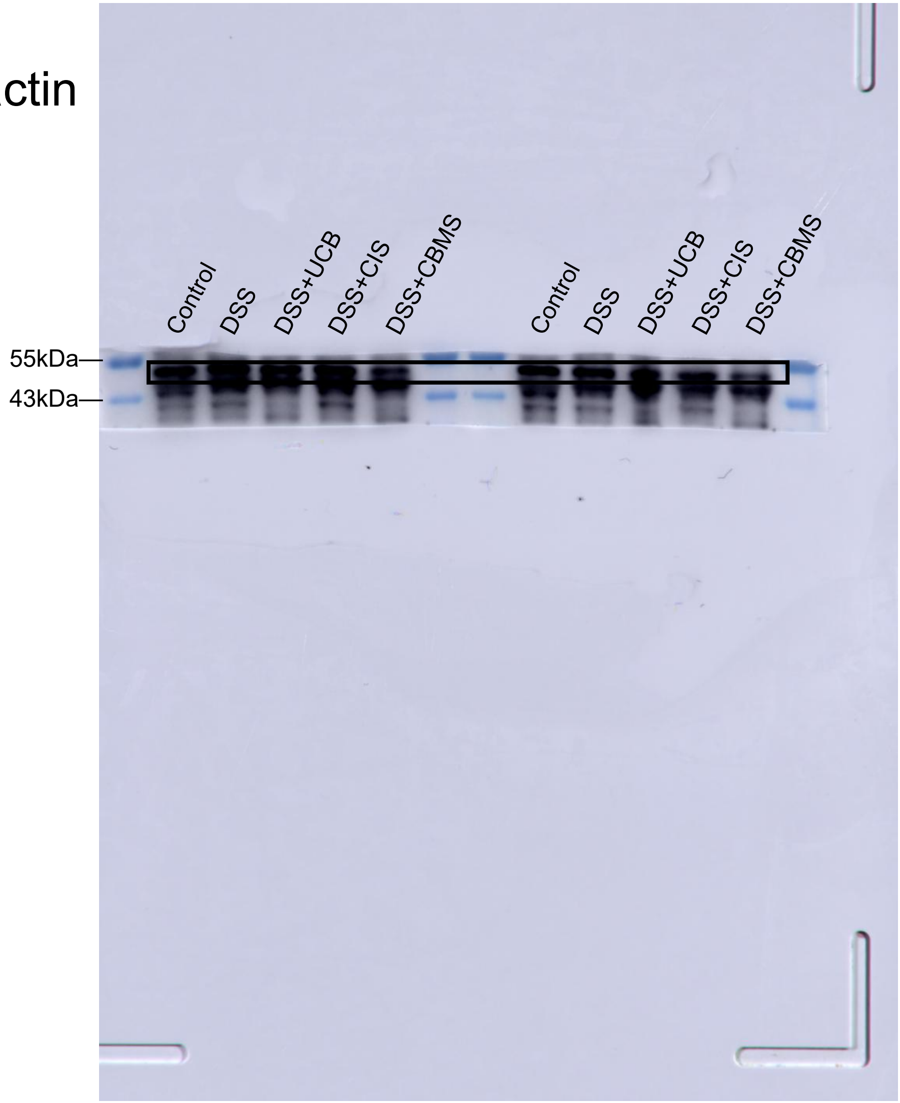

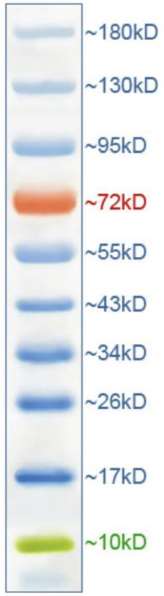

4-20% SDS-PAGE

Uncropped gel from repeated Western blot analysis of p-NF-kB

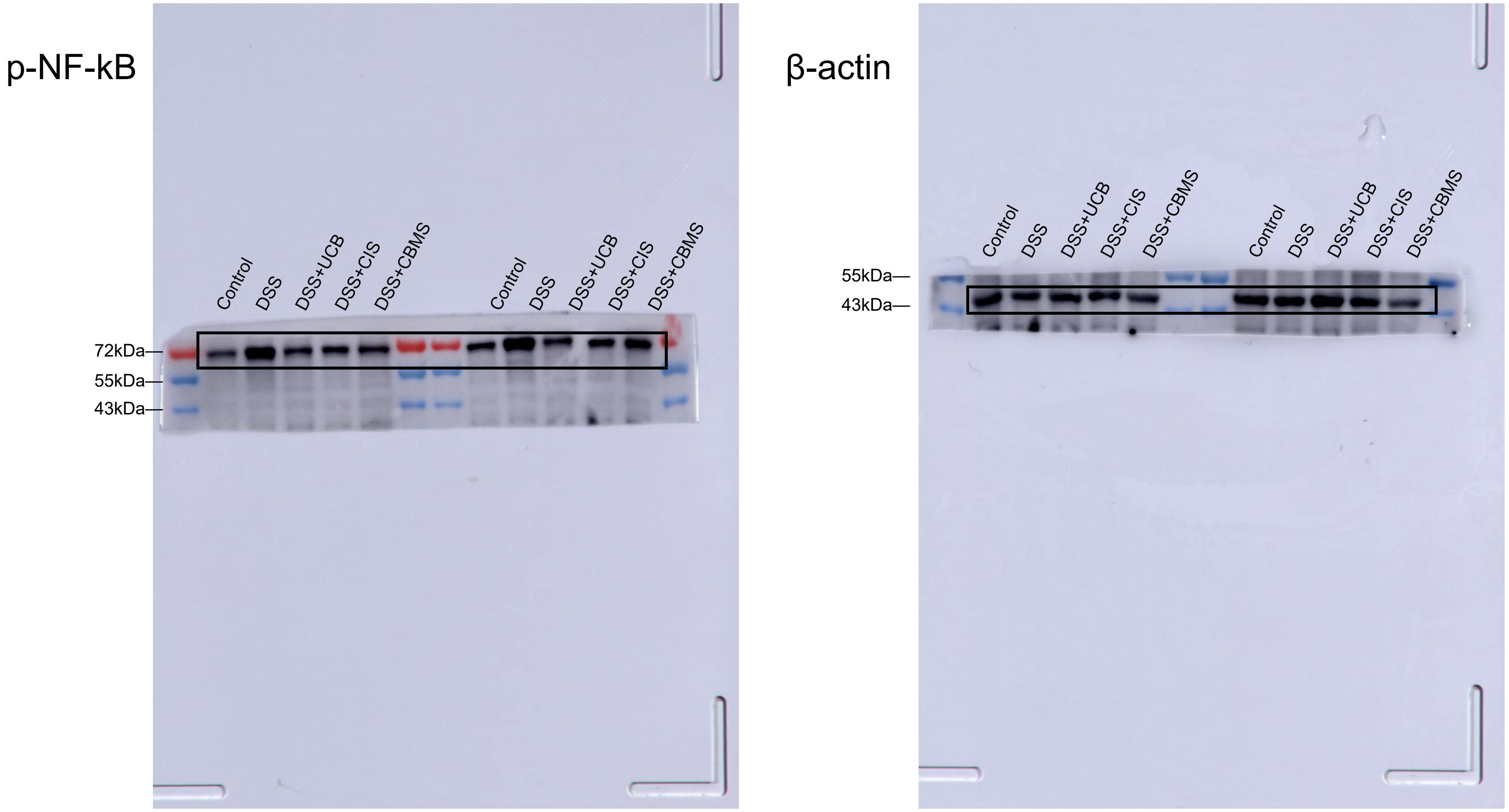

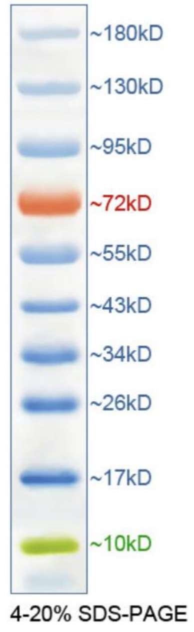

Uncropped gels for Westem Blots in Figure 5 of NF-kB

NF-kB

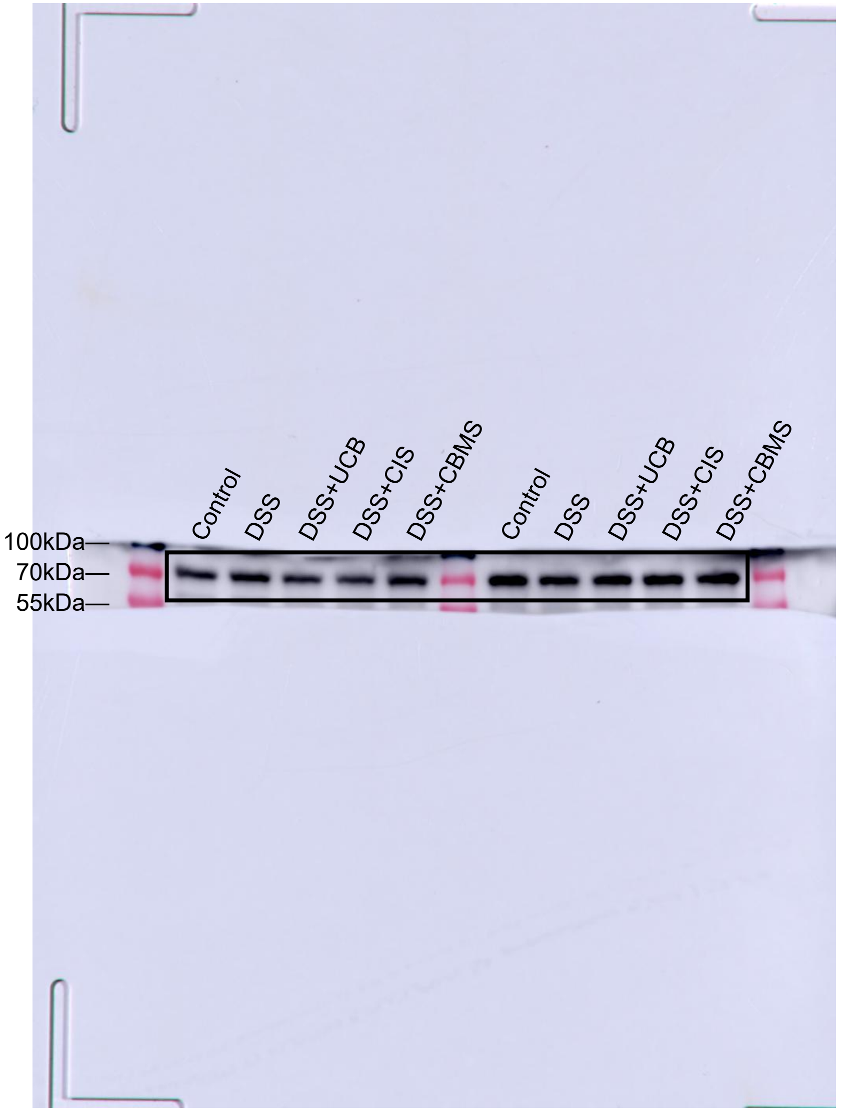

β-actin

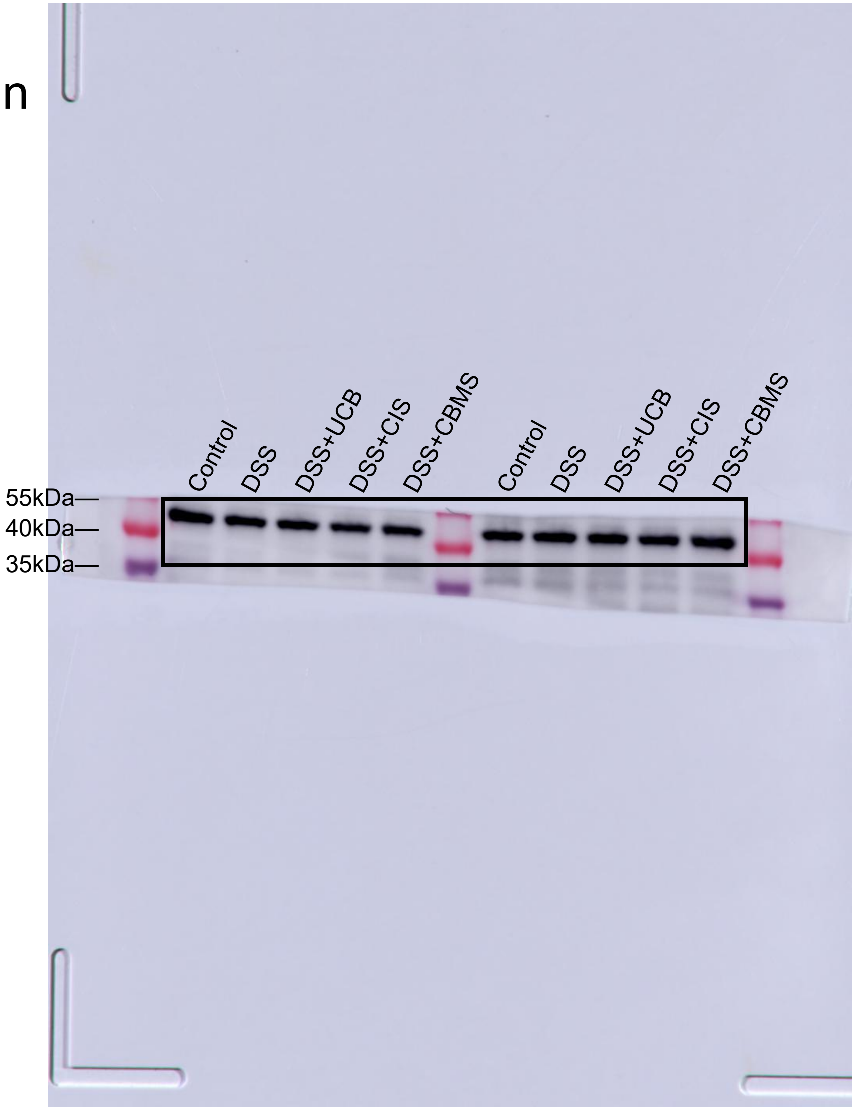

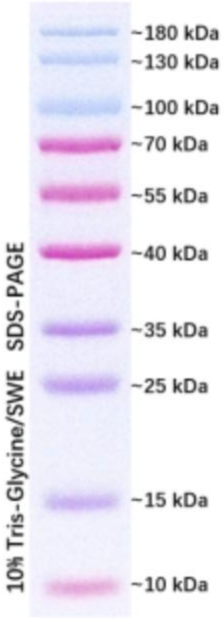

Uncropped gel from repeated Western blot analysis of NF-kB

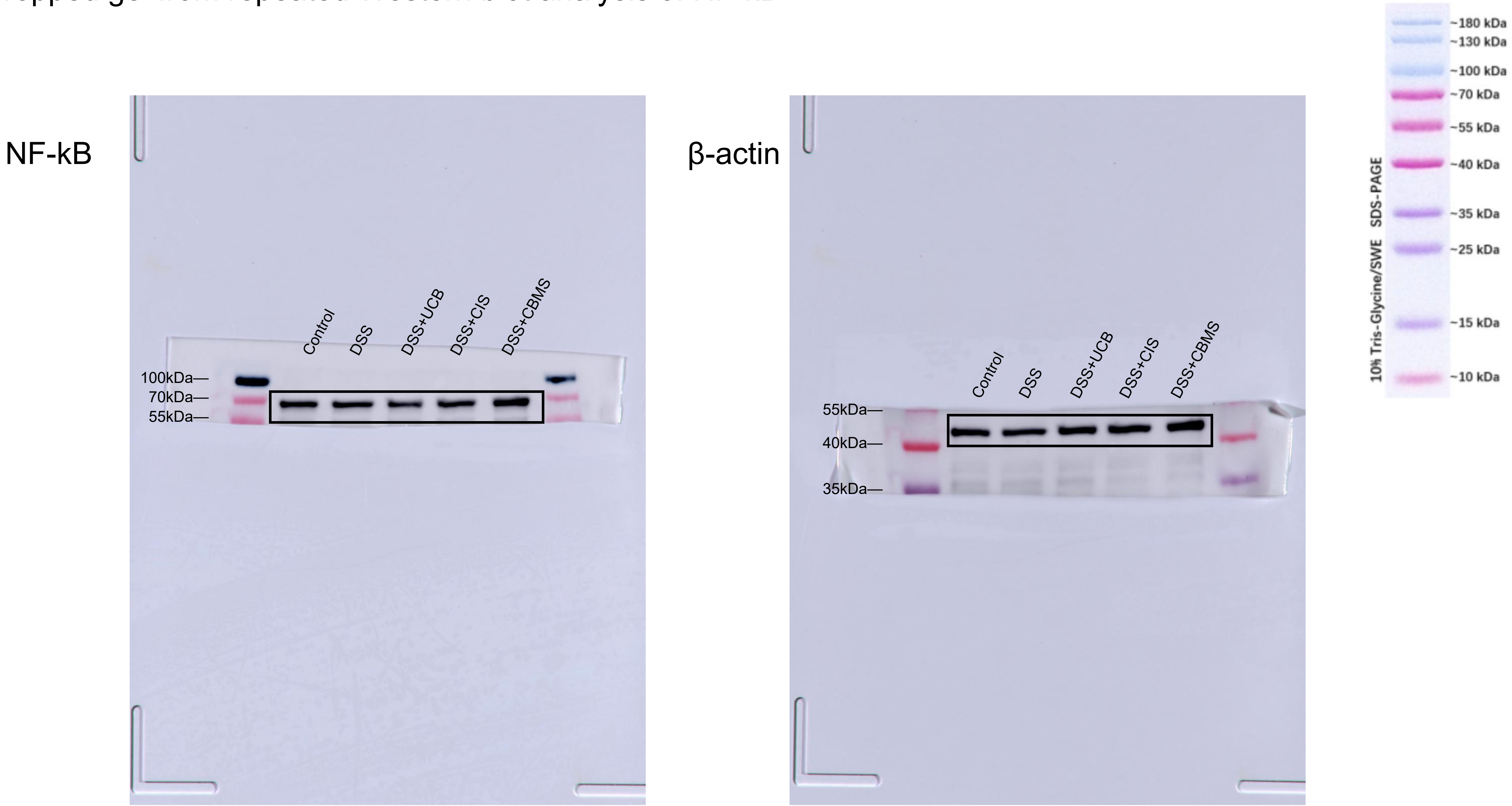

Uncropped gel from repeated Western blot analysis of NF-kB

NF-kB

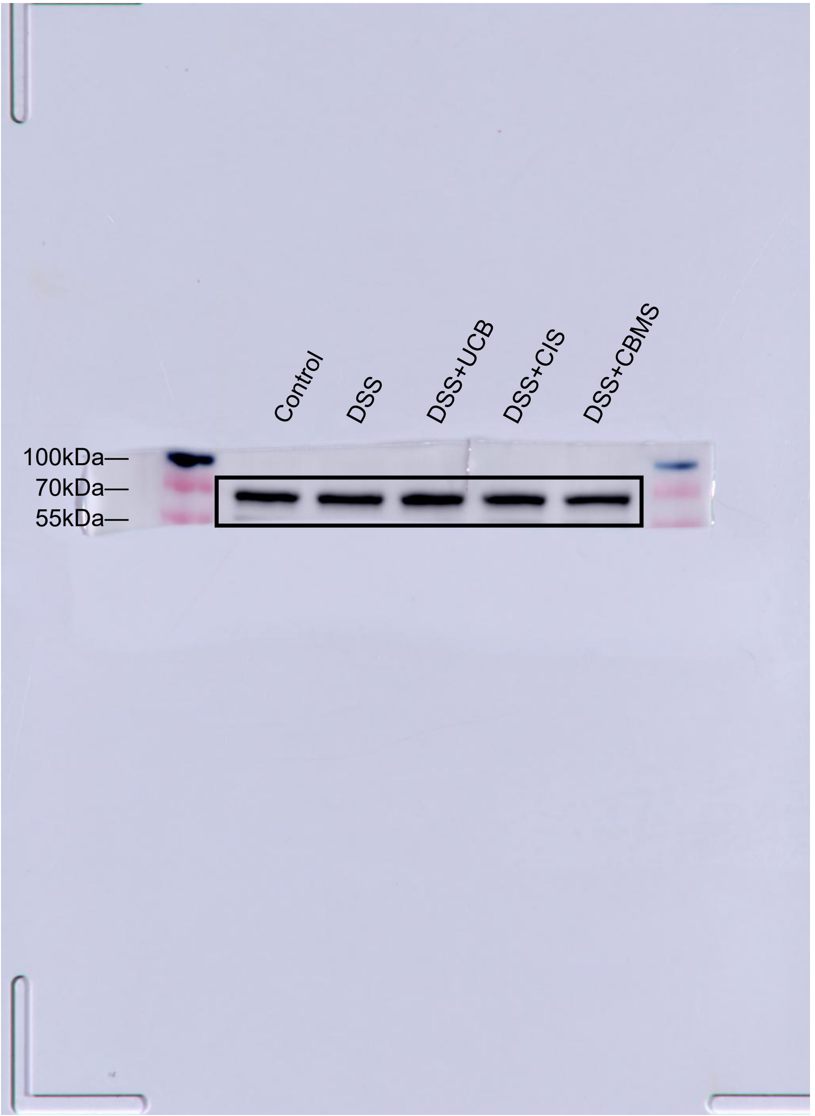

β-actin

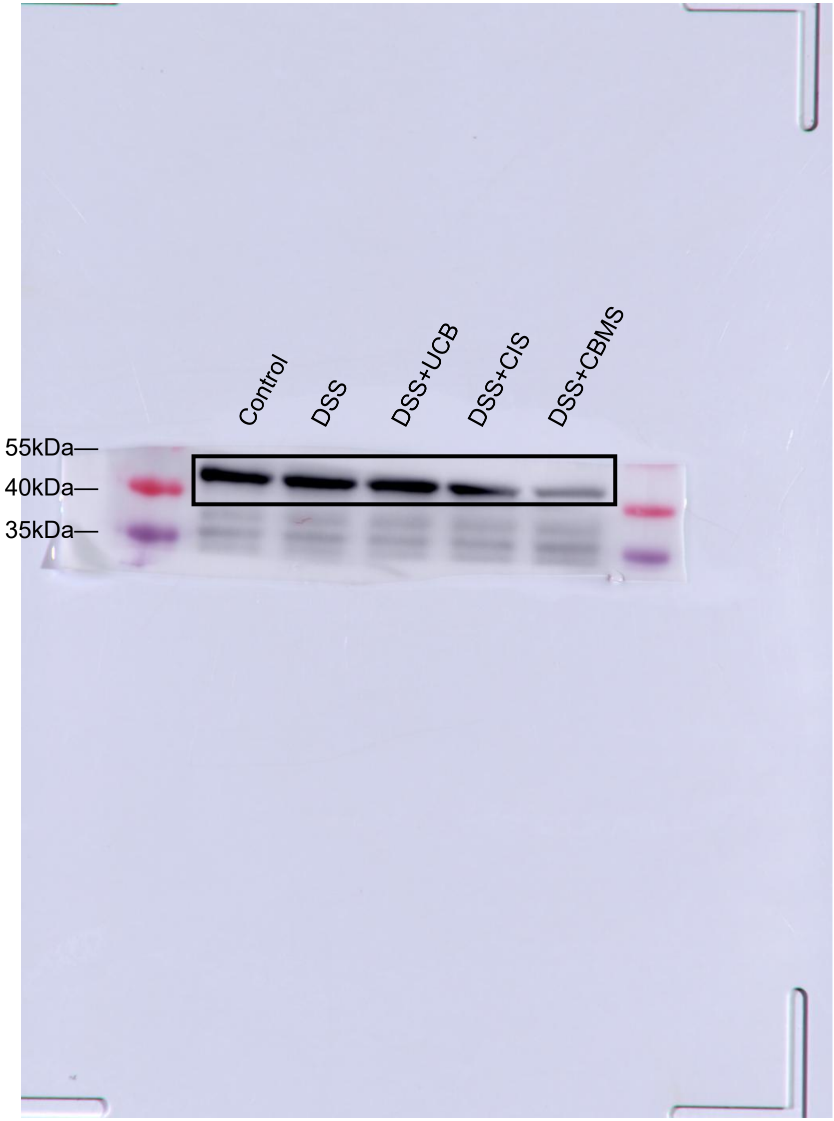

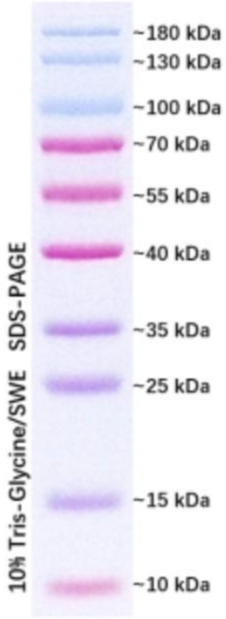

Uncropped gels for Westem Blots in Figure 5 of IL-6

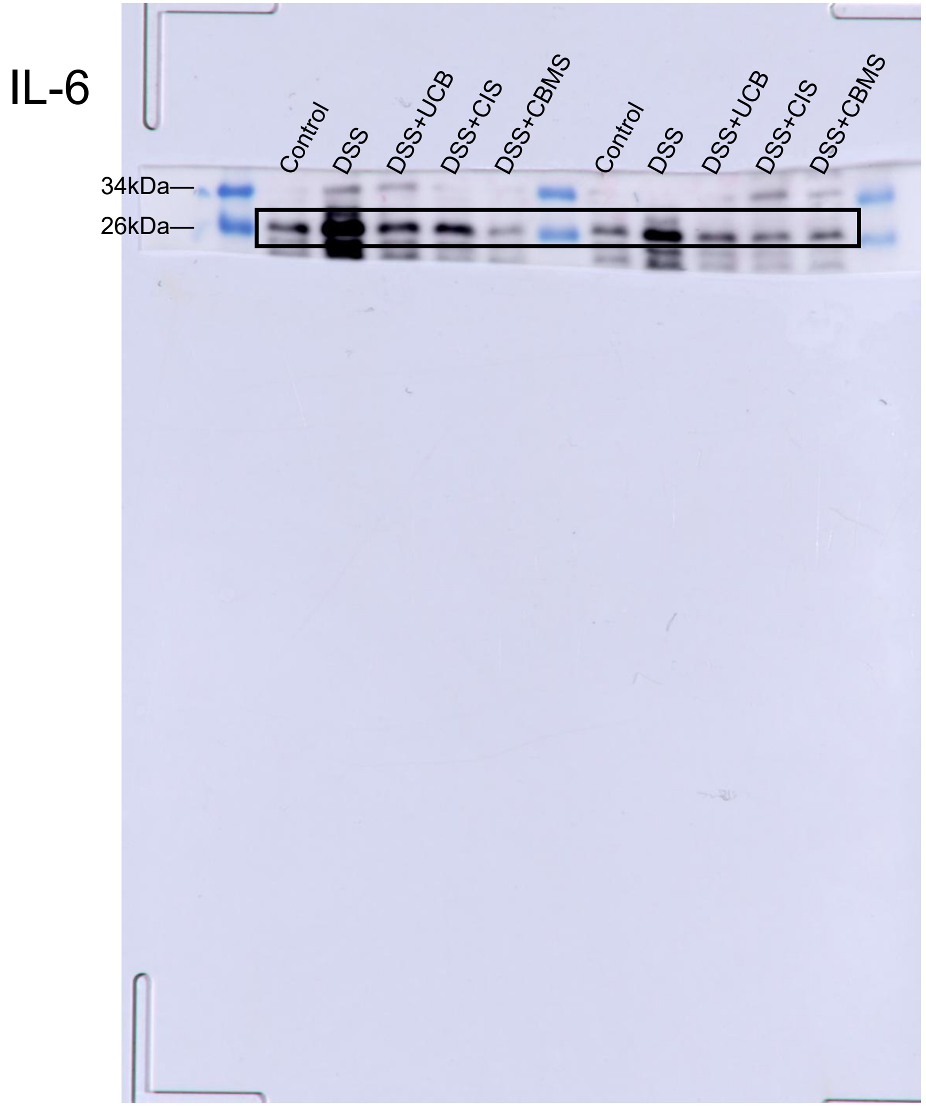

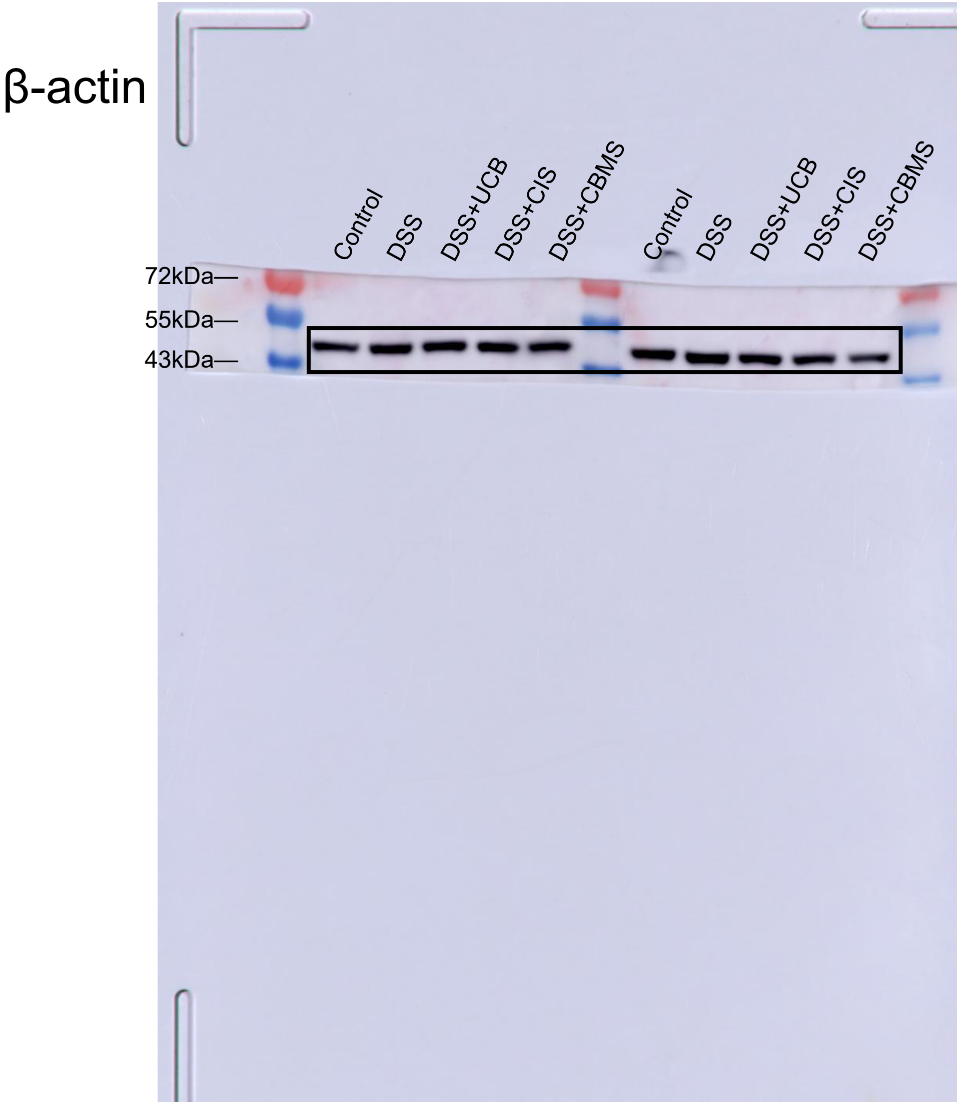

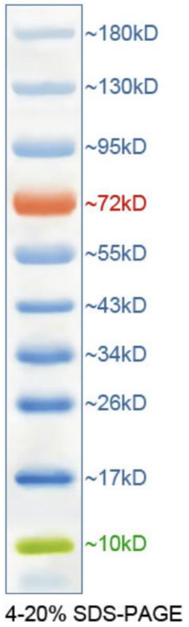

Uncropped gel from repeated Western blot analysis of IL-6

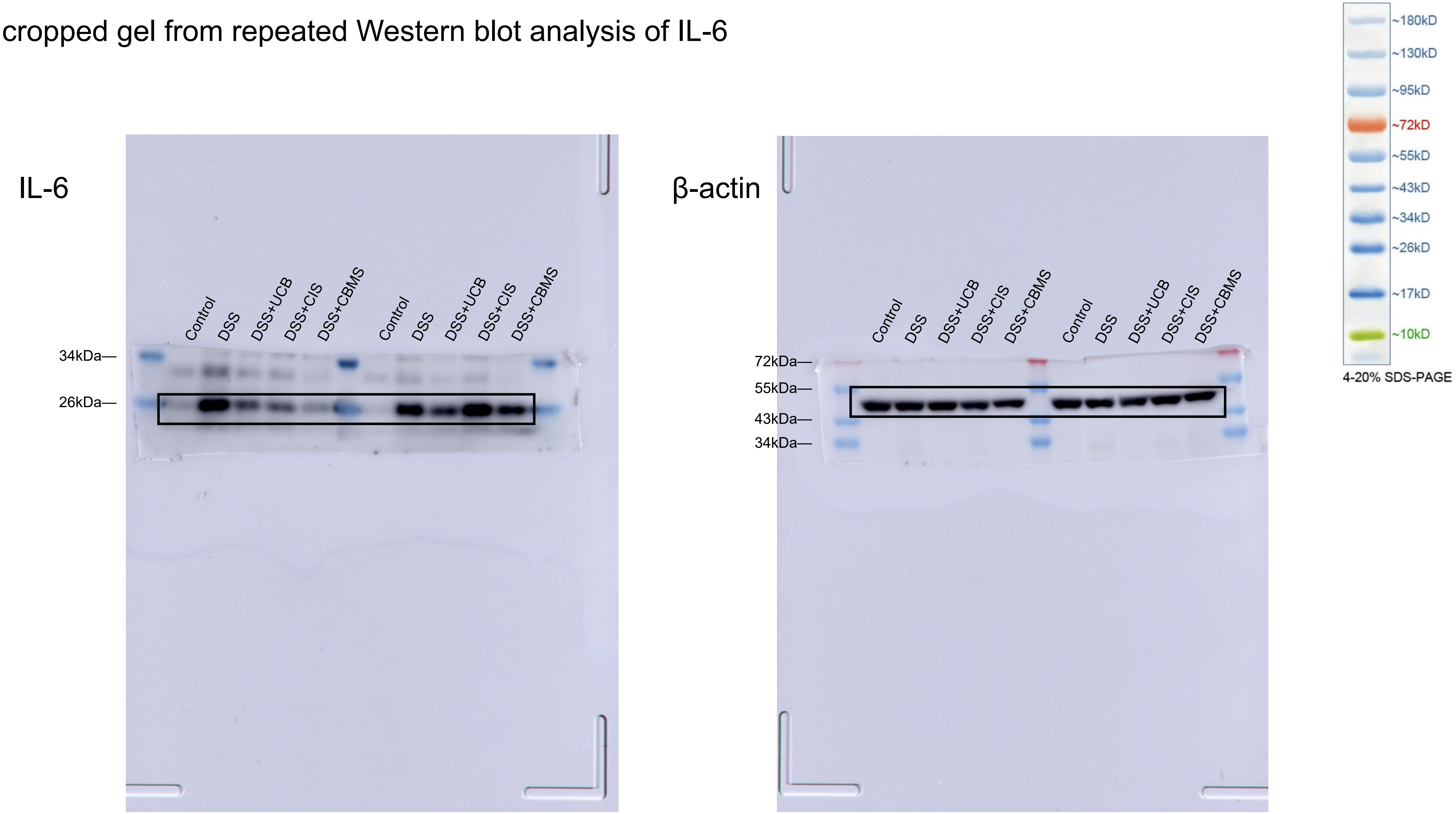

Uncropped gels for Westem Blots in Figure 5 of IL-10

IL-10

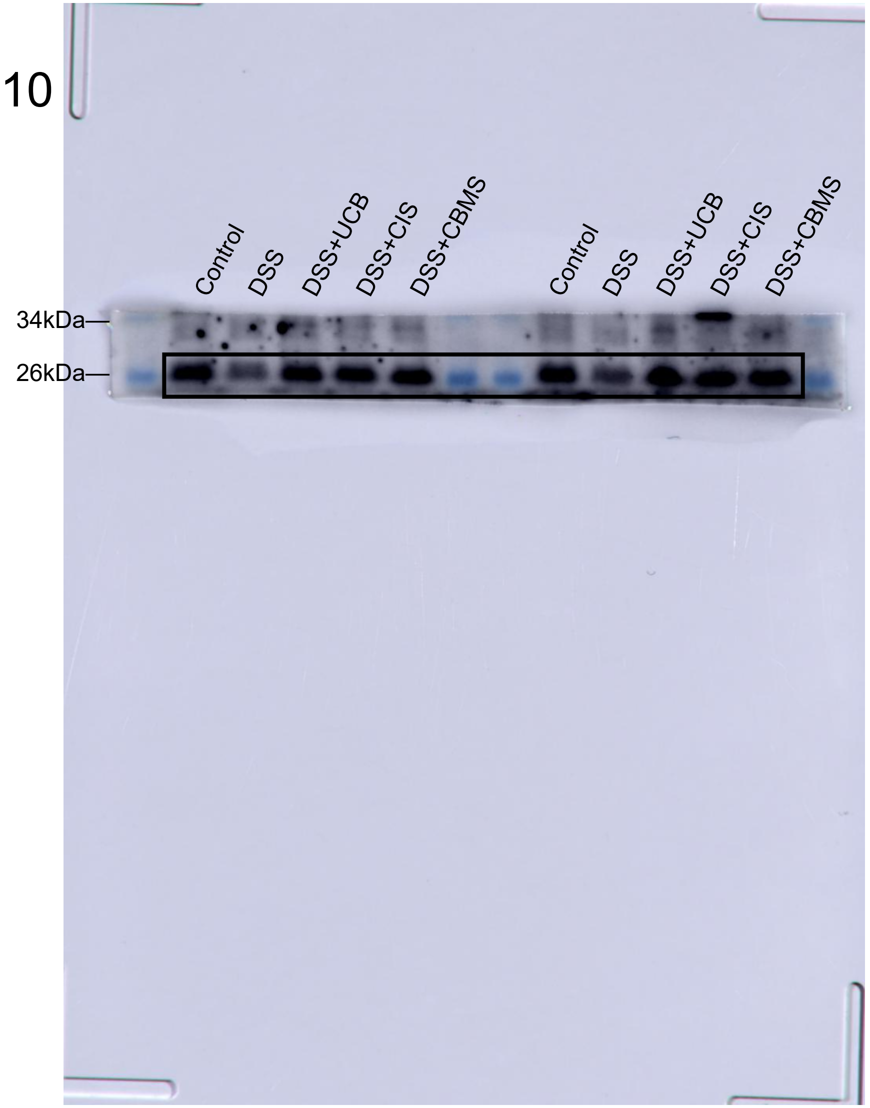

β-actin

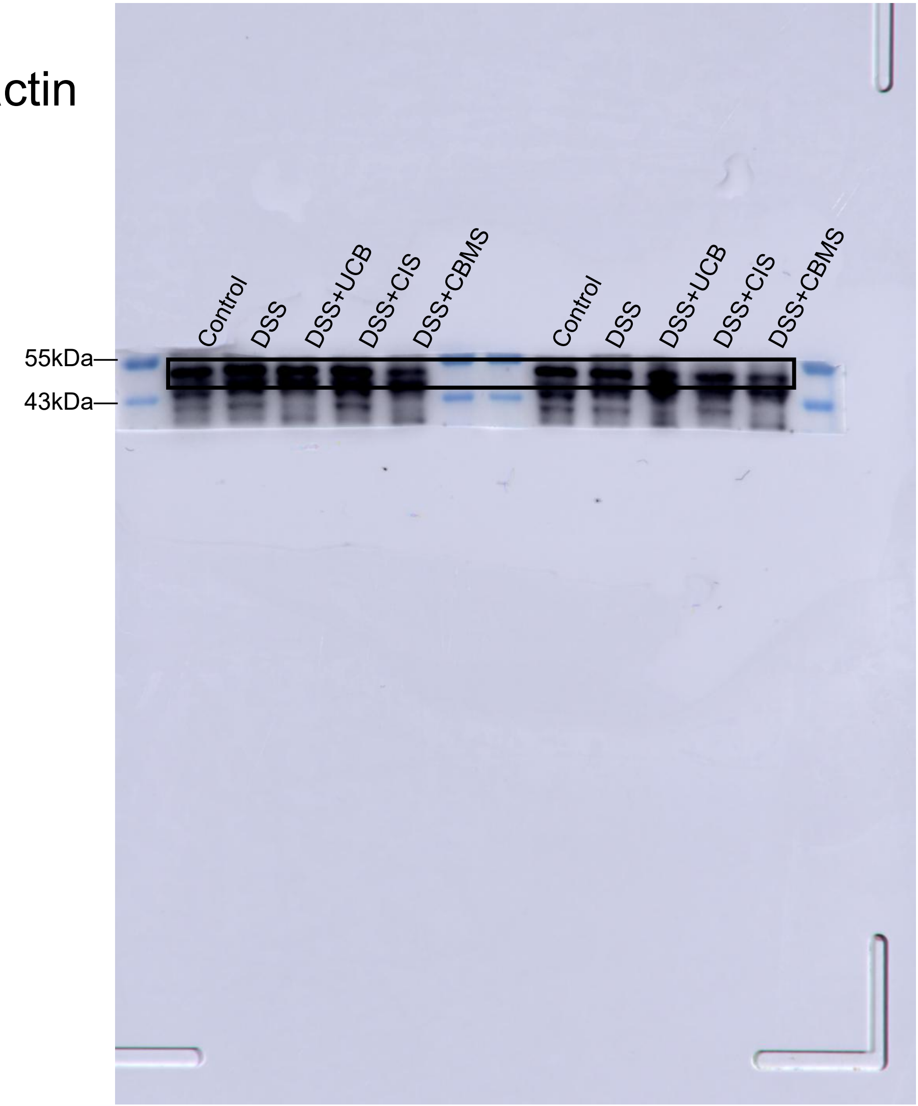

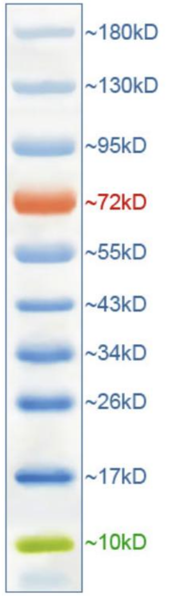

4-20% SDS-PAGE

Uncropped gel from repeated Western blot analysis of IL-10

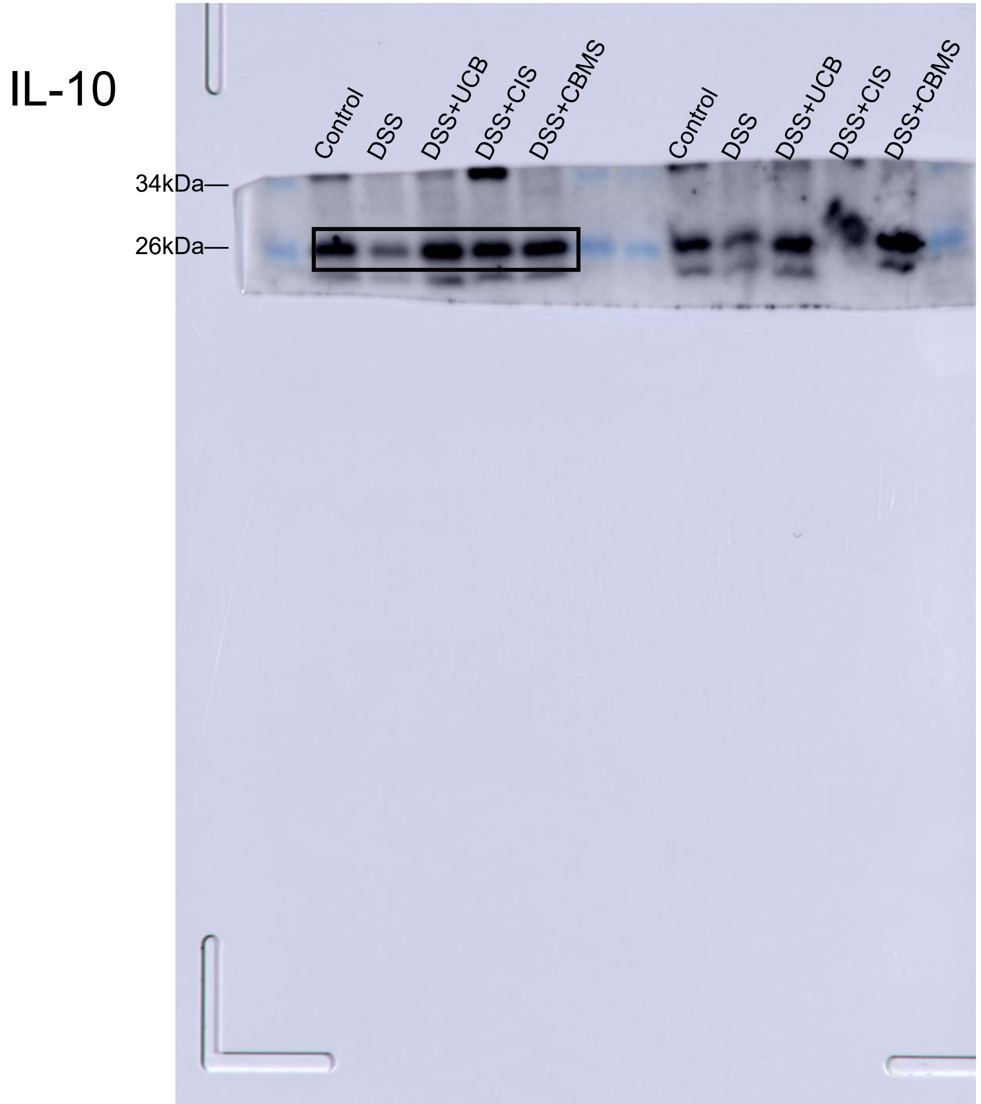

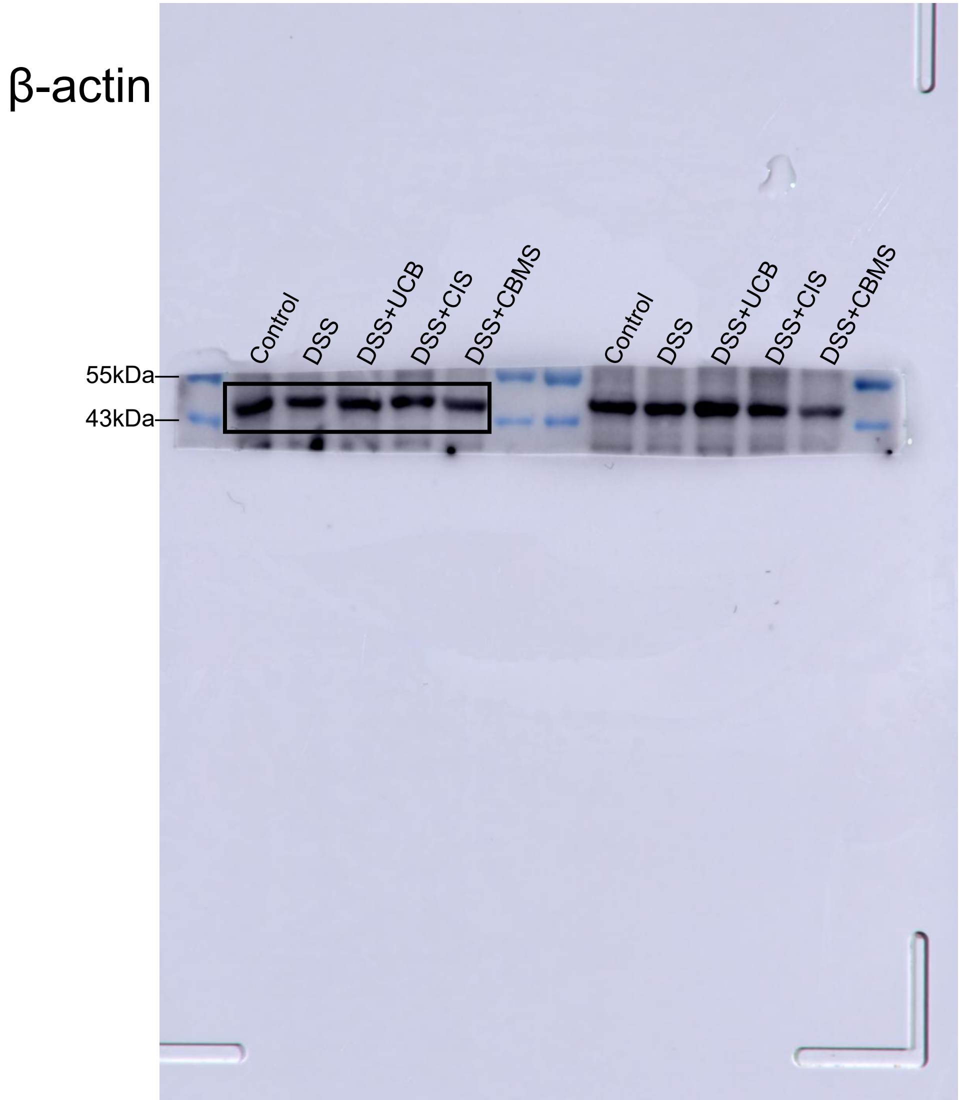

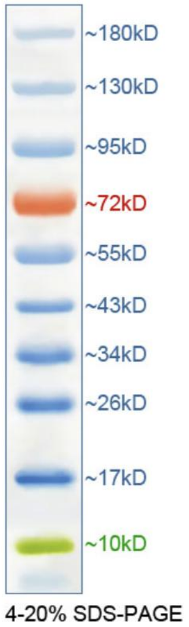

Supplement: Supplementary file 1 [file biomolecules-16-01140-s001.zip › Uncropped gels for Westem Blots.pdf]
